# Decomposition of Intermolecular Interactions in the Crystal Structure of Some Diacetyl Platinum(II) Complexes: Combined Hirshfeld, AIM, and NBO Analyses

**DOI:** 10.3390/molecules21121669

**Published:** 2016-12-06

**Authors:** Saied M. Soliman, Assem Barakat

**Affiliations:** 1 Department of Chemistry, Rabigh College of Science and Art, King Abdulaziz University, P.O. Box 344, Rabigh 21911, Saudi Arabia; 2 Department of Chemistry, College of Science, King Saud University, P.O. Box 2455, Riyadh 11451, Saudi Arabia; ambarakat@ksu.edu.sa; 3 Department of Chemistry, Faculty of Science, Alexandria University, P.O. Box 426, Ibrahimia, Alexandria 21321, Egypt

**Keywords:** diacetyl platinum(II), topology, Hirshfeld, AIM, NBO

## Abstract

Intermolecular interactions play a vital role in crystal structures. Therefore, we conducted a topological study, using Hirshfeld surfaces and atom in molecules (AIM) analysis, to decompose and analyze, respectively, the different intermolecular interactions in six hydrazone-diacetyl platinum(II) complexes. Using AIM and natural bond orbital (NBO) analyses, we determined the type, nature, and strength of the interactions. All the studied complexes contain C-H⋯O interactions, and the presence of bond critical points along the intermolecular paths underlines their significance. The electron densities (ρ(r)) at the bond critical points (0.0031–0.0156 e/a_0_^3^) fall within the typical range for H-bonding interactions. Also, the positive values of the Laplacian of the electron density (∇^2^ρ(r)) revealed the depletion of electronic charge on the interatomic path, another characteristic feature of closed-shell interactions. The ratios of the absolute potential energy density to the kinetic energy density (|*V*(r)|/*G*(r)) and ρ(r) are highest for the O2⋯H15-N3 interaction in [Pt(COMe)_2_(2-pyCMe=NNH_2_)] (1); hence, this interaction has the highest covalent character of all the O⋯H intermolecular interactions. Interestingly, in [Pt(COMe)_2_(H_2_NN=CMe-CMe=NNH_2_)] (3), there are significant N-H⋯Pt interactions. Using the NBO method, the second-order interaction energies, *E*^(2)^, of these interactions range from 3.894 to 4.061 kJ/mol. Furthermore, the hybrid Pt orbitals involved in these interactions are comprised of d_xy_, d_xz_, and s atomic orbitals.

## 1. Introduction

In a crystal, the molecules are packed in a unique pattern held together by weak and strong intermolecular interactions. These interactions strongly affect each other [1] where small changes in the molecular structure produce significant changes in the crystal structure. In general, there is no clear relationship between the molecular structure and the crystal structure. One of the most common intermolecular interactions is with hydrogen bonds, which play an important role in crystal engineering, and hence, the role of hydrogen bonding in a variety of molecules and crystals has been investigated [1].

The theory of atoms in molecules (AIM) proposed by Bader offers a simple method to understand the various intermolecular interactions in molecular systems [2]. The AIM theory yields significant information about the changes in electron distribution due to bond and complex formation. The concepts of chemical bonding and bond strength can be explained using the electron density distribution functions [2,3], obtained from the AIM theory. In this topological analysis, the electron density description of chemical bonding is made using bond paths and bond critical points (BCP). The BCP is a point between two interacting atoms where the gradient of the electron density is zero, indicating a significant interaction between these atoms. For hydrogen bonds, the presence of a BCP on the hydrogen bond path indicates the presence of a hydrogen bond. In addition, topological descriptors such as the electron density (ρ(r)) and the Laplacian of the electron density (∇^2^ρ(r)) at the BCP can be obtained from the AIM theory. These descriptors and others have been used to characterize the strength of hydrogen bonds in various molecular systems. Furthermore, these descriptors can be employed to distinguish between covalent and ionic bonding, hydrogen bonding, and van der Waals (vdW) interactions [4]. In addition, natural bond orbital (NBO) analysis gives another route for the study of intermolecular interactions within molecular systems.

Platinum(II) complexes have great importance in the field of cancer chemotherapy [5,6,7]. For example, cisplatin and carboplatin are the most common Pt-based drugs used for cancer treatment. These medications have almost 100% cure rate for the treatment of ovarian, testicular, and bladder cancers [6,7]. Because of the importance of Pt compounds in cancer therapy, many Pt-based anticancer drugs have been synthesized and have entered clinical use [8,9,10,11,12,13]. In this study, the significant intermolecular contacts obtained from Hirshfeld analyses of the solid-state crystal structures of six structurally related hydrazone-diacetyl platinum(II) complexes were investigated, and AIM and NBO analyses were used to understand the type, nature, and strength of these interactions. We placed particular focus on the characterization of hydrogen-bonding interactions.

## 2. Results and Discussion

### 2.1. Hirshfeld Analysis

Molecular Hirshfeld surfaces of molecules in a crystal structure are constructed based on the electron distribution, which is calculated as the sum of spherical atom electron densities [14,15,16,17,18,19,20,21,22]. The Hirshfeld (HF) surface is unique [23,24,25,26] for each crystal. The properties of the surface yield information about the intermolecular interactions in the crystal. Each point on the Hirshfeld surface represents two distances: (1) the distance from this point to the nearest external nucleus (*d*_e_) and (2) the distance to the nearest internal nucleus (*d*_i_). Graphical plots of the molecular Hirshfeld surfaces are mapped with the normalized contact distance (*d*_norm_), and these indicate regions of important intermolecular interactions [14,15,16,17,18,19,20,21,22,27,28]. The value of the *d*_norm_ is represented by red, white, or blue when the intermolecular contacts are shorter, equal, or longer to the vdW separation, respectively. The combination of *d*_e_ and *d*_i_ in the form of a 2D fingerprint plot gives a summary of the intermolecular contacts in the crystal [14,23,26,29]. The fingerprint plots can be decomposed to highlight particular atom pair close contacts [23,28] (Figure S1). This decomposition enables separation of contributions from different interaction types, which overlap in the full fingerprint. Also, it provides a valuable quantitative analysis of the intermolecular interactions occurring in the crystal structure. 

**Figure 1:**
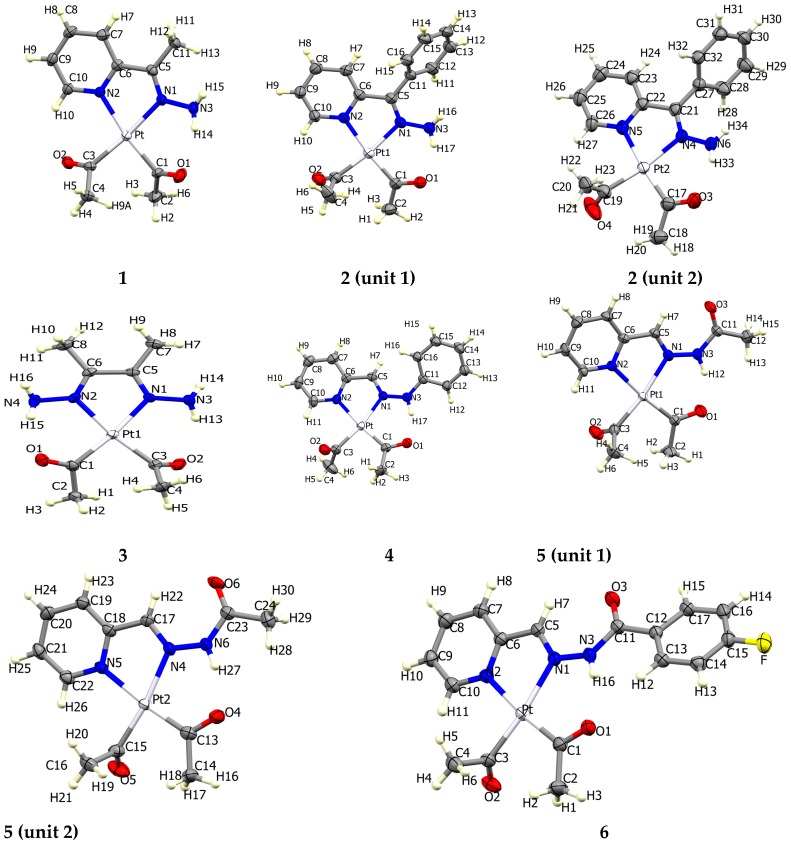
Atomic numbering in the six Pt complexes (front view).

**Figure 2:**
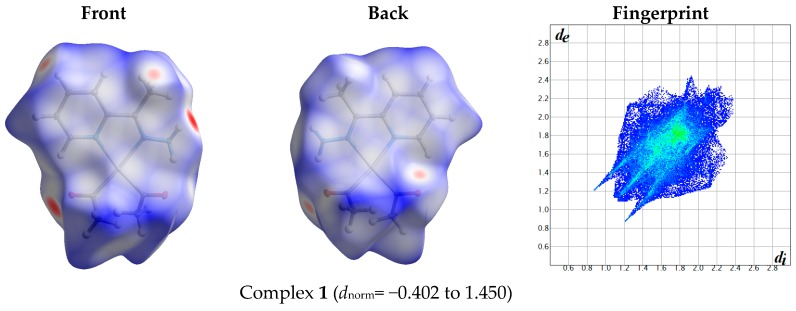

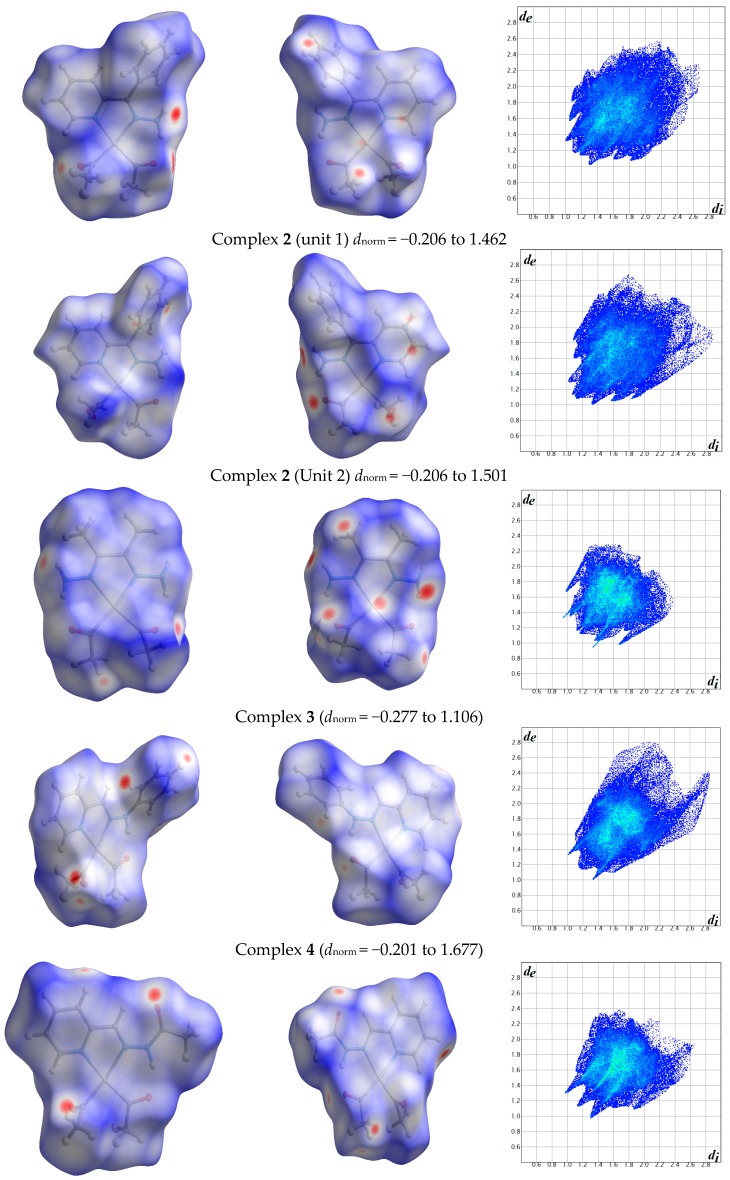

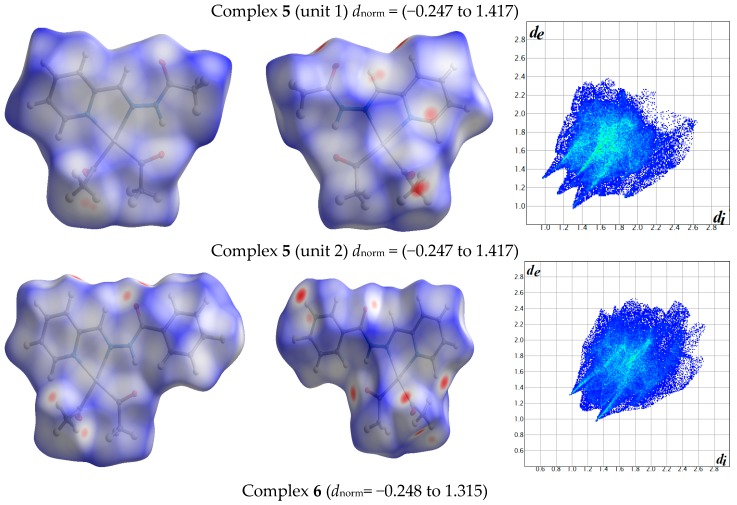
Hirshfeld surfaces and full fingerprint plots of the studied complexes; front views are referred to Figure 1.

**Figure 3:**
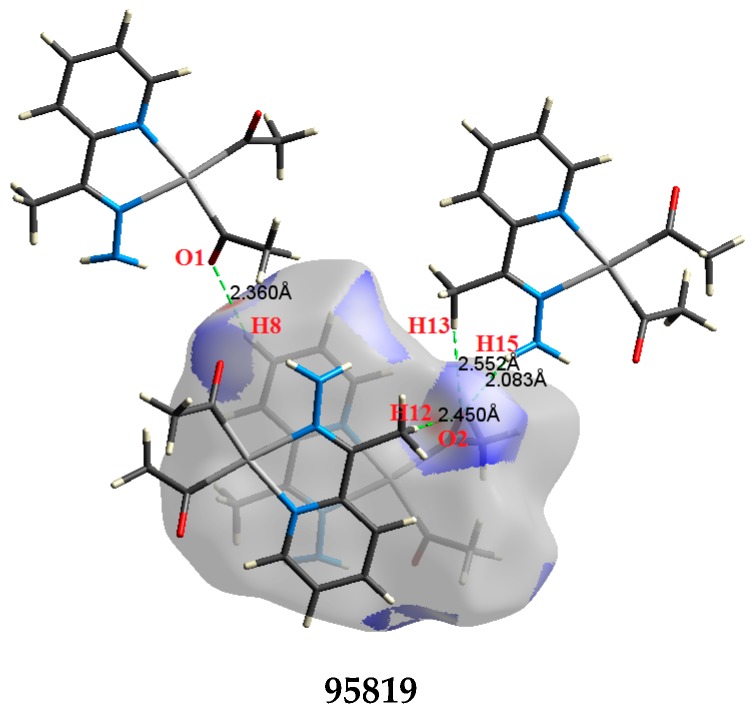
The most important intermolecular contacts in complex **1**.

**Figure 4:**
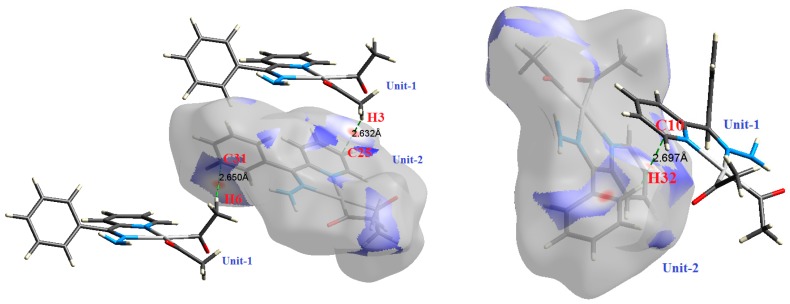

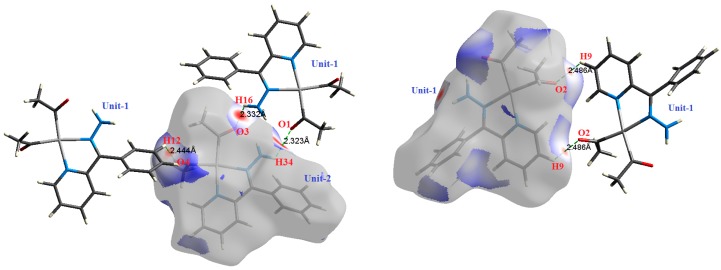
The most important intermolecular contacts in complex **2**.

**Figure 5:**
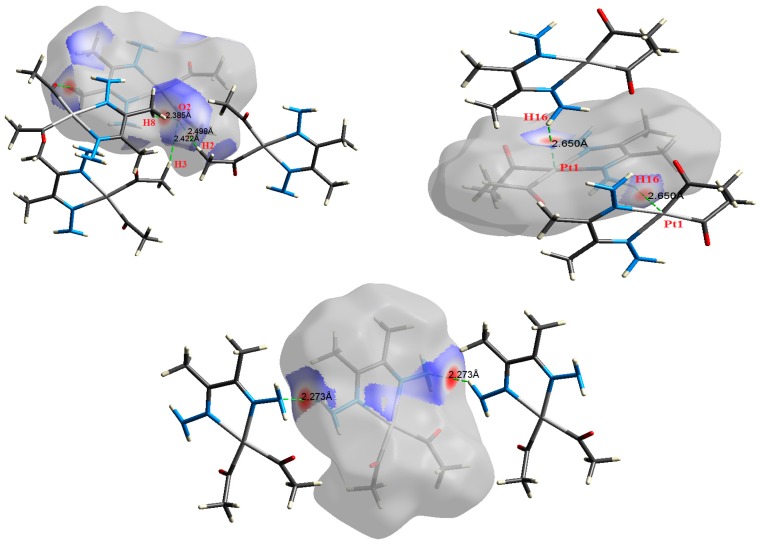
The most important intermolecular contacts in complex **3**.

**Figure 6:**
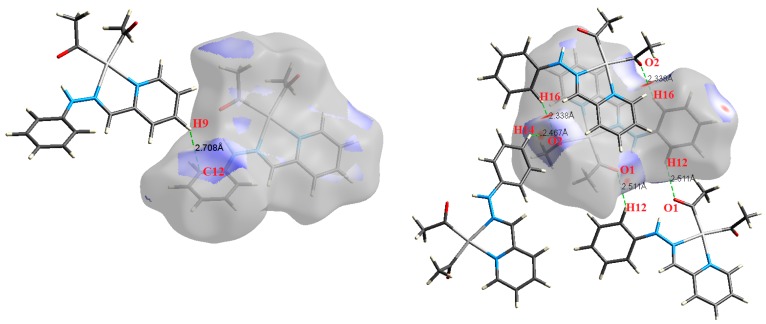

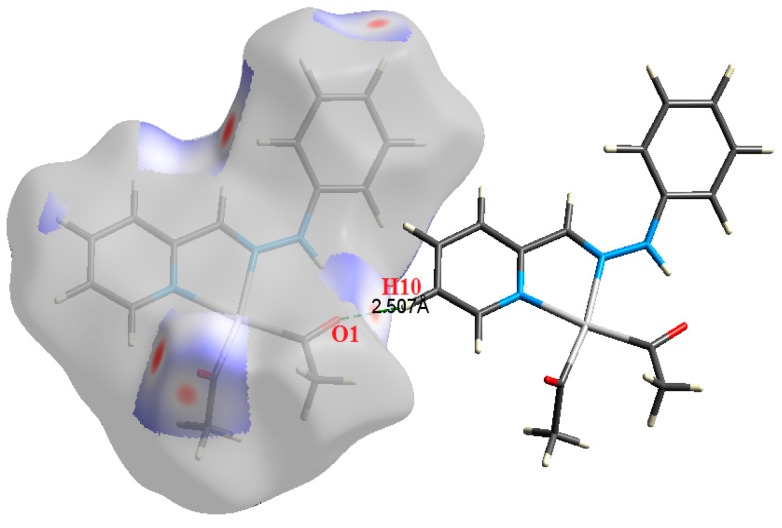
The most important intermolecular contacts in complex **4**.

**Figure 7:**
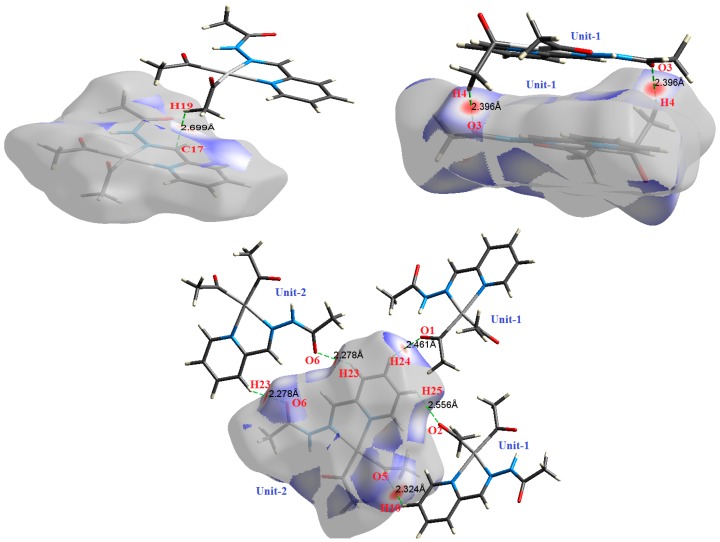
The most important intermolecular contacts in complex **5**.

**Figure 8:**
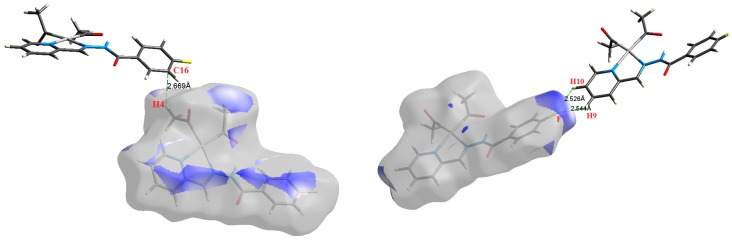

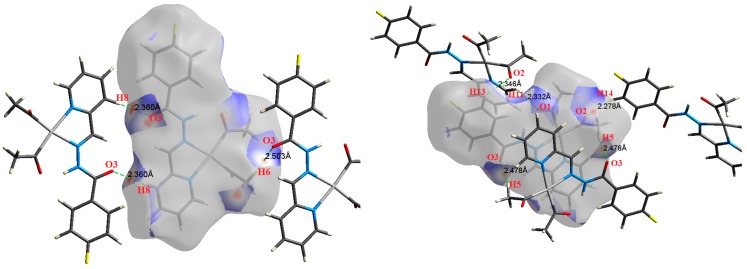
The most important intermolecular contacts in complex **6**.

The atom numbering schemes according to the X-ray structures of the studied Pt-complexes are shown in Figure 1. The front and back views of the Hirshfeld surfaces together with the fingerprint plots of the six Pt complexes are shown in Figure 2. The decomposed fingerprint maps for all possible interactions are given in the Supplementary Data (Figure S1). The most significant intermolecular interactions in the crystal lattice of the studied complexes are listed in Table 1 and shown in Figure 3, Figure 4, Figure 5, Figure 6, Figure 7 and Figure 8. In the same table, the minimum contact distances between two interacting atoms are given in brackets.

Many common intermolecular interactions, such as H⋯H, H⋯C, and H⋯O contacts, play a crucial role in the crystal packing of these Pt complexes. The H⋯H intermolecular contacts make the largest contribution (34.9%–57.0%) in the fingerprint maps of all complexes, and it is believed that these intermolecular interactions play a major role in crystal lattice stability [30]. The minimum H⋯H contact distances are 2.230, 2.206, 2.288, 2.320, 2.017, and 2.250 Å for complexes **1**–**6**, respectively. In all complexes, the contact distances are more than twice the van der Waals radius of a hydrogen atom, except for those of complex **5**, where the minimum H⋯H intermolecular separation is slightly less than twice the van der Waals radius of a hydrogen atom.

O⋯H intermolecular interactions are another type of close contact that makes a large contribution to the crystal lattice of all the studied complexes. These structures having shorter intermolecular O⋯H contact distances than the sum of the van der Waals radii are shown in Figure 3, Figure 4, Figure 5, Figure 6, Figure 7 and Figure 8. The O⋯H% is highest for complexes **3** and **5**. Three types of H⋯O interactions were detected. The N-H⋯O interactions have the shortest hydrogen bond distances. The O⋯H distances for these interactions are 2.083 Å and 2.323–2.332 Å for complexes **1** and **2**, respectively. The other complexes did not show this type of interaction. Moreover, the other two types of C-H⋯O interactions occurred between either the aliphatic or aromatic C-H bonds with the O-atoms of the carbonyl groups of the neighboring molecules. The H⋯C intermolecular contacts also showed high contributions to the overall fingerprint plot, except for complex **3**. H⋯C% is highest in **2**, **4**, and **6,** which contain two phenyl rings, while it is lowest in **3,** which has no aromatic ring system. The most significant C⋯H intermolecular contact distances are shown in Figure 3, Figure 4, Figure 5, Figure 6, Figure 7 and Figure 8. These figures showed that the shortest C⋯H intermolecular distances occurred in **2**. The minimum contact distance in **2** is found to be 2.632 Å, which is less than the van der Waals radii sum of the two elements. It is well known that the shorter the contact distance compared to the van der Waals radii sum, the more significant the intermolecular interaction.

The Hirshfeld surface analysis of complexes **2** and **3** showed the presence of Pt⋯H interactions. In **2**, the Pt atom from one complex interacts with the H31 of the phenyl ring of neighboring complex; the interactions are very weak, as indicated by the longer contact distance (2.901 Å, which is longer than the vdW radii sum). In contrast, for **3**, the Pt atom interacts with the N-H proton from a neighboring complex (2.81 Å). The Pt⋯H distance, in this case, is 2.650 Å, which is less than the sum of the vdW radii. Moreover, complexes **3** and **6** contain N⋯H (2.273 Å) and F⋯H (2.526–2.544 Å) intermolecular interactions, respectively, and these contact distances are shorter than the van der Waals radii sum of the two elements. However, the latter range of values is very close to the vdW sum of the two elements (F and H) and could be considered a weak interaction. Notably, the absence of any significant intermolecular Pt⋯Pt, Pt⋯O, Pt⋯N, and Pt⋯C contacts indicates the monomeric nature of these complexes. Another significant feature of the Hirshfeld surface analysis is the sensitivity of the fingerprint plots to small variations in geometric parameters. Although the asymmetric units of complexes **2** and **5** are very similar, their Hirshfeld surfaces and fingerprint plots are unique. The importance of shape index and curvedness plots is explained in the Supplementary Data (Figure S2).

### 2.2. AIM Study

The quantum theory of atoms in molecules [2] is a popular tool for describing various inter- and intra-molecular interactions. The AIM theory uses topological parameters such as the electron density (ρ(r)), the Laplacian of the electron density (∇^2^ρ(r)), the kinetic energy density *G*(r), the potential energy density *V*(r), and the total electron energy density (*H*(r) = *V*(r) + *G*(r)) at the bond critical point (BCP) of interacting atoms or fragments [31,32,33]. According to Espinosa [34], the interaction energy (*E*_int_) can be estimated using the potential energy density at the BCP as *E*_int_ = 1/2 (*V*(r)). The topological parameters, as well as the calculated interaction energies (*E*_int_) of the different intermolecular interactions observed from the Hirshfeld analysis, are listed in Table 2.

Bader’s theory showed that the topological properties ρ(r) and ∇^2^ρ(r) at the BCP of two hydrogen-bonded atoms are important parameters for investigating these intermolecular interactions. The atomic interactions belong to two general classes: (1) shared interactions, such as covalent and polarized bonds, in which the electronic charge is concentrated on the line of interaction linking the nuclei. For these interactions, ∇^2^ρ(r) <0 and ρ(r) should be ˃10^−1^ a.u. (2) Closed-shell interactions, such as hydrogen bonds and van der Waals interactions, in which the electronic charge is concentrated towards each of the interacting nuclei, deplete the electronic charge at the interatomic surface, and in this case, ρ(r) ≈ 10^−2^ and ∇^2^ρ(r) > 0. Furthermore, the electron density, ρ(r), has been used to measure the degree of covalency of the intermolecular interactions [4]. Consequently, ρ(r) and ∇^2^ρ(r) at the BCP of hydrogen-bonded atoms should be 0.002 ± 0.035 e/a_0_^3^ and 0.024 ± 0.139 e/a_0_^5^, respectively, if a hydrogen bond exists [33].

**Figure 9:**
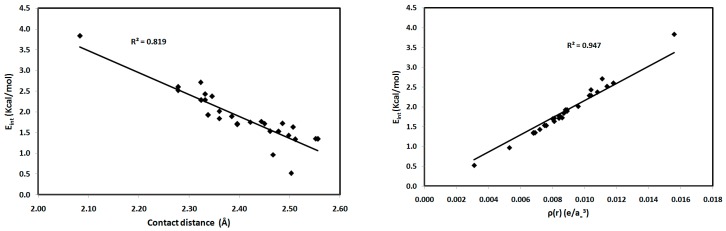
Interaction energies (*E*_int_) versus contact distances (left) and electron density (ρ(r)) (right) for the studied systems.

**Figure 10:**
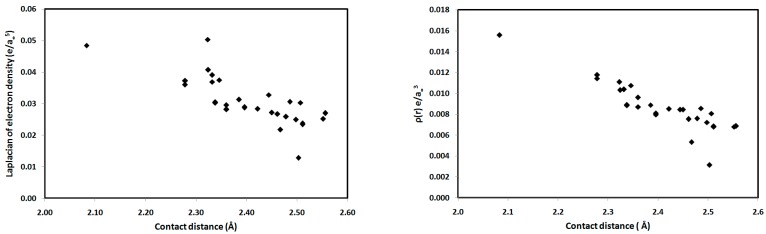
Contact distances versus the topological parameters ∇^2^ρ(r) and ρ(r) for the studied systems.

For the studied systems, the O⋯H hydrogen bonds had low ρ(r) and positive ∇^2^ρ(r) values, typical of hydrogen-bonded closed-shell interactions [35] and satisfying the criteria proposed by Popelier for hydrogen bond formation [36]. As shown in Table 2, the values of ρ(r) at the BCPs range from 0.0031–0.0156 e/a_0_^3^, which falls within the typical range proposed by Popelier [33] for hydrogen-bonding interactions. In addition, the positive values of ∇^2^ρ(r) for all interactions indicate that the electronic charges are depleted along the interatomic path; again, this is characteristic of closed-shell interactions, such as hydrogen bonds. We noted that the O2⋯H15-N3 interaction in **1** has the highest ρ(r) indicating the strongest hydrogen bonding interaction and the highest covalent character of the interactions studied. The hydrogen-bonding interaction energies (*E*_int_) calculated using the potential energy density (*V*(r)) are listed in Table 2. Correlation graphs between the *E*_int_ and ρ(r) are straight lines (Figure 9) with high correlation coefficients (*R*^2^ = 0.947), following the relationship *E*_int_ = 216.2 ρ(r), which agrees well with the equation of Parthasarathi [37]. A graphical plot of the hydrogen bond distance and *E*_int_ gave the expected inverse linear relationship (Figure 9). Stronger hydrogen-bonding interactions with shorter hydrogen-bond distances are usually indicated by higher values of ρ(r) at the BCP, indicating a more covalent hydrogen bond (Figure 9). Moreover, the contact distances plotted against ρ(r) and ∇^2^ρ(r) are shown in Figure 10. There is an inverse relationship between the hydrogen bond length and both ρ(r) and ∇^2^ρ(r). Furthermore, there is a general decrease in the electron density and the strength of the hydrogen bonds with increasing contact distance because the increase in the atomic separation results in reduced orbital overlap, decreasing the electron density along the bond path. 

According to Rozas et al. [38], interactions may be classified based on the total electronic energy density, *H*(r) and ∇^2^ρ(r), which are indicative of the strength and the degree of covalency of the hydrogen bonds. The values ∇^2^ρ(r) > 0 and *H*(r) > 0 are indicative of weak hydrogen bonds that are mainly electrostatic in nature. In contrast, medium strength hydrogen bonds are characterized by ∇^2^ρ(r) > 0 and *H*(r) < 0, while, for strong hydrogen bonds, both ∇^2^ρ(r) and H(r) are less than zero. Moreover, Espinosa et al. [39] used the ratio of the absolute potential energy density to the kinetic energy density (|*V*(r)|/*G*(r)) to classify the bonding interactions. In their study, closed-shell interactions have a ratio of |*V*(r)|/*G*(r) < 1, while shared interactions have a ratio of |*V*(r)|/*G*(r) > 2. Bonded interactions with |*V*(r)|/*G*(r) ratios between 1 and 2 are considered intermediate between these two extremes. As shown in Table 2, the absolute value of the potential energy density is, generally, smaller than the kinetic energy density; this results in |*V*(r)|/*G*(r) ratios that range from 0.686 to 0.943 and are, in general, less than 1. In addition, most interactions have ∇^2^ρ(r) > 0 and *H*(r) > 0 which are the typical characteristics of weak interactions, except for the N-H⋯O hydrogen bond in complex **1**. Therefore, based on the values ∇^2^ρ(r) > 0 and (*H*(r)) < 0, the N-H⋯O interaction in **1** is a medium strength hydrogen bond with partially covalent character. In addition, the N-H⋯O hydrogen bond has a |*V*(r)|/*G*(r) ratio slightly greater than 1 (1.004). Therefore, the high covalency of this interaction is evident.

Other intermolecular interactions, such as the C⋯H, H⋯F, and C⋯N, showed similar results, but, in general, the values of the ρ(r), ∇^2^ρ(r), and *E*_int_ are lower than those for the H⋯O interactions. In addition, the |*V*(r)|/*G*(r) ratios are very low for these interactions, which also have the lowest interaction energies; consequently, these interactions are considered weak and play a less important role in crystal packing than the previously discussed interactions. In contrast, complex **3** has significant N⋯H interactions which seem to be equivalent. The high values of ρ(*r*), ∇^2^ρ(*r*), and *E*_int_ are indicative of the importance of these interactions. In the crystal structures of complexes **2** and **3**, Pt⋯H interactions are evident. Based on ρ(r), ∇^2^ρ(r), and *E*_int_, these interactions are slightly stronger in complex **3** than those in **2** because the *E*_int_ values for the former are higher than that of the latter.

### 2.3. NBO Charges

Because we found that the H⋯O interactions are the most important intermolecular interactions in the crystal structures of the Pt complexes studied, we focus on studying these Hydrogen bonding interactions within the NBO framework. The formation of hydrogen bonds affects the charges of the atoms involved in these interactions, and the atomic charges of the hydrogen-bonded atoms are often different compared to those of atoms in the monomer or isolated molecules. Therefore, the atomic charges of the hydrogen-bonded atoms were investigated to give more insight into the electrostatic nature of the hydrogen bonds in the studied systems. The change in the charge of hydrogen-bonded atoms is a criterion used in the study of hydrogen bonding interactions. The hydrogen atom charges in the isolated complexes and cluster complexes were calculated by natural population analysis (NPA) using the NBO program implemented in G03, and the results are listed in Table 3. From this table, it is evident that the charges of the hydrogen atoms shifted to more positive values upon the formation of hydrogen bonds. The magnitude of this effect ranges from 0.0043 e to 0.0285 e for most of the hydrogen-bonded complexes. Due to these interactions, the hydrogen acceptor atoms (A) in the clusters have more negative charge compared to those in the monomers (see Table 3). However, the charges at the donor atoms do not show a clear trend for all the studied systems. The absolute NBO charge differences (ΔN_A_⋯_H_) between intermolecular hydrogen-bond-forming atom A and the hydrogen atoms were obtained by taking absolute values of the difference between the charge of atom A and the charges of the hydrogen atoms, and these values are summarized in Table 3. The calculated absolute NBO charge difference between intermolecular hydrogen bond-forming atoms (A, H) for the O⋯H-N interaction is a maximum, and accordingly, from an electrostatic point of view this is the strongest hydrogen bond type, as shown in Table 3.

A more exhaustive NBO analysis of the complex clusters and monomers was made to more accurately estimate the nature of the hydrogen bonds in the studied systems. Table 4 shows the most important donor-acceptor interactions and their second-order perturbation energies, *E*^(2)^. Estimations of the second-order perturbative charge-transfer (CT) energies listed in this table reveal the significant interactions between the lone pairs of the H-acceptor atom (A) to the D-H antibonding (σ*) orbitals. In general, the CT interactions from the second lone pair of A to the antibonding σ*- orbital are larger than that of the first lone pair. Based on results collected in Table 4, among the interactions studied, the N-H⋯O (complex **1**) and N-H⋯N (complex **3**) interactions are the strongest, and their *E*^(2)^ values were calculated to be 6.155 and 5.192 kJ/mol, respectively.

Due to the interactions between the NBOs of the D-H and an A atom or group, the occupancies and energies change significantly. The energies and occupancies of the donor and acceptor NBOs involved in these interactions compared to those of the monomer complex are listed in Table 4. Because most of the interactions involve electron donation from the hydrogen-bond acceptor atom (A) lone pair NBO to the σ*-antibonding NBO of the D-H bond, compared to the monomer, the occupancy of the former decreases but increases for the latter. In addition, their energies are affected. The data presented in Table 4 reveal the criteria for all interactions where the occupancy of the σ*(D-H) bond is increased and their energies are destabilized. In contrast, the energies of the donor NBOs lone pairs are stabilized. Notably, some of the C-H⋯O and N-H⋯O interactions not only originate from the LP(A) → σ*(D-H) interaction but from the filled π-NBO of the C=O group to the σ*(D-H) antibonding orbital.

**Figure 11:**
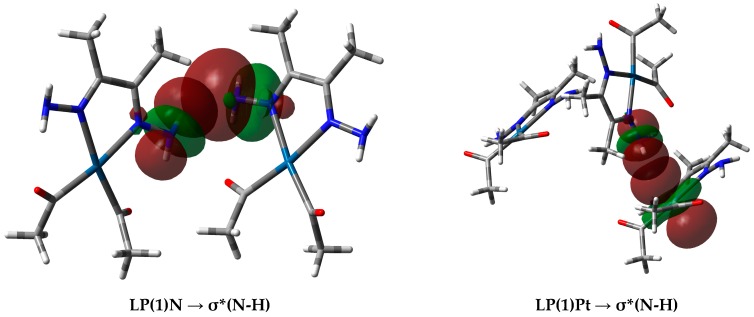
(Left) Interactions between the donor natural bond orbital (NBO) (LP(1)N) of the N-atom and the acceptor NBO (σ*N-H) of the N-H⋯N (2.273 Å) interaction, and (right) donor NBO (LP(1)Pt) to the acceptor NBO (σ*N-H) for the N-H⋯Pt (2.650 Å) interaction in complex **3**.

In the case of the Pt⋯H interactions in complex **3**, the donor NBO is from the filled lone pair NBO of the Pt atom to the antibonding σ*_N-H_ NBO. In these interactions, the NBO donor lone pair from the Pt as H-acceptor has a high contribution from the d_xy_, d_xz_, and s atomic orbitals in the NBO hybrid. A representative example of the interactions between the NBOs involved in the hydrogen-bonding interactions is shown in Figure 11. Notably, Pt⋯H interactions in **2** were not detected.

## 3. Computational Details

Hirshfeld surface analyses were carried out using Crystal explorer 3.1 [40]. Gaussian 03 [41] was used to create the wavefunction files containing the data needed for the atom in molecules (AIM) analyses. The Multiwfn program [42] was used to process the wavefunction files for topology analysis of complex clusters. In addition, natural population analyses were made using NBO 3.1 [43,44,45,46,47,48,49], which is built into Gaussian 03. The complex units and complex clusters were extracted from crystallographic information files (CIFs) obtained from the Cambridge Crystallographic Database Centre (CCDC Nos. 95819–95823 and 95825) [50]. All density functional theory calculations were performed using the B3LYP functional with 6-311G(d,p) basis sets [51,52] for nonmetal atoms and the LANL2DZ effective core potential [53,54,55,56] for Pt. The Cartesian coordinates of the clusters containing the intermolecular interactions identified from Hirshfeld analysis and used in the calculations are listed in the Supplementary Data.

## 4. Conclusions

In this work, Hirshfeld surface analysis of the crystal structures of six hydrazone-diacetyl platinum(II) complexes was conducted to determine and decompose the most important intermolecular interactions within the crystal lattices. In addition to the H⋯H contacts, C-H⋯O interactions are common in all the crystals. Following the results obtained from the Hirshfeld analysis, the AIM and NBO methods were applied to describe the nature and strength of these interactions. At the bond critical points, positive values of ∇^2^ρ(r) and electron density (ρ(r)) values in the range 0.0031–0.0156 e/a_0_^3^ indicated closed-shell H-bonding interactions. The highest ρ(r) and, hence the highest covalent character, was observed for the O2⋯H15-N3 interaction in **1**. Significant Pt··H interactions in **3** with *E*^(2)^ values in the range 3.894–4.061 kJ/mol were detected using the NBO method. We found that the d_xy_, d_xz_, and s atomic orbitals are the main contributors to the Pt-NBO hybrid orbitals.

## Figures and Tables

**Table 1:** Summary of the most important intermolecular contacts and their percentage contributions in the crystal structure of the studied complexes. The minimum contact distances are in angstroms.

Contact	1	2 (1)	2 (2)	3	4	5 (1)	5 (2)	6
C⋯H%	5.0 (2.878)	16.2 (2.632)	18.2 (2.632)	3.5 (2.876)	18.3 (2.703)	11.8 (2.699)	7.5 (2.675)	19.3 (2.669)
C⋯N%	8.6 (3.392)	1.2 (3.369)	1.1 (3.369)	2.3 (3.381)	4.6 (3.383)	3.1 (3.318)	5.2 (3.487)	3.9 (3.388)
C⋯C%	1.6 (3.509)	2.1 (3.523)	1.8 (3.523)	0.2 (3.646)	1.1 (3.427)	1.2 (3.315)	2.4 (3.446)	3.3 (3.375)
C⋯PT%	2.2 (3.474)	0.2 (3.356)	0.2 (3.356)	0.3 (3.629)	2.8 (3.430)	0.0	2.6 (3.682)	0.9 (3.484)
H⋯H%	57.0 (2.230)	51.7 (2.206)	52.4 (2.206)	54.1 (2.288)	53.8 (2.320)	46.8 (2.017)	48 (2.017)	34.9 (2.250)
H⋯O%	19.0 (2.083)	18.0 (2.323)	14.5 (2.323)	21.2 (2.385)	14.6 (2.338)	27.3 (2.324)	24.3 (2.324)	19.8 (2.278)
H⋯N%	3.5 (2.773)	6.8 (2.643)	7.7 (2.643)	12.1 (2.273)	0.4 (3.448)	4.1 (2.908)	2.2 (3.510)	1.5 (3.328)
H⋯Pt%	2.7 (3.361)	3.1 (2.901)	3.4 (2.901)	5.3 (2.650)	0.9 (3.329)	3.0 (2.960)	2.2 (2.960)	2.4 (3.057)
F⋯H%								9.5 (2.544)

**Table 2:** Topological parameters obtained from the atom in molecules (AIM) analyses of the most important contacts observed from the Hirshfeld analyses of the Pt complexes.

Contact Type	D (Å)	E_int_ (kcal/mol)	ρ(r) (e/a_0_^3^)	∇^2^ρ(r) (e/a_0_^5^)	*V*(r) (a.u.)	*G*(r) (a.u.)	*H*(r) (a.u.)	|*V*(r)|/*G*(r)
**Complex 1**								
C9⋯N3	3.404	0.9230	0.0056	0.0161	−0.0029	0.0035	0.0005	0.844
C5⋯N2	3.392	0.8683	0.0050	0.0160	−0.0028	0.0034	0.0006	0.817
C5⋯N2	3.417	0.8424	0.0049	0.0156	−0.0027	0.0033	0.0006	0.815
C9⋯N3	3.407	0.8591	0.0052	0.0148	−0.0027	0.0032	0.0005	0.850
O1⋯H8	2.360	2.0118	0.0096	0.0295	−0.0064	0.0069	0.0005	0.930
O2⋯H13	2.552	1.3478	0.0068	0.0252	−0.0043	0.0053	0.0010	0.811
O2⋯H15	2.083	3.8335	0.0156	0.0485	−0.0122	0.0122	−0.0001	1.004
O2⋯H12	2.450	1.7128	0.0084	0.0272	−0.0055	0.0061	0.0007	0.890
**Complex 2**								
C25⋯H3	2.632	1.0444	0.0071	0.0223	−0.0033	0.0045	0.0011	0.747
C31⋯H6	2.650	0.9912	0.0068	0.0209	−0.0032	0.0042	0.0010	0.753
C10⋯H32	2.697	0.7919	0.0055	0.0193	−0.0025	0.0037	0.0012	0.686
H34⋯O1	2.323	2.7102	0.0111	0.0504	−0.0086	0.0106	0.0020	0.814
H16⋯O3	2.332	2.4315	0.0104	0.0391	−0.0077	0.0088	0.0010	0.885
H12⋯O4	2.444	1.7705	0.0084	0.0328	−0.0056	0.0069	0.0013	0.815
H9⋯O2	2.486	1.7245	0.0086	0.0306	−0.0055	0.0066	0.0011	0.836
Pt2⋯H29	2.901	1.7245	0.0086	0.0306	−0.0055	0.0066	0.0011	0.836
**Complex 3**								
N4⋯H14	2.273	2.5969	0.0122	0.0382	−0.0083	0.0089	0.0006	0.929
N4⋯H14	2.273	2.6064	0.0122	0.0381	−0.0083	0.0089	0.0006	0.932
H8⋯O2	2.385	1.8904	0.0089	0.0313	−0.0060	0.0069	0.0009	0.870
H3⋯O2	2.422	1.7526	0.0085	0.0284	−0.0056	0.0063	0.0008	0.881
H2⋯O2	2.498	1.4290	0.0072	0.0250	−0.0046	0.0054	0.0008	0.843
Pt1⋯H16	2.650	2.2492	0.0114	0.0392	−0.0072	0.0085	0.0013	0.845
Pt1⋯H16	2.650	2.2517	0.0114	0.0392	−0.0072	0.0085	0.0013	0.846
**Complex 4**								
C12⋯H9	2.708	0.7821	0.0057	0.0186	−0.0025	0.0036	0.0011	0.699
H16⋯O2	2.338	1.9276	0.0089	0.0303	−0.0061	0.0069	0.0007	0.896
H16⋯O2	2.338	1.9242	0.0088	0.0305	−0.0061	0.0069	0.0007	0.891
H14⋯O2	2.467	0.9670	0.0053	0.0218	−0.0031	0.0043	0.0012	0.722
H12⋯O1	2.511	1.3375	0.0068	0.0235	−0.0043	0.0051	0.0008	0.842
H12⋯O1	2.511	1.3392	0.0068	0.0237	−0.0043	0.0051	0.0008	0.837
H10⋯O1	2.507	1.6330	0.0081	0.0303	−0.0052	0.0064	0.0012	0.814
**Complex 5**								
C17⋯H19	2.699	0.9949	0.0069	0.0240	−0.0032	0.0046	0.0014	0.692
H4⋯O3	2.396	1.6975	0.0080	0.0289	−0.0054	0.0063	0.0009	0.856
H4⋯O3	2.396	1.7129	0.0081	0.0287	−0.0055	0.0063	0.0009	0.863
H24⋯O1	2.461	1.5307	0.0075	0.0267	−0.0049	0.0058	0.0009	0.844
H25⋯O2	2.556	1.3488	0.0069	0.0270	−0.0043	0.0055	0.0012	0.777
H10⋯O5	2.324	2.2836	0.0103	0.0407	−0.0073	0.0087	0.0015	0.834
H23⋯O6	2.278	2.5966	0.0118	0.0373	−0.0083	0.0088	0.0005	0.941
H23⋯O6	2.278	2.6027	0.0118	0.0374	−0.0083	0.0088	0.0005	0.940
**Complex 6**								
H9⋯F	2.544	1.1047	0.0050	0.0260	−0.0035	0.0050	0.0015	0.703
H10⋯F	2.526	1.1840	0.0054	0.0274	−0.0038	0.0053	0.0015	0.711
C16⋯H4	2.669	0.9030	0.0063	0.0212	−0.0029	0.0041	0.0012	0.704
H8⋯O3	2.360	1.8423	0.0087	0.0283	−0.0059	0.0065	0.0006	0.907
H8⋯O3	2.360	1.8405	0.0087	0.0282	−0.0059	0.0065	0.0006	0.908
H6⋯O3	2.503	0.5211	0.0031	0.0128	−0.0017	0.0024	0.0008	0.683
H5⋯O3	2.478	1.5297	0.0076	0.0260	−0.0049	0.0057	0.0008	0.858
H5⋯O3	2.478	1.5259	0.0076	0.0259	−0.0049	0.0057	0.0008	0.857
H14⋯O2	2.278	2.5177	0.0114	0.0360	−0.0080	0.0085	0.0005	0.943
H13⋯O2	2.346	2.3741	0.0108	0.0375	−0.0076	0.0085	0.0009	0.893
H11⋯O1	2.332	2.2869	0.0104	0.0369	−0.0073	0.0083	0.0010	0.883

**Table 3:** Calculated natural charges at the D-H⋯A sites using the B3LYP functional.

Contact Type A⋯H-D	A⋯D Distance	Clusters			Monomer Complex			ΔN(H)	ΔN(D)	ΔN(A)	ΔN_A_⋯_H_
		H	D	A	H	D	A				
**Complex 1**											
O1⋯H8-C8	2.360	0.2028	–0.0799	–0.6605	0.1759	–0.1090	–0.6367	0.0269	0.0291	–0.0238	0.8633
O2⋯H13-C11	2.552	0.2086	–0.6004	–0.6842	0.1957	–0.5904	–0.6214	0.0129	–0.01	–0.0628	0.8928
O2⋯H15-N3	2.083	0.3281	–0.4735	–0.6842	0.3075	–0.4708	–0.6214	0.0206	–0.0027	–0.0628	1.0123
O2⋯H12-C11	2.450	0.2334	–0.5976	–0.6842	0.2063	–0.5904	–0.6214	0.0271	–0.0072	–0.0628	0.9176
**Complex 2**											
C25⋯H3-C2	2.632	0.1977	–0.6472	–0.2038	0.2010	–0.6467	–0.2015	–0.0033	–0.0005	–0.0023	0.4015
C31⋯H6-C4	2.650	0.2074	–0.6601	–0.1517	0.2001	–0.6522	–0.1503	0.0073	–0.0079	–0.0014	0.3591
C10⋯H32-C32	2.697	0.1847	–0.1590	0.1273	0.1781	–0.1622	0.1328	0.0066	0.0032	–0.0055	0.0574
O1⋯H34-N6	2.323	0.3398	–0.4528	–0.6923	0.3213	–0.4543	–0.6528	0.0185	0.0015	–0.0395	1.0321
O3⋯H16-N3	2.332	0.3363	–0.4663	–0.6757	0.3219	–0.4670	–0.6523	0.0144	0.0007	–0.0234	1.0120
O4⋯H12-C13	2.444	0.1911	–0.1293	–0.6149	0.1733	–0.1497	–0.6009	0.0178	0.0204	–0.014	0.8060
O2⋯H9-C9	2.486	0.1938	–0.1778	–0.6341	0.1813	–0.1954	–0.5979	0.0125	0.0176	–0.0362	0.8279
**Complex 3**											
N4⋯H14-N3	2.273	0.3205	–0.4693	–0.5312	0.3080	–0.4800	–0.4907	0.0125	0.0107	–0.0405	0.8517
N4⋯H14-N3	2.273	0.3198	–0.4702	–0.5326	0.3080	–0.4800	–0.4907	0.0118	0.0098	–0.0419	0.8524
O2⋯H8-C7	2.385	0.2367	–0.6048	–0.6671	0.2082	–0.5996	–0.6323	0.0285	–0.0052	–0.0348	0.9038
O2⋯H3-C2	2.422	0.2102	–0.6570	–0.6671	0.2015	–0.6499	–0.6323	0.0087	–0.0071	–0.0348	0.8773
O2⋯H2-C2	2.498	0.2231	–0.6637	–0.6671	0.2051	–0.6499	–0.6323	0.0180	–0.0138	–0.0348	0.8902
Pt1⋯H16-N4	2.650	0.3253	–0.4924	0.4082	0.3043	–0.4907	0.4225	0.0210	–0.0017	–0.0143	0.0829
Pt1⋯H16-N4	2.650	0.3199	–0.4881	0.4136	0.3043	–0.4907	0.4225	0.0156	0.0026	–0.0089	0.0937
**Complex 4**											
C12⋯H9-C8	2.708	0.1792	–0.1077	–0.2004	0.1749	–0.1088	0.1817	0.0043	0.0011	–0.3821	0.3796
O2⋯H16-C16	2.338	0.1910	–0.2396	–0.6429	0.1742	–0.2277	–0.5938	0.0168	–0.0119	–0.0491	0.8339
O2⋯H16-C16	2.338	0.1925	–0.2367	–0.6461	0.1742	–0.2277	–0.5938	0.0183	–0.009	–0.0523	0.8386
O2⋯H14-C14	2.467	0.1817	–0.1455	–0.6429	0.1712	–0.1799	–0.5938	0.0105	0.0344	–0.0491	0.8246
O1⋯H12-C12	2.511	0.1933	–0.1915	–0.6827	0.1817	–0.1856	–0.6594	0.0116	–0.0059	–0.0233	0.8760
O1⋯H12-C12	2.511	0.1959	–0.2010	–0.6797	0.1817	–0.1856	–0.6594	0.0142	–0.0154	–0.0203	0.8756
O1⋯H10-C9	2.507	0.1895	–0.1959	–0.6827	0.1820	–0.1986	–0.6594	0.0075	0.0027	–0.0233	0.8722
**Complex 5**											
C17⋯H19-C16	2.699	0.2109	–0.6558	0.0684	0.2040	–0.6470	0.0832	0.0069	–0.0088	–0.0148	0.1425
O3⋯H4-C4	2.396	0.2193	–0.6544	–0.6382	0.1982	–0.6486	–0.6272	0.02112	–0.0058	–0.01103	0.8576
O3⋯H4-C4	2.396	0.2193	–0.6544	–0.6382	0.1982	–0.6486	–0.6272	0.02112	–0.0058	–0.01103	0.8576
O1⋯H24-C20	2.461	0.1886	–0.0996	–0.6982	0.1777	–0.1017	–0.6724	0.0109	0.0021	–0.0258	0.8868
O2⋯H25-C21	2.556	0.1986	–0.1619	–0.6303	0.1825	–0.1879	–0.5975	0.0161	0.026	–0.0328	0.8289
O5⋯H10-C9	2.324	0.2069	–0.1683	–0.6463	0.1853	–0.1912	–0.5927	0.0216	0.0229	–0.0536	0.8532
O6⋯H23-C19	2.278	0.2010	–0.1787	–0.6460	0.1877	–0.1816	–0.6144	0.0133	0.0029	–0.0316	0.8470
O6⋯H23-C19	2.278	0.2034	–0.1742	–0.6452	0.1877	–0.1816	–0.6144	0.0157	0.0074	–0.0308	0.8486
**Complex 6**											
H9⋯F-C15	2.544	0.1890	–0.1830	–0.3625	0.1827	–0.1849	–0.3451	0.0063	0.0019	–0.0174	0.5515
H10⋯F-C15	2.526	0.1824	–0.0970	–0.3625	0.1774	–0.1078	–0.3451	0.0050	0.0108	–0.0174	0.5449
C16⋯H4-C4	2.669	0.1960	–0.6500	–0.2518	0.1910	–0.6513	–0.2459	0.0050	0.0013	–0.0059	0.4478
O3⋯H8-C7	2.360	0.2067	–0.1633	–0.6540	0.1862	–0.1730	–0.6268	0.0205	0.0097	–0.0272	0.8607
O3⋯H8-C7	2.360	0.2028	–0.1665	–0.6450	0.1862	–0.1730	–0.6268	0.0166	0.0065	–0.0182	0.8478
O3⋯H6-C4	2.503	0.2195	–0.6698	–0.6336	0.2052	–0.6513	–0.6268	0.0143	–0.0185	–0.0068	0.8531
O3⋯H5-C4	2.478	0.2030	–0.6528	–0.6301	0.1938	–0.6513	–0.6268	0.0092	–0.0015	–0.0033	0.8331
O3⋯H5-C4	2.478	0.2055	–0.6528	–0.6331	0.1938	–0.6513	–0.6268	0.0117	–0.0015	–0.0063	0.8386
O2⋯H14-C16	2.278	0.2137	–0.2478	–0.6088	0.1917	–0.2459	–0.5928	0.0220	–0.0019	–0.016	0.8225
O2⋯H13-C14	2.346	0.2167	–0.2310	–0.6240	0.1930	–0.2377	–0.5928	0.0237	0.0067	–0.0312	0.8407
O1⋯H11-C10	2.332	0.1852	0.1362	–0.6918	0.1715	0.1394	–0.6786	0.0137	–0.0032	–0.0132	0.8770

**Table 4:** Natural bond orbitals (NBOs) involved in the intermolecular interactions, their occupancies, energies (a.u.), and the second-order perturbation energies, *E*^(2)^, (KJ/mol).

Contact type A⋯H-D	A⋯D Dist.	(NBO) i	(Occupancy) i	*E*_i_	(NBO) j	(Occupancy) j	*E*_j_	*E*^(2)^
**Complex 1**								
O1⋯H8-C8	2.360	LP(1)O1	1.97137 (1.97169)	–0.66824 (–0.64828)	σ*(C8-H8)	0.01397 (0.01265)	0.71211 (0.66653)	0.628
		LP(2)O1	1.86311 (1.85818)	–0.26356 (–0.24078)	σ*(C8-H8)	0.01397 (0.01265)	0.71211 (0.66653)	2.261
O2⋯H13-C11	2.552	π(C3-O2)	1.99011 (0.53040)	–0.36890 (–0.33956)	σ*(C11-H13)	0.00788 (0.00670)	0.55924 (0.53040)	1.005
O2⋯H15-N3	2.083	LP(1)O2	1.97123 (0.73470)	–0.67080 (0.73470)	σ*(N3-H15)	0.01347 (0.73470)	0.78719 (0.73470)	2.386
		LP(2)O2	1.87487 (1.86076)	–0.26693 (–0.23237)	σ*(N3-H15)	0.01347 (0.00912)	0.78719 (0.73470)	6.155
		π(C3=O2)	1.99011 (1.98993)	–0.36890 (–0.33956)	σ*(N3-H15)	0.01347 (0.00912)	0.78719 (0.73470)	0.544
O2⋯H12-C11	2.450	LP(1)O2	1.97123 (1.97351)	–0.67080 (–0.64215)	σ*(C11-H12)	0.01168 (0.00886)	0.57514 (0.52857)	0.628
		LP(2)O2	1.87487 (1.86076)	–0.26693 (–0.23237)	σ*(C11-H12)	0.01168 (0.00886)	0.57514 (0.52857)	2.680
**Complex 2**								
C25⋯H3-C2	2.632	π(C25-C26)	1.63140 (1.63245)	–0.27948 (–0.29051)	σ*(C2-H3)	0.00867 (0.00810)	0.59404 (0.59837)	1.214
C31⋯H6-C4	2.650	π(C31-C32)	1.64661 (1.65943)	–0.26358 (–0.27596)	σ*(C4-H6)	0.00826 (0.00734)	0.59477 (0.58464)	1.382
C10⋯H32-C32	2.697	σ(C32-H32)	1.97739 (1.97819)	–0.57490 (–0.58925)	π*(C10-N2)	0.43750 (0.43608)	–0.03118 (–0.04048)	0.586
		π(C10-N2)	1.76913 (1.75890)	–0.33656 (–0.34197)	σ*(C32-H32)	0.01374 (0.01350)	0.69918 (0.67576)	0.586
O1⋯H34-N6	2.323	LP(1)O1	1.97191 (1.97185)	–0.66711 (–0.65175)	σ*(N6-H34)	0.01218 (0.01182)	0.80595 (0.77963)	0.209
		LP(2)O1	1.88227 (1.87315)	–0.25176 (–0.23319)	σ*(N6-H34)	0.01218 (0.01182)	0.80595 (0.77963)	0.879
O3⋯H16-N3	2.332	π(C17-O3)	1.98910 (1.98983)	–0.35705 (–0.33828)	σ*(N3-H17)	0.01256 (0.01108)	0.74628 (0.71888)	0.795
		LP(2)O3	1.87408 (1.86995)	–0.25616 (–0.23664)	σ*(N3-H17 )	0.01256 (0.01108)	0.74628 (0.71888)	0.963
O4⋯H12-C13	2.444	LP(2)O4	1.85563 (1.85073)	–0.24363 (–0.23150)	σ*(C13-H12)	0.01286 (0.01281)	0.72157 (0.68308)	0.461
O2⋯H9-C9	2.486	LP(2)O2	1.84626 (1.84547)	–0.23524 (–0.23060)	σ*(C9-H9)	0.01214 (0.01129)	0.65796 (0.66930)	0.712
**Complex 3**								
N4⋯H14-N3	2.273	LP(1)N4	1.85986 (1.84172)	–0.36581 (–0.34055)	σ*(N3-H14)	0.01164 (0.00884)	0.81339 (0.78729)	5.192
N4⋯H14-N3	2.273	LP (1)N4	1.86168 (1.84172)	–0.35759 (–0.34055)	σ*(N3-H14)	0.01161 (0.00884)	0.82344 (0.78729)	5.192
O2⋯H8-C7	2.385	LP(1)O2	1.97169 (1.97261)	–0.66099 (–0.64978)	σ*(C7-H8)	0.01186 (0.01044)	0.58379 (0.52851)	1.675
		LP(2)O2	1.86505 (1.85902)	–0.25512 (–0.24097)	σ*(C7-H8)	0.01186 (0.01044)	0.58379 (0.52851)	0.963
O2⋯H3-C2	2.422	LP(1)O2	1.97169 (1.97261)	–0.66099 (–0.64978)	σ*(C2-H3)	0.01035 (0.00768)	0.62219 (0.60008)	0.293
		LP(2)O2	1.86505 (1.85902)	–0.25512 (–0.24097)	σ*(C2-H3)	0.01035 (0.00768)	0.62219 (0.60008)	1.005
		π(C3-O2)	1.99010 (1.99023)	–0.36459 (–0.35580)	σ*(C2-H3)	0.01035 (0.00768)	0.62219 (0.60008)	1.424
O2⋯H2-C2	2.498	LP(1)O2	1.97169 (1.97261)	–0.66099 (–0.64978)	σ*(C2-H2)	0.00979 (0.00743)	0.61830 (0.58366)	1.005
		LP(2)O2	1.86505 (1.85902)	–0.25512 (–0.24097)	σ*(C2-H2)	0.00979 (0.00743)	0.61830 (0.58366)	1.633
Pt1⋯H16-N4	2.650	LP(1)Pt1	1.87880 (1.99158)	–0.26130 (–0.24303)	σ*(N4-H16)	0.01058 (0.00839)	0.77742 (0.74610)	4.061
Pt1⋯H16-N4	2.650	LP(1)Pt1	1.91600 (1.91561)	–0.46068 (–0.45392)	σ*(N4-H16)	0.01057 (0.00839)	0.76746 (0.74610)	3.894
**Complex 4**								
C12⋯H9-C8	2.708	σ(C8-H9)	1.97880 (1.97943)	–0.59280 (–0.60808)	π*(C12-C13)	0.01734 (0.30861)	0.57893 (0.02913)	0.461
		π(C12-C13)	1.97514 (1.97564)	–0.71226 (–0.69807)	σ*(C8-H9)	0.01350 (0.01312)	0.69110 (0.66991)	0.419
O2⋯H16–C16	2.338	π(C3-O2)	1.98923 (1.98983)	–0.38610 (–0.35347)	σ*(C16-H16)	0.01424 (0.01304)	0.69995 (0.67898)	1.549
		LP(1)O2	1.97068 (1.97264)	–0.67192 (–0.63955)	σ*(C16-H16)	0.01424 (0.01304)	0.69995 (0.67898)	1.089
O2⋯H16-C16	2.338	π(C3-O2)	1.98947 (1.98983)	–0.37977 (–0.35347)	σ*(C16-H16)	0.01423 (0.01304)	0.69933 (0.67898)	1.591
		LP(1)O2	1.97110 (1.97264)	–0.66751 (–0.63955)	σ*(C16-H16)	0.01423 (0.01304)	0.69933 (0.67898)	1.089
O2⋯H14-C14	2.467	LP(1)O2	1.97068 (1.97264)	–0.67192 (–0.63955)	σ*(C14-H14)	0.01283 (0.01256)	0.72291 (0.69606)	0.419
O1⋯H12-C12	2.511	LP(1)O1	1.96440 (1.99026)	–0.69115 (–0.35247)	σ*(C12-H12)	0.01253 (0.01158)	0.70138 (0.68580)	0.544
		LP(2)O1	1.86902 (1.86203)	–0.28550 (–0.25502)	σ*(C12-H12)	0.01253 (0.01158)	0.70138 (0.68580)	0.502
O1⋯H12-C12	2.511	LP(1)O1	1.96527 (1.99026)	–0.68160 (–0.35247)	σ*(C12-H12)	0.01253 (0.01158)	0.70098 (0.68580)	0.544
		LP(2)O1	1.86794 (1.86203)	–0.27517 (–0.25502)	σ*(C12-H12)	0.01253 (0.01158)	0.70098 (0.68580)	0.544
O1⋯H10-C9	2.507	LP(2)O1	1.86902 (1.86203)	–0.28550 (–0.25502)	σ*(C9-H10)	0.01279 (0.01193)	0.68180 (0.66621)	0.419
**Complex 5**								
C17⋯H19-C16	2.699	σ(C16-H19)	1.96707 (1.96868)	–0.54074 (–0.53534)	π*(C17-N4)	0.01548 (0.25607)	0.54744 (−0.03872)	1.130
		π(C17-N4)	1.98824 (1.92080)	–0.91673 (–0.37610)	σ*(C16-H19)	0.00922 (0.00877)	0.57426 (0.57142)	0.377
O3⋯H4-C4	2.396	π(C11-O3)	1.98337 (1.99523)	(–0.39427) (–1.06665)	σ*(C4-H4)	0.00963 (0.00835)	0.59373 (0.56896)	0.7534
		LP(1)O3	1.97713 (1.97866)	(–0.70347) (–0.69621)	σ*(C4-H4)	0.00963 (0.00835)	0.59373 (0.56896)	1.6324
O3⋯H4-C4	2.396	π(C11-O3)	1.98337 (1.99523)	(–0.39427) (–1.06665)	σ*(C4-H4)	0.00963 (0.00835)	0.59373 (0.56896)	0.7534
		LP(1)O3	1.97713 (1.97866)	–0.70347 (–0.69621)	σ*(C4-H4)	0.00963 (0.00835)	0.59373 (0.56896)	1.6324
O1⋯H24-C20	2.461	LP(1)O1	1.96648 (1.96689)	–0.68368 (–0.67330)	σ*(C20-H24)	0.01276 (0.01209)	0.70137 (0.66118)	0.7540
		LP(1)O1	1.87419 (1.86803)	–0.26999 (–0.25705)	σ*(C20-H24)	0.01276 (0.01209)	0.70137 (0.66118)	0.2930
O2⋯H25-C21	2.556	LP(2)O2	1.97269 (1.86803)	–0.65652 (–0.25705)	σ*(C21-H25)	0.01146 (0.01137)	0.70641 (0.65909)	0.209
O5⋯H10-C9	2.324	LP(1)O5	1.97273 (1.97354)	–0.66258 (–0.65175)	σ*(C9-H10)	0.01165 (0.01184)	0.69823 (0.65601)	0.628
O6⋯H23-C19	2.278	LP(1)O6	1.97702 (1.97845)	–0.69553 (–0.69126)	σ*(C19-H23)	0.01490 (0.01292)	0.68884 (0.64597)	0.586
		LP(2)O6	1.87205 (1.86984)	–0.27847 (–0.27097)	σ*(C19-H23)	0.01490 (0.01292)	0.68884 (0.64597)	2.721
O6⋯H23-C19	2.278	LP(1)O6	1.97712 (1.97845)	–0.69886 (–0.69126)	σ*(C19-H23)	0.01473 (0.01292)	0.68329 (0.64597)	0.586
		LP(2)O6	1.87194 (1.86984)	–0.28146 (–0.27097)	σ*(C19-H23)	0.01473 (0.01292)	0.68329 (0.64597)	2.680
**Complex 6**								
C16⋯H4-C4	2.669	σ(C4-H4)	1.98252 (1.98376)	–0.53852 (–0.53308)	π*(C16-C17)	0.29616 (0.30335)	0.02878 (0.01810)	0.544
		π(C16-C17)	1.66193 (1.66922)	–0.26125 (–0.27086)	σ*(C4-H4)	0.01050 (0.01030)	0.58775 (0.58738)	1.005
O3⋯H8-C7	2.360	LP(1)O3	1.97802 (1.97901)	–0.71166 (–0.70195)	σ*(C7-H8)	0.01316 (0.01180)	0.67217 (0.64842)	1.256
		LP(2)O3	1.87851 (1.87585)	–0.28994 (–0.27736)	σ*(C7-H8)	0.01316 (0.01180)	0.67217 (0.64842)	1.758
O3⋯H8-C7	2.360	LP(1)O3	1.97816 (1.97901)	–0.71673 (–0.70195)	σ*(C7-H8)	0.01316 (0.01180)	0.67815 (0.64842)	1.256
		LP(2)O3	1.87761 (1.87585)	–0.29446 (–0.27736)	σ*(C7-H8)	0.01316 (0.01180)	0.67815 (0.64842)	1.675
O3⋯H6-C4	2.503	LP(1)O3	1.97816 (1.97901)	–0.69908 (–0.70195)	σ*(C4-H6)	0.01029 (0.00859)	0.58979 (0.57290)	0.670
		LP(2)O3	1.87657 (1.87585)	–0.27608 (–0.27736)	σ*(C4-H6)	0.01029 (0.00859)	0.58979 (0.5729)	0.502
O3⋯H5-C4	2.478	π(C11-O3)	1.97576 (1.97564)	–0.39637 (–0.39003)	σ*(C4-H5)	0.00951 (0.0082)	0.58267 (0.56896)	1.256
		LP(1)O3	1.97818 (1.97901)	–0.70613 (–0.70195)	σ*(C4-H5)	0.00951 (0.0082)	0.58267 (0.56896)	0.025
O3⋯H5-C4	2.478	π(C3-O3)	1.97554 (1.97564)	–0.39514 (–0.39003)	σ*(C4-H5)	0.00972 (0.0082)	0.57705 (0.56896)	1.298
		LP(1)O3	1.97828 (1.97901)	–0.70568 (–0.70195)	σ*(C4-H5)	0.00972 (0.00820)	0.57705 (0.56896)	0.251
O2⋯H14-C16	2.278	LP(1)O2	1.97197 (1.97301)	–0.66209 (–0.64902)	σ*(C16-H14)	0.01430 (0.01217)	0.70710 (0.67024)	0.921
		LP(2)O2	1.85410 (1.85107)	–0.25874 (–0.24082)	σ*(C16-H14)	0.01430 (0.01217)	0.70710 (0.67024)	2.763
O2⋯H13-C14	2.346	LP(1)O2	1.97245 (1.97301)	–0.65870 (–0.64902)	σ*(C14-H13)	0.01325 (0.01153)	0.70642 (0.67422)	0.322
		LP(2)O2	1.85944 (1.85107)	–0.25185 (–0.24082)	σ*(C14-H13)	0.01325 (0.01153)	0.70642 (0.67422)	2.010
O1⋯H11-C10	2.332	π(C1-O1)	1.98826 (1.98947)	–0.37137 (–0.35014)	σ*(C10-H11)	0.01643 (0.01559)	0.67607 (0.65302)	0.502
		LP(1)O1	1.96970 (1.97111)	–0.69047 (–0.67364)	σ*(C10-H11)	0.01643 (0.01559)	0.67607 (0.65302)	0.335

Values in parentheses relate to the monomers (non-interacting units).

## Supplementary Materials

Supplementary materials can be accessed at: http://www.mdpi.com/1420-3049/21/12/1669/s1.
