# Supplementary material for: Decomposition of Intermolecular Interactions in the Crystal Structure of Some Diacetyl Platinum(II) Complexes: Combined Hirshfeld, AIM, and NBO Analyses
Source: Molecules. 2016 Dec 6;21(12):1669. doi: 10.3390/molecules21121669 (PMC6273681; doi:10.3390/molecules21121669)
Supplement: Supplementary file 1 [file molecules-21-01669-s001.pdf]

Supplementary Materials: Decomposition of Intermolecular Interactions in the Crystal Structure of Some Diacetyl Platinum(II) Complexes: A Combined Hirshfeld, AIM, and NBO Analyses

Saied M. Soliman and Assem Barakat

Complex 1

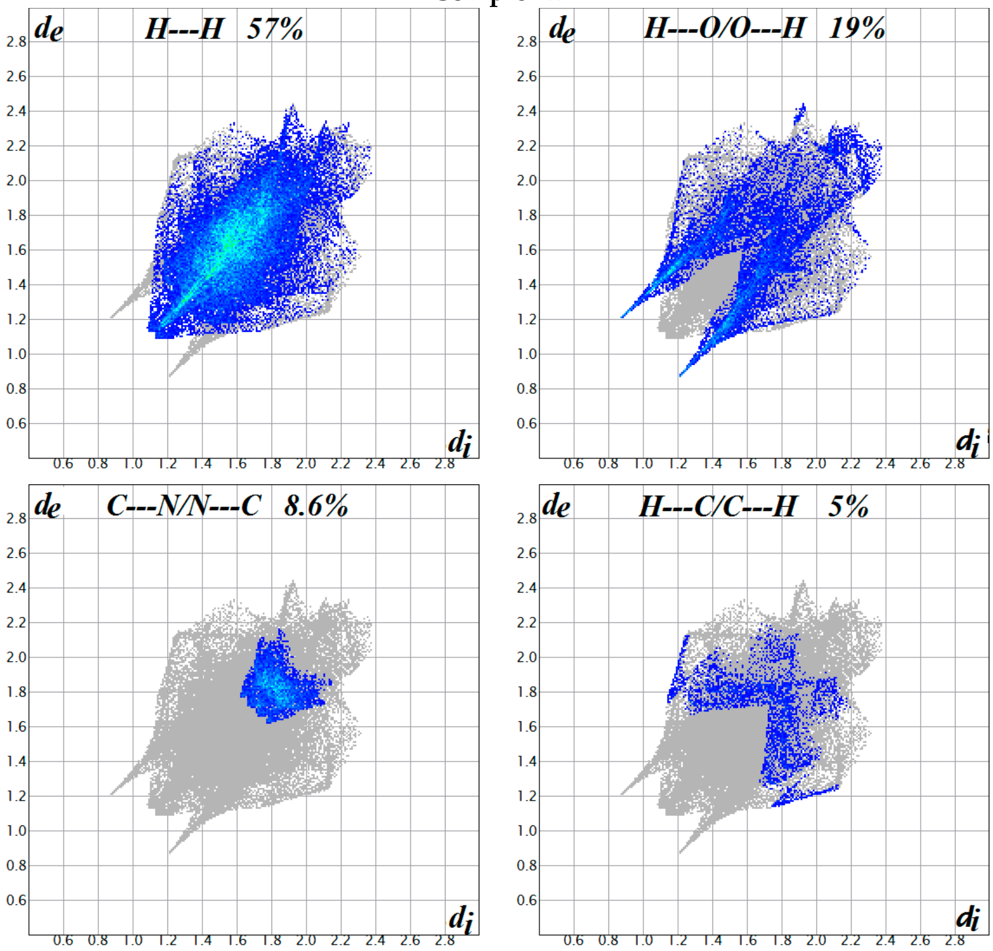

Complex 2 (Unit 1)

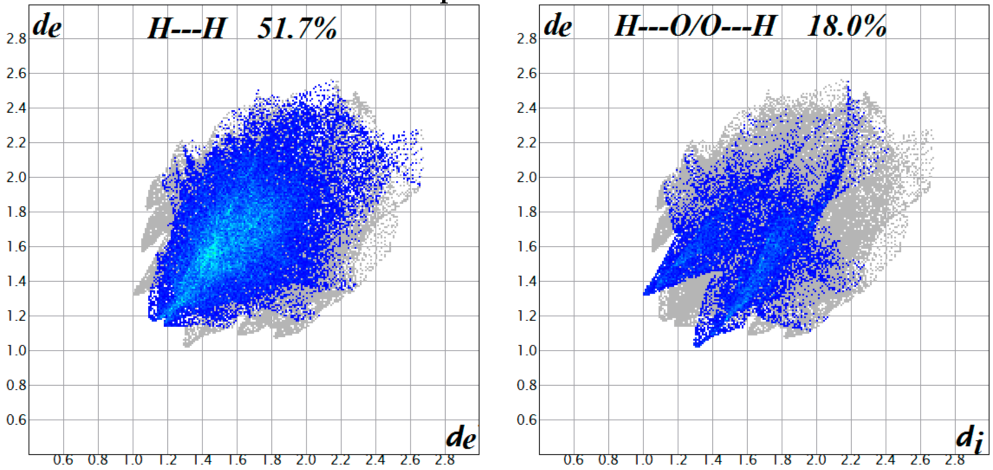

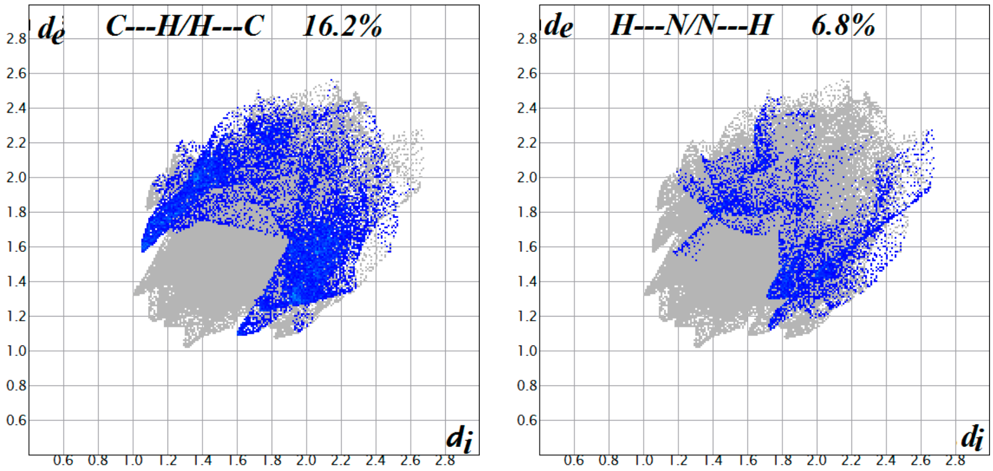

Complex 2 (Unit 2)

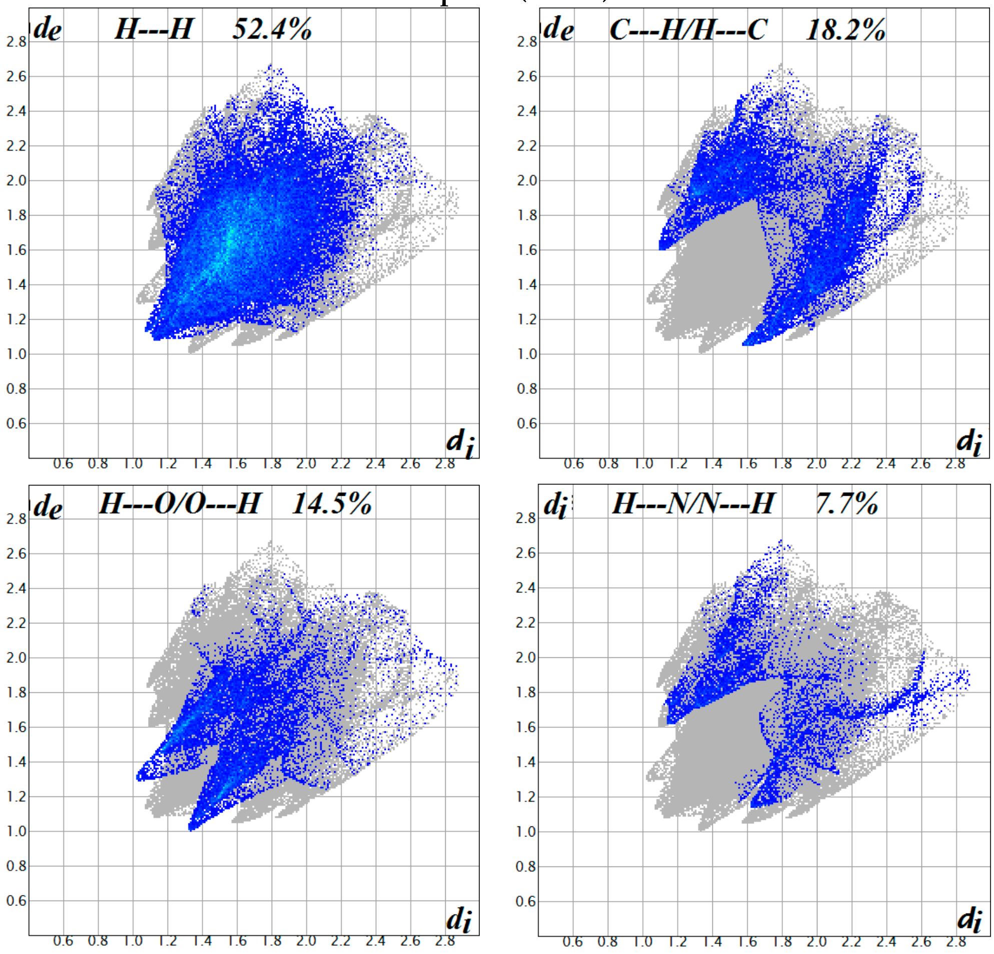

Complex3

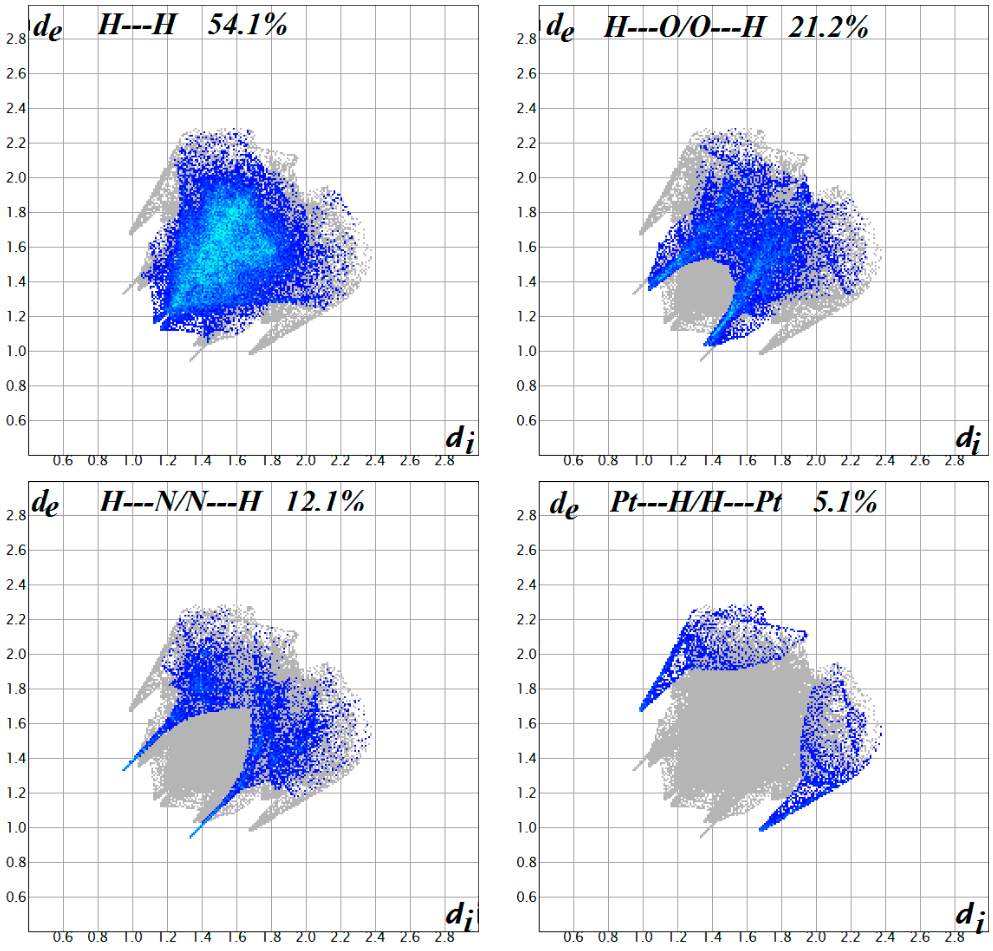

Complex 4

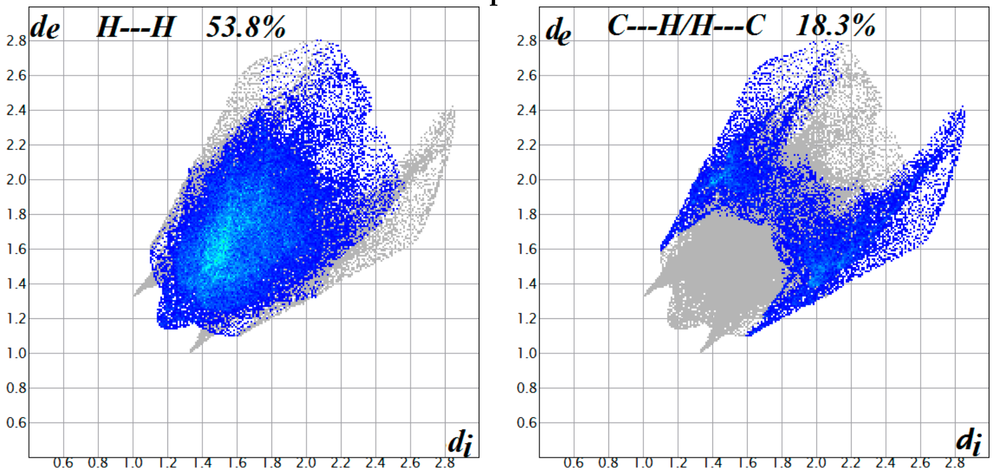

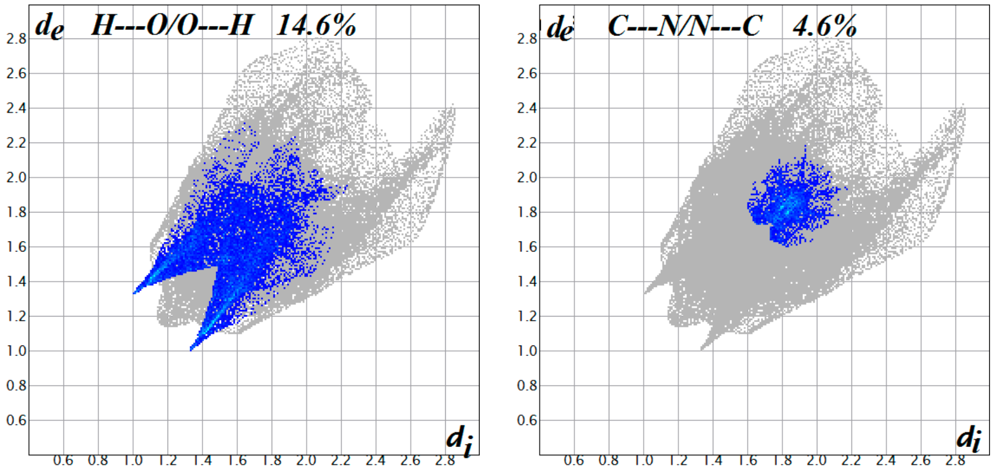

Complex 5 (Unit 1)

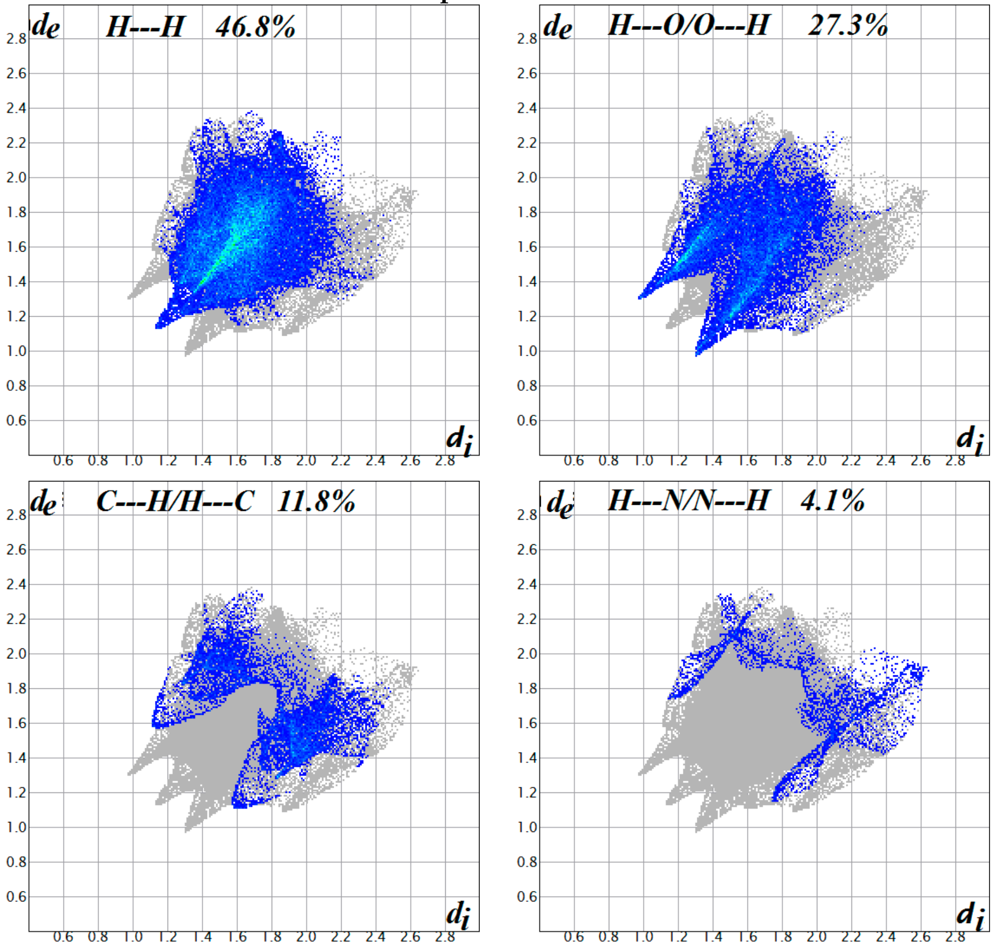

Complex 5 (Unit 2)

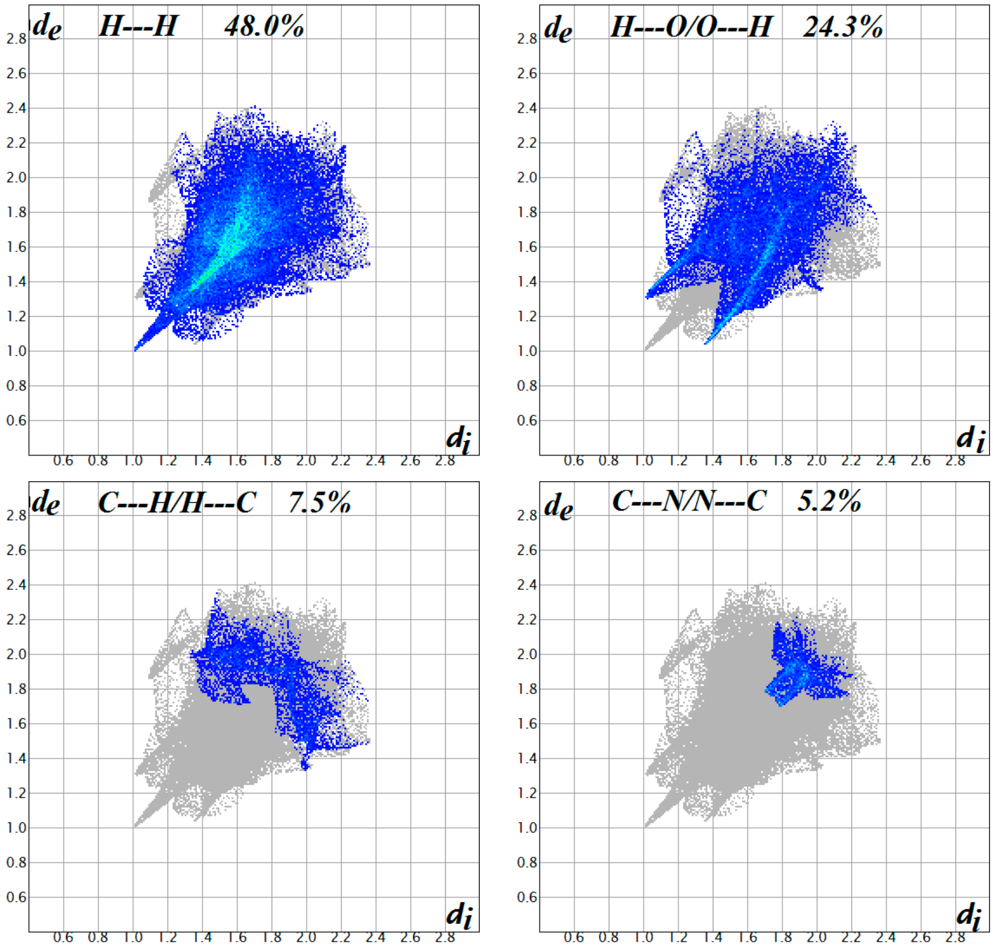
de H---H 48.0%
di
de H---O/O---H 24.3%
di
de C---H/H---C 7.5%
di
de C---N/N---C 5.2%
di

Complex 6

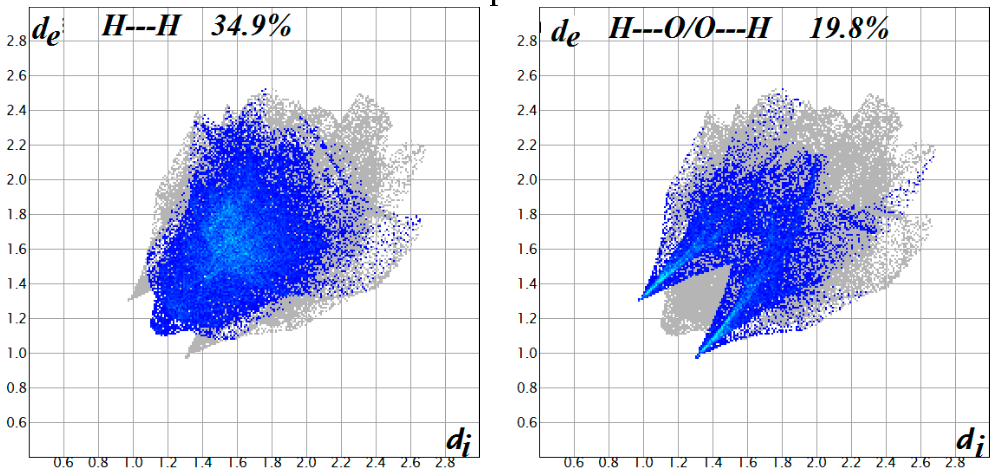
de H---H 34.9%
di
de H---O/O---H 19.8%
di

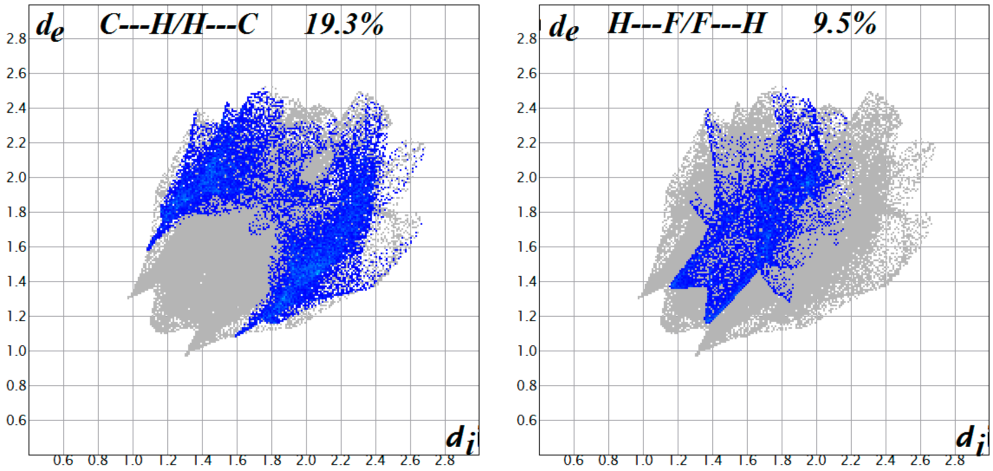

Figure S1. The decomposed fingerprint maps of the most significant intermolecular interactions.

Table S1. The rest of the intermolecular contacts obtained from the Hirsheld surfaces analysis of the studied complexes, the minimum contact distances are in Å.

Contact	1	2 (1)	2 (2)	3	4	5 (1)	5 (2)	6
C---O%	0.1	0.7	0.7	0.4	1.5	1.4	3	1.2
N---N%	0.1	0	0	0.6	1.8	0.4	1.3	1
N---O%	0	0	0	0	0	0.8	0.8	0
O---O%	0.2	0	0	0	0	0.1	0.1	0.4
Pt---N%	0	0	0	0	0.1	0	0	0
Pt---O%	0	0	0	0	0	0	0.3	0
Pt---Pt%	0	0	0	0	0	0	0	0
F---Pt%								0.2
F---C%								0.1
F---F%								0
F---O%								0.9
F---N%								0.8

Front **Back**

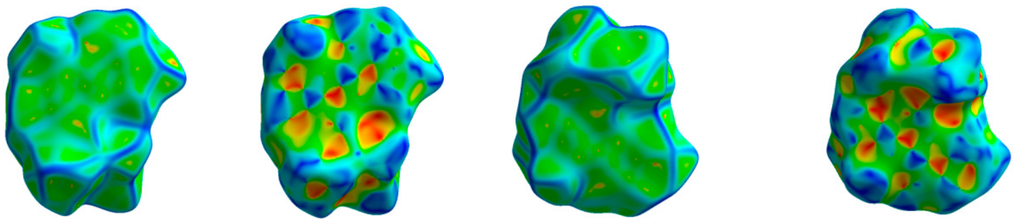

1

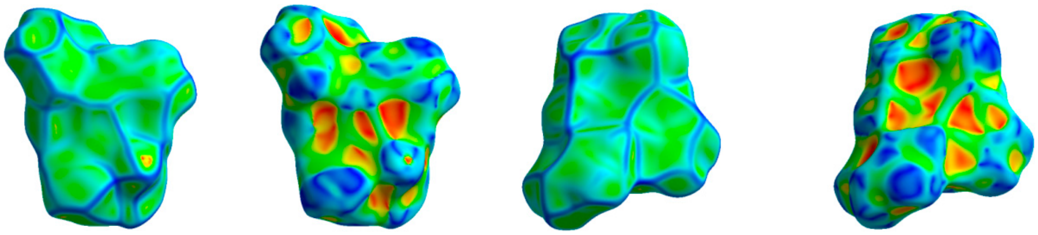

2 (Unit 1)

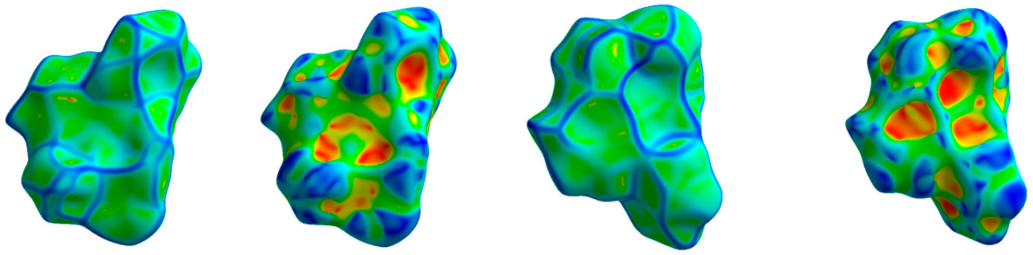

2 (Unit 2)

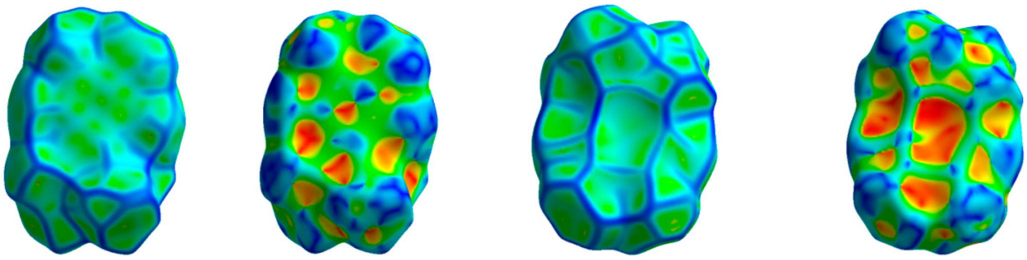

3

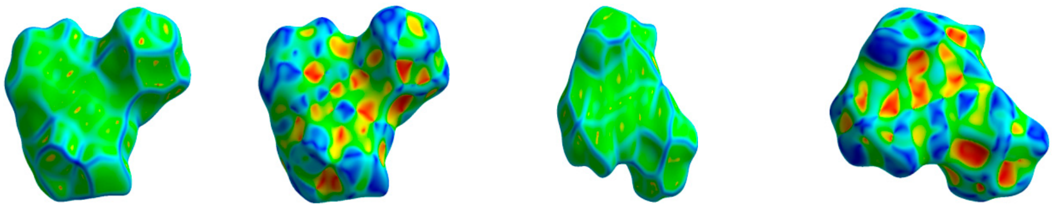

4

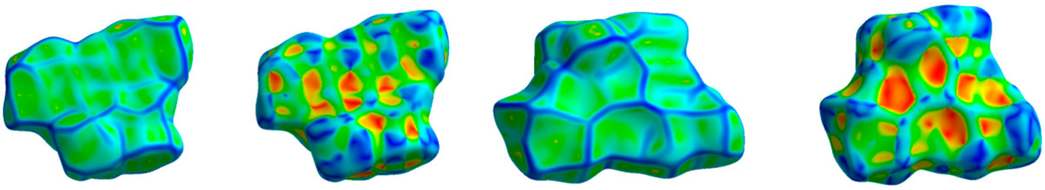

5 (Unit 1)

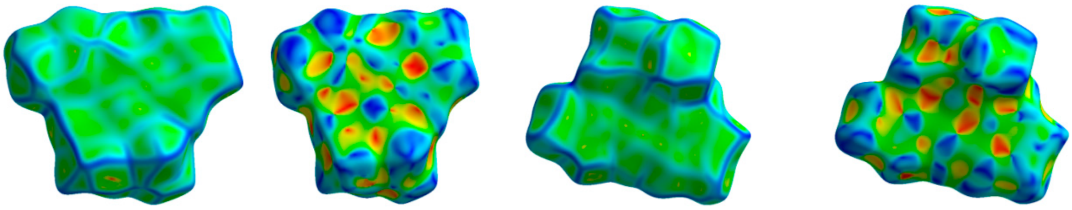

5 (Unit 2)

5

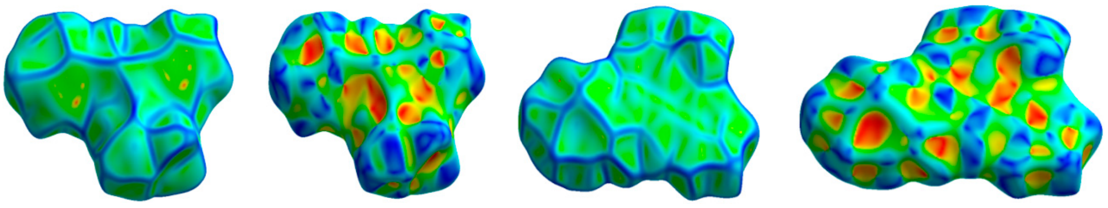

6

Figure S2. The curvedness and shape index plots of the studied complexes.

Clusters Information

Complex 1			
C---N interactions			
C	−4.282708000	−1.160034000	−0.350596000
C	−4.755144000	−1.030645000	−1.773503000
H	−5.734701000	−1.038629000	−1.794150000
H	−4.411009000	−1.779745000	−2.301922000
H	−4.427722000	−0.187081000	−2.151025000
C	−3.975078000	1.638095000	−0.276799000
C	−5.437078000	1.744359000	0.104416000
H	−5.901148000	2.317177000	−0.541381000
H	−5.513226000	2.133632000	1.000382000
H	−5.841982000	0.851579000	0.100152000
C	−0.570891000	−1.022517000	1.817487000
C	−0.280737000	0.411246000	1.735862000
C	0.881307000	0.987260000	2.235475000
H	1.534077000	0.443581000	2.660331000
C	1.087624000	2.348868000	2.114935000
H	1.872832000	2.751587000	2.467799000
C	0.139122000	3.114555000	1.476403000
H	0.259781000	4.049960000	1.371292000
C	−0.998804000	2.486018000	0.989156000
H	−1.650478000	3.013459000	0.543585000
C	0.388016000	−1.970221000	2.495882000
H	0.093591000	−2.893261000	2.349612000
H	0.406593000	−1.781862000	3.457444000
H	1.285258000	−1.849570000	2.121366000
N	−1.673007000	−1.395099000	1.271714000
N	−1.220110000	1.169252000	1.118211000
N	−2.068893000	−2.707373000	1.268803000
H	−2.911549000	−2.757494000	1.174930000
H	−1.744196000	−3.108626000	1.950146000
O	−4.688009000	−2.100719000	0.311456000
O	−3.517916000	2.563868000	−0.941196000
Pt	−2.894773000	0.096870000	0.364474000
C	4.440315000	1.110703000	0.055535000
C	5.847363000	0.688780000	−0.271269000
H	6.419168000	0.824390000	0.512739000
H	6.184491000	1.224645000	−1.018263000
H	5.854585000	−0.259527000	−0.520245000
C	3.825769000	−1.591361000	0.556324000
C	4.327950000	−1.397202000	1.972015000
H	5.037803000	−2.046951000	2.157671000
H	3.589228000	−1.531421000	2.601588000
H	4.681806000	−0.488444000	2.071845000
C	0.558970000	1.026248000	−1.795169000
C	0.266295000	−0.406726000	−1.708825000
C	−0.875582000	−0.989051000	−2.246193000
H	−1.516378000	−0.449121000	−2.693466000
C	−1.079379000	−2.351449000	−2.130371000
H	−1.865217000	−2.753970000	−2.482056000
C	−0.127726000	−3.118122000	−1.497738000

H	−0.237670000	−4.056881000	−1.412685000
C	0.997597000	−2.485640000	−0.986896000
H	1.656833000	−3.015450000	−0.555482000
C	−0.406239000	1.975925000	−2.461766000
H	0.004775000	2.862472000	−2.533768000
H	−1.224562000	2.037887000	−1.926103000
H	−0.625367000	1.643023000	−3.356764000
N	1.676841000	1.393899000	−1.278892000
N	1.196845000	−1.161970000	−1.074655000
N	2.081550000	2.703411000	−1.292500000
H	2.641239000	2.842101000	−0.668875000
H	1.398260000	3.216904000	−1.302510000
O	4.258885000	2.235035000	0.491919000
O	3.981809000	−2.709066000	0.072731000
Pt	2.902830000	−0.099392000	−0.379556000
O---H interactions			
C	3.910494000	−5.053480000	−1.376552000
C	4.897885000	−5.128516000	−0.243402000
H	5.807391000	−5.171261000	−0.605332000
H	4.721256000	−5.929099000	0.292019000
H	4.808496000	−4.331861000	0.321114000
C	3.826184000	−2.261509000	−1.019627000
C	5.036362000	−2.153861000	−1.924030000
H	5.756191000	−1.686780000	−1.450514000
H	4.797001000	−1.652486000	−2.731134000
H	5.338477000	−3.052189000	−2.174543000
C	−0.322798000	−4.509509000	−1.906096000
C	−0.442598000	−3.090315000	−1.562489000
C	−1.656431000	−2.415638000	−1.506196000
H	−2.463597000	−2.876815000	−1.701126000
C	−1.690050000	−1.075812000	−1.167360000
H	−2.514970000	−0.604535000	−1.141825000
C	−0.511108000	−0.432574000	−0.867365000
H	−0.506298000	0.484233000	−0.622355000
C	0.671539000	−1.156864000	−0.931350000
H	1.484262000	−0.714471000	−0.718805000
C	−1.541276000	−5.327474000	−2.258388000
H	−1.289320000	−6.270894000	−2.339379000
H	−1.909174000	−5.013388000	−3.110695000
H	−2.215371000	−5.227943000	−1.554433000
N	0.869923000	−4.986769000	−1.874713000
N	0.719362000	−2.451633000	−1.278902000
N	1.129161000	−6.300221000	−2.168712000
H	1.937022000	−6.389885000	−2.415117000
H	0.538108000	−6.597210000	−2.710112000
O	3.955412000	−5.911369000	−2.242279000
O	3.733178000	−1.417005000	−0.133266000
Pt	2.461835000	−3.668403000	−1.355580000
C	8.354509000	2.295039000	−0.019009000
C	9.560409000	2.710038000	−0.817874000
H	10.374584000	2.453501000	−0.336908000
H	9.543056000	2.266654000	−1.690753000

H	9.549258000	3.682153000	−0.945132000
C	8.198444000	4.926088000	0.972368000
C	9.184221000	4.530485000	2.052093000
H	9.961512000	5.126436000	2.017526000
H	8.754347000	4.604609000	2.929477000
H	9.474400000	3.605393000	1.907104000
C	4.081583000	2.750124000	−0.198106000
C	3.961193000	4.167996000	0.150712000
C	2.752075000	4.853246000	0.165317000
H	1.947709000	4.398348000	−0.054367000
C	2.719046000	6.194394000	0.498942000
H	1.893978000	6.665340000	0.525780000
C	3.898724000	6.839285000	0.792423000
H	3.906040000	7.761710000	1.015286000
C	5.078424000	6.108386000	0.754493000
H	5.892916000	6.554745000	0.951406000
C	2.861631000	1.928854000	−0.537370000
H	3.140845000	1.046565000	−0.859375000
H	2.306757000	1.823619000	0.263545000
H	2.346077000	2.383934000	−1.235202000
N	5.277987000	2.281125000	−0.199291000
N	5.121090000	4.802052000	0.452539000
N	5.539288000	0.972298000	−0.511531000
H	6.280992000	0.734269000	−0.172989000
H	4.864398000	0.487293000	−0.311651000
O	8.262253000	1.129517000	0.328148000
O	8.248802000	6.092105000	0.591129000
Pt	6.870886000	3.601704000	0.311113000
C	−4.123931000	2.172921000	−0.452338000
C	−3.136540000	2.097885000	0.680813000
H	−2.227035000	2.055140000	0.318883000
H	−3.313169000	1.297302000	1.216234000
H	−3.225930000	2.894540000	1.245328000
C	−4.208241000	4.964892000	−0.095413000
C	−2.998063000	5.072540000	−0.999815000
H	−2.278235000	5.539621000	−0.526299000
H	−3.237424000	5.573915000	−1.806919000
H	−2.695948000	4.174213000	−1.250329000
C	−8.357223000	2.716893000	−0.981881000
C	−8.477024000	4.136086000	−0.638274000
C	−9.690857000	4.810763000	−0.581981000
H	−10.498022000	4.349586000	−0.776912000
C	−9.724475000	6.150590000	−0.243145000
H	−10.549395000	6.621866000	−0.217610000
C	−8.545533000	6.793828000	0.056849000
H	−8.540723000	7.710635000	0.301859000
C	−7.362887000	6.069537000	−0.007135000
H	−6.550163000	6.511930000	0.205410000
C	−9.575701000	1.898928000	−1.334173000
H	−9.323745000	0.955508000	−1.415165000
H	−9.943599000	2.213014000	−2.186480000
H	−10.249796000	1.998459000	−0.630218000

N	−7.164503000	2.239632000	−0.950498000
N	−7.315063000	4.774769000	−0.354687000
N	−6.905264000	0.926180000	−1.244497000
H	−6.097403000	0.836516000	−1.490902000
H	−7.496317000	0.629192000	−1.785897000
O	−4.079013000	1.315033000	−1.318064000
O	−4.301247000	5.809396000	0.790949000
Pt	−5.572591000	3.557999000	−0.431366000
C	−4.094774000	−2.464291000	1.882910000
C	−5.300674000	−2.879290000	2.681774000
H	−6.114849000	−2.622753000	2.200808000
H	−5.283321000	−2.435905000	3.554653000
H	−5.289522000	−3.851405000	2.809032000
C	−3.938709000	−5.095340000	0.891532000
C	−4.924485000	−4.699737000	−0.188193000
H	−5.701776000	−5.295688000	−0.153625000
H	−4.494612000	−4.773861000	−1.065576000
H	−5.214665000	−3.774645000	−0.043204000
C	0.178152000	−2.919376000	2.062006000
C	0.298542000	−4.337248000	1.713189000
C	1.507660000	−5.022498000	1.698584000
H	2.312026000	−4.567599000	1.918267000
C	1.540689000	−6.363646000	1.364958000
H	2.365757000	−6.834592000	1.338120000
C	0.361011000	−7.008536000	1.071477000
H	0.353695000	−7.930962000	0.848615000
C	−0.818689000	−6.277637000	1.109408000
H	−1.633181000	−6.723997000	0.912495000
C	1.398104000	−2.098106000	2.401271000
H	1.118890000	−1.215817000	2.723275000
H	1.952979000	−1.992871000	1.600355000
H	1.913658000	−2.553185000	3.099102000
N	−1.018252000	−2.450377000	2.063192000
N	−0.861355000	−4.971303000	1.411362000
N	−1.279553000	−1.141550000	2.375431000
H	−2.021257000	−0.903521000	2.036890000
H	−0.604662000	−0.656545000	2.175552000
O	−4.002518000	−1.298769000	1.535752000
O	−3.989067000	−6.261357000	1.272771000
Pt	−2.611151000	−3.770956000	1.552788000

Complex 2			
C---H interactions			
C	0.050936000	1.131496000	5.426208000
C	0.658363000	2.507494000	5.411918000
H	1.580678000	2.460740000	5.740862000
H	0.135408000	3.101951000	5.990794000
H	0.655921000	2.857120000	4.496353000
C	2.121772000	0.492859000	3.647316000
C	3.225457000	0.207682000	4.651358000
H	2.828268000	−0.030764000	5.514880000
H	3.784045000	1.006202000	4.757526000
H	3.777229000	−0.535402000	4.328322000
C	−1.762981000	−2.252241000	3.463760000
C	−0.711888000	−2.548295000	2.481484000
C	−0.835716000	−3.506189000	1.500764000
H	−1.637795000	−4.013274000	1.441395000
C	0.190104000	−3.740982000	0.598920000
H	0.108899000	−4.402134000	−0.079425000
C	1.353137000	−2.967795000	0.722667000
H	2.094898000	−3.113134000	0.146831000
C	1.402648000	−1.994659000	1.692092000
H	2.185326000	−1.457771000	1.754487000
C	−2.954763000	−3.145140000	3.583210000
C	−4.237113000	−2.626288000	3.428034000
H	−4.361142000	−1.701406000	3.252833000
C	−5.330790000	−3.468112000	3.529652000
H	−6.204764000	−3.121230000	3.403449000
C	−5.159222000	−4.796930000	3.811240000
H	−5.916929000	−5.365292000	3.887001000
C	−3.902891000	−5.310060000	3.984074000
H	−3.794080000	−6.231621000	4.190088000
C	−2.785531000	−4.495191000	3.861603000
H	−1.914696000	−4.858049000	3.965790000
N	−1.563619000	−1.217181000	4.212739000
N	0.397200000	−1.761413000	2.561965000
N	−2.479567000	−0.826939000	5.135457000
H	−2.982105000	−1.459025000	5.445084000
H	−2.191295000	−0.289316000	5.741233000
O	−0.682507000	0.892201000	6.400536000
O	2.407410000	1.090089000	2.626456000
Pt	0.320909000	−0.225265000	4.015050000
C	−1.666610000	−5.218349000	−3.000221000
C	−2.489212000	−6.250204000	−3.723454000
H	−1.900350000	−6.820003000	−4.261335000
H	−3.134191000	−5.801080000	−4.309209000
H	−2.970284000	−6.801500000	−3.070373000
C	−3.927405000	−5.046540000	−1.320366000
C	−3.817940000	−5.717291000	0.019926000
H	−4.294239000	−6.574312000	−0.003419000
H	−4.217061000	−5.141821000	0.705795000
H	−2.873988000	−5.872571000	0.232597000
C	−1.204899000	−1.117776000	−1.691654000

C	−2.462558000	−0.987706000	−0.945699000
C	−2.896023000	0.210763000	−0.418942000
H	−2.376065000	0.998064000	−0.535106000
C	−4.084544000	0.265846000	0.277236000
H	−4.381464000	1.085129000	0.656994000
C	−4.833921000	−0.879179000	0.416327000
H	−5.656958000	−0.865285000	0.892577000
C	−4.368224000	−2.042579000	−0.147200000
H	−4.885895000	−2.831919000	−0.047074000
C	−0.234066000	0.007924000	−1.781248000
C	0.035284000	0.613873000	−3.012152000
H	−0.401270000	0.307511000	−3.797826000
C	0.941271000	1.663997000	−3.087423000
H	1.125436000	2.068148000	−3.926807000
C	1.575922000	2.124911000	−1.953258000
H	2.182561000	2.853779000	−2.006129000
C	1.318220000	1.514873000	−0.730562000
H	1.758732000	1.819869000	0.054137000
C	0.425387000	0.469004000	−0.656095000
H	0.258298000	0.056603000	0.184112000
N	−0.985913000	−2.290060000	−2.204527000
N	−3.209881000	−2.117693000	−0.835438000
N	0.136102000	−2.571746000	−2.885720000
H	0.359917000	−3.410948000	−2.951443000
H	0.763359000	−2.006308000	−2.933358000
O	−0.460601000	−5.220370000	−3.254727000
O	−4.949247000	−5.207795000	−1.948950000
Pt	−2.467668000	−3.811947000	−1.854108000
C	6.808868000	−2.146366000	−2.522102000
C	6.201440000	−3.522363000	−2.507812000
H	5.279126000	−3.475610000	−2.836756000
H	6.724394000	−4.116821000	−3.086688000
H	6.203883000	−3.871990000	−1.592247000
C	4.738031000	−1.507729000	−0.743210000
C	3.634346000	−1.222552000	−1.747251000
H	4.031535000	−0.984105000	−2.610774000
H	3.075759000	−2.021072000	−1.853420000
H	3.082575000	−0.479468000	−1.424216000
C	8.622785000	1.237371000	−0.559654000
C	7.571691000	1.533425000	0.422622000
C	7.695520000	2.491319000	1.403342000
H	8.497599000	2.998404000	1.462711000
C	6.669699000	2.726112000	2.305186000
H	6.750905000	3.387264000	2.983532000
C	5.506665000	1.952926000	2.181439000
H	4.764906000	2.098264000	2.757275000
C	5.457155000	0.979789000	1.212014000
H	4.674478000	0.442901000	1.149619000
C	9.814567000	2.130270000	−0.679104000
C	11.096917000	1.611419000	−0.523928000
H	11.220945000	0.686537000	−0.348727000
C	12.190593000	2.453242000	−0.625546000

H	13.064567000	2.106360000	−0.499343000
C	12.019025000	3.782060000	−0.907134000
H	12.776732000	4.350422000	−0.982895000
C	10.762695000	4.295190000	−1.079968000
H	10.653883000	5.216751000	−1.285982000
C	9.645335000	3.480322000	−0.957497000
H	8.774499000	3.843179000	−1.061684000
N	8.423422000	0.202311000	−1.308633000
N	6.462603000	0.746543000	0.342141000
N	9.339370000	−0.187931000	−2.231351000
H	9.841907000	0.444155000	−2.540977000
H	9.051099000	−0.725554000	−2.837127000
O	7.542309000	−1.907070000	−3.496430000
O	4.452394000	−2.104959000	0.277650000
Pt	6.538894000	−0.789605000	−1.110944000
C	−5.374163000	1.261897000	−2.949268000
C	−5.981590000	−0.114100000	−2.934978000
H	−6.903905000	−0.067347000	−3.263922000
H	−5.458636000	−0.708557000	−3.513855000
H	−5.979148000	−0.463726000	−2.019412000
C	−7.444999000	1.900534000	−1.170376000
C	−8.548684000	2.185711000	−2.174418000
H	−8.151495000	2.424158000	−3.037940000
H	−9.107272000	1.387191000	−2.280586000
H	−9.100456000	2.928796000	−1.851383000
C	−3.560246000	4.645634000	−0.986820000
C	−4.611339000	4.941689000	−0.004544000
C	−4.487511000	5.899583000	0.976176000
H	−3.685432000	6.406668000	1.035544000
C	−5.513332000	6.134376000	1.878020000
H	−5.432126000	6.795527000	2.556365000
C	−6.676365000	5.361189000	1.754272000
H	−7.418125000	5.506527000	2.330108000
C	−6.725875000	4.388052000	0.784847000
H	−7.508553000	3.851164000	0.722453000
C	−2.368464000	5.538534000	−1.106270000
C	−1.086114000	5.019682000	−0.951095000
H	−0.962085000	4.094800000	−0.775894000
C	0.007563000	5.861505000	−1.052713000
H	0.881537000	5.514623000	−0.926509000
C	−0.164005000	7.190323000	−1.334300000
H	0.593701000	7.758685000	−1.410062000
C	−1.420336000	7.703453000	−1.507135000
H	−1.529148000	8.625015000	−1.713148000
C	−2.537696000	6.888585000	−1.384664000
H	−3.408532000	7.251442000	−1.488850000
N	−3.759608000	3.610575000	−1.735800000
N	−5.720427000	4.154806000	−0.085026000
N	−2.843660000	3.220333000	−2.658517000
H	−2.341123000	3.852419000	−2.968144000
H	−3.131932000	2.682710000	−3.264294000
O	−4.640721000	1.501193000	−3.923596000

O	−7.730637000	1.303305000	−0.149516000
Pt	−5.644137000	2.618658000	−1.538110000
O---H interactions			
C	−4.996881000	2.206752000	−0.242557000
C	−5.226032000	1.914193000	1.214989000
H	−5.283276000	0.944667000	1.348395000
H	−6.064012000	2.333322000	1.504955000
H	−4.481498000	2.272569000	1.741999000
C	−2.646372000	0.718665000	0.105843000
C	−3.212782000	−0.678182000	−0.082690000
H	−4.042259000	−0.627291000	−0.601982000
H	−3.399397000	−1.075458000	0.793822000
H	−2.561085000	−1.233486000	−0.560086000
C	−2.669054000	3.852441000	−3.477383000
C	−1.410779000	3.127573000	−3.256877000
C	−0.260537000	3.365246000	−3.974624000
H	−0.262562000	4.030625000	−4.653783000
C	0.899423000	2.650441000	−3.721042000
H	1.695743000	2.810979000	−4.214976000
C	0.856665000	1.681134000	−2.708919000
H	1.617927000	1.145003000	−2.519283000
C	−0.306317000	1.519024000	−1.994479000
H	−0.320567000	0.876507000	−1.293269000
C	−2.823771000	4.748653000	−4.662604000
C	−3.153898000	6.089689000	−4.488404000
H	−3.289525000	6.439730000	−3.616302000
C	−3.282037000	6.910288000	−5.595345000
H	−3.486208000	7.829386000	−5.478477000
C	−3.116443000	6.404127000	−6.856640000
H	−3.214445000	6.973843000	−7.610771000
C	−2.810852000	5.082818000	−7.036353000
H	−2.708491000	4.737861000	−7.916173000
C	−2.649707000	4.241543000	−5.943745000
H	−2.421395000	3.329188000	−6.071638000
N	−3.608334000	3.632525000	−2.616306000
N	−1.431149000	2.224664000	−2.237035000
N	−4.804527000	4.268876000	−2.698996000
H	−5.035719000	4.515528000	−3.495023000
H	−5.445922000	3.914804000	−2.249163000
O	−6.013476000	2.535006000	−0.877615000
O	−1.802987000	0.890633000	0.965817000
Pt	−3.234705000	2.136559000	−1.134108000
C	4.996881000	−2.206752000	0.242557000
C	5.226032000	−1.914193000	−1.214989000
H	5.283276000	−0.944667000	−1.348395000
H	6.064012000	−2.333322000	−1.504955000
H	4.481498000	−2.272569000	−1.741999000
C	2.646372000	−0.718665000	−0.105843000
C	3.212782000	0.678182000	0.082690000
H	4.042259000	0.627291000	0.601982000
H	3.399397000	1.075458000	−0.793822000
H	2.561085000	1.233486000	0.560086000

C	2.669054000	−3.852441000	3.477383000
C	1.410779000	−3.127573000	3.256877000
C	0.260537000	−3.365246000	3.974624000
H	0.262562000	−4.030625000	4.653783000
C	−0.899423000	−2.650441000	3.721042000
H	−1.695743000	−2.810979000	4.214976000
C	−0.856665000	−1.681134000	2.708919000
H	−1.617927000	−1.145003000	2.519283000
C	0.306317000	−1.519024000	1.994479000
H	0.320567000	−0.876507000	1.293269000
C	2.823771000	−4.748653000	4.662604000
C	3.153898000	−6.089689000	4.488404000
H	3.289525000	−6.439730000	3.616302000
C	3.282037000	−6.910288000	5.595345000
H	3.486208000	−7.829386000	5.478477000
C	3.116443000	−6.404127000	6.856640000
H	3.214445000	−6.973843000	7.610771000
C	2.810852000	−5.082818000	7.036353000
H	2.708491000	−4.737861000	7.916173000
C	2.649707000	−4.241543000	5.943745000
H	2.421395000	−3.329188000	6.071638000
N	3.608334000	−3.632525000	2.616306000
N	1.431149000	−2.224664000	2.237035000
N	4.804527000	−4.268876000	2.698996000
H	5.035719000	−4.515528000	3.495023000
H	5.445922000	−3.914804000	2.249163000
O	6.013476000	−2.535006000	0.877615000
O	1.802987000	−0.890633000	−0.965817000
Pt	3.234705000	−2.136559000	1.134108000
O---H interactions			
C	−1.750695000	0.635617000	1.877275000
C	−0.778861000	−0.510799000	1.953005000
H	−1.260273000	−1.333462000	2.181505000
H	−0.336277000	−0.621016000	1.085371000
H	−0.105669000	−0.324043000	2.641341000
C	0.563785000	2.212603000	2.222115000
C	0.774709000	2.802016000	3.588268000
H	1.446248000	2.275343000	4.071528000
H	1.088496000	3.726414000	3.499362000
H	−0.070348000	2.790150000	4.084376000
C	−3.085407000	4.217575000	−0.154556000
C	−1.827258000	4.972493000	−0.107306000
C	−1.655041000	6.175408000	−0.759249000
H	−2.366268000	6.547270000	−1.268727000
C	−0.449908000	6.838820000	−0.670811000
H	−0.329446000	7.676245000	−1.104180000
C	0.574635000	6.274590000	0.053034000
H	1.414652000	6.714044000	0.128210000
C	0.358551000	5.063321000	0.664646000
H	1.066337000	4.677695000	1.166043000
C	−4.312562000	4.789632000	−0.774704000
C	−4.859350000	4.212277000	−1.924719000

H	−4.448515000	3.450138000	−2.314696000
C	−6.003526000	4.752262000	−2.497880000
H	−6.374577000	4.352728000	−3.275385000
C	−6.605583000	5.864006000	−1.947330000
H	−7.378470000	6.240095000	−2.351383000
C	−6.069865000	6.431534000	−0.796477000
H	−6.484266000	7.191737000	−0.405085000
C	−4.939495000	5.892595000	−0.223461000
H	−4.582690000	6.286126000	0.565060000
N	−3.056499000	3.059181000	0.430705000
N	−0.811643000	4.395623000	0.587868000
N	−4.138890000	2.267953000	0.499092000
H	−4.149604000	1.664126000	1.126746000
H	−4.889803000	2.542098000	0.222656000
O	−2.937592000	0.342854000	2.034770000
O	1.517960000	1.710234000	1.672151000
Pt	−1.209591000	2.474915000	1.369905000
C	−7.326337000	0.117329000	−0.648198000
C	−8.258979000	0.935893000	−1.498299000
H	−9.097857000	1.084050000	−1.013131000
H	−7.842656000	1.800404000	−1.701411000
H	−8.445222000	0.458176000	−2.333527000
C	−9.351297000	−1.818204000	−0.766226000
C	−10.230378000	−1.319817000	0.367958000
H	−9.693667000	−0.778985000	0.984180000
H	−10.957194000	−0.773651000	0.001395000
H	−10.608862000	−2.085593000	0.848909000
C	−4.944089000	−3.399511000	0.092008000
C	−5.987688000	−4.424765000	−0.038638000
C	−5.734568000	−5.777104000	0.003963000
H	−4.842029000	−6.081939000	0.124136000
C	−6.760501000	−6.699652000	−0.126915000
H	−6.590050000	−7.634498000	−0.097301000
C	−8.060132000	−6.204608000	−0.304868000
H	−8.799772000	−6.797690000	−0.368937000
C	−8.246883000	−4.845237000	−0.385270000
H	−9.127954000	−4.517512000	−0.530308000
C	−3.559952000	−3.782438000	0.503607000
C	−2.474948000	−3.467411000	−0.309556000
H	−2.608204000	−3.001369000	−1.125970000
C	−1.199972000	−3.839252000	0.079404000
H	−0.461041000	−3.644503000	−0.482858000
C	−0.998869000	−4.485434000	1.269569000
H	−0.118717000	−4.729462000	1.531631000
C	−2.058363000	−4.781204000	2.082915000
H	−1.907937000	−5.220314000	2.912440000
C	−3.351597000	−4.444428000	1.706577000
H	−4.085897000	−4.665981000	2.265680000
N	−5.314706000	−2.181417000	−0.133722000
N	−7.243653000	−3.949789000	−0.268168000
N	−4.431330000	−1.152481000	−0.073775000
H	−3.754785000	−1.278546000	0.450001000

H	−4.777659000	−0.368621000	−0.005047000
O	−6.453977000	0.769394000	−0.049606000
O	−9.888510000	−2.241599000	−1.772490000
Pt	−7.398427000	−1.850693000	−0.484657000
C	9.255767000	−3.473148000	−0.054414000
C	10.188409000	−4.291712000	0.795688000
H	11.027287000	−4.439870000	0.310520000
H	9.772085000	−5.156223000	0.998800000
H	10.374652000	−3.813996000	1.630915000
C	11.280727000	−1.537615000	0.063615000
C	12.159808000	−2.036002000	−1.070569000
H	11.623098000	−2.576834000	−1.686791000
H	12.886624000	−2.582168000	−0.704006000
H	12.538292000	−1.270226000	−1.551520000
C	6.873519000	0.043692000	−0.794619000
C	7.917117000	1.068946000	−0.663973000
C	7.663998000	2.421286000	−0.706575000
H	6.771460000	2.726120000	−0.826747000
C	8.689930000	3.343832000	−0.575696000
H	8.519480000	4.278679000	−0.605310000
C	9.989562000	2.848789000	−0.397743000
H	10.729202000	3.441871000	−0.333675000
C	10.176313000	1.489418000	−0.317341000
H	11.057384000	1.161692000	−0.172304000
C	5.489382000	0.426618000	−1.206219000
C	4.404379000	0.111592000	−0.393056000
H	4.537634000	−0.354450000	0.423359000
C	3.129403000	0.483433000	−0.782016000
H	2.390471000	0.288684000	−0.219754000
C	2.928300000	1.129615000	−1.972180000
H	2.048147000	1.373642000	−2.234243000
C	3.987794000	1.425384000	−2.785527000
H	3.837367000	1.864494000	−3.615052000
C	5.281028000	1.088609000	−2.409189000
H	6.015327000	1.310161000	−2.968291000
N	7.244136000	−1.174402000	−0.568889000
N	9.173084000	0.593970000	−0.434444000
N	6.360760000	−2.203338000	−0.628837000
H	5.684215000	−2.077274000	−1.152613000
H	6.707090000	−2.987198000	−0.697565000
O	8.383407000	−4.125214000	−0.653006000
O	11.817940000	−1.114221000	1.069878000
Pt	9.327857000	−1.505126000	−0.217955000
Pt---H interactions			
C	−1.123508000	−0.558533000	5.270098000
C	−1.352659000	−0.851092000	6.727644000
H	−1.409903000	−1.820618000	6.861050000
H	−2.190639000	−0.431963000	7.017610000
H	−0.608125000	−0.492716000	7.254654000
C	1.227001000	−2.046620000	5.618498000
C	0.660591000	−3.443467000	5.429965000
H	−0.168886000	−3.392576000	4.910673000

H	0.473976000	−3.840743000	6.306477000
H	1.312288000	−3.998771000	4.952569000
C	1.204319000	1.087156000	2.035272000
C	2.462594000	0.362288000	2.255778000
C	3.612836000	0.599961000	1.538031000
H	3.610811000	1.265340000	0.858872000
C	4.772796000	−0.114844000	1.791613000
H	5.569116000	0.045694000	1.297679000
C	4.730038000	−1.084151000	2.803736000
H	5.491300000	−1.620282000	2.993372000
C	3.567056000	−1.246261000	3.518176000
H	3.552806000	−1.888778000	4.219386000
C	1.049602000	1.983368000	0.850051000
C	0.719475000	3.324404000	1.024251000
H	0.583848000	3.674445000	1.896353000
C	0.591336000	4.145003000	−0.082690000
H	0.387165000	5.064101000	0.034178000
C	0.756930000	3.638842000	−1.343985000
H	0.658928000	4.208558000	−2.098116000
C	1.062521000	2.317533000	−1.523698000
H	1.164882000	1.972576000	−2.403518000
C	1.223666000	1.476258000	−0.431090000
H	1.451978000	0.563903000	−0.558983000
N	0.265039000	0.867240000	2.896349000
N	2.442224000	−0.540621000	3.275620000
N	−0.931154000	1.503591000	2.813659000
H	−1.162346000	1.750243000	2.017632000
H	−1.572549000	1.149519000	3.263492000
O	−2.140103000	−0.230279000	4.635040000
O	2.070386000	−1.874652000	6.478472000
Pt	0.638668000	−0.628726000	4.378547000
C	1.123508000	0.558533000	−5.270098000
C	1.352659000	0.851092000	−6.727644000
H	1.409903000	1.820618000	−6.861050000
H	2.190639000	0.431963000	−7.017610000
H	0.608125000	0.492716000	−7.254654000
C	−1.227001000	2.046620000	−5.618498000
C	−0.660591000	3.443467000	−5.429965000
H	0.168886000	3.392576000	−4.910673000
H	−0.473976000	3.840743000	−6.306477000
H	−1.312288000	3.998771000	−4.952569000
C	−1.204319000	−1.087156000	−2.035272000
C	−2.462594000	−0.362288000	−2.255778000
C	−3.612836000	−0.599961000	−1.538031000
H	−3.610811000	−1.265340000	−0.858872000
C	−4.772796000	0.114844000	−1.791613000
H	−5.569116000	−0.045694000	−1.297679000
C	−4.730038000	1.084151000	−2.803736000
H	−5.491300000	1.620282000	−2.993372000
C	−3.567056000	1.246261000	−3.518176000
H	−3.552806000	1.888778000	−4.219386000
C	−1.049602000	−1.983368000	−0.850051000

C	−0.719475000	−3.324404000	−1.024251000
H	−0.583848000	−3.674445000	−1.896353000
C	−0.591336000	−4.145003000	0.082690000
H	−0.387165000	−5.064101000	−0.034178000
C	−0.756930000	−3.638842000	1.343985000
H	−0.658928000	−4.208558000	2.098116000
C	−1.062521000	−2.317533000	1.523698000
H	−1.164882000	−1.972576000	2.403518000
C	−1.223666000	−1.476258000	0.431090000
H	−1.451978000	−0.563903000	0.558983000
N	−0.265039000	−0.867240000	−2.896349000
N	−2.442224000	0.540621000	−3.275620000
N	0.931154000	−1.503591000	−2.813659000
H	1.162346000	−1.750243000	−2.017632000
H	1.572549000	−1.149519000	−3.263492000
O	2.140103000	0.230279000	−4.635040000
O	−2.070386000	1.874652000	−6.478472000
Pt	−0.638668000	0.628726000	−4.378547000

**Complex 3**			
N---H interactions			
C	0.632766000	−2.041346000	0.188730000
C	0.942966000	−3.328193000	0.907703000
H	0.444077000	−3.359065000	1.750965000
H	1.903292000	−3.374747000	1.094396000
H	0.681467000	−4.087876000	0.345912000
C	−2.226538000	−2.675892000	1.123414000
H	−1.949555000	−2.645102000	0.183817000
H	−3.202496000	−2.627693000	1.178431000
H	−1.917452000	−3.514453000	1.527370000
C	−1.622423000	−1.510441000	1.865001000
C	0.070577000	2.452963000	1.637692000
C	1.235051000	2.172138000	0.775441000
C	−0.255775000	3.834728000	2.104104000
H	−1.219284000	3.987204000	2.023552000
H	0.226641000	4.487653000	1.554649000
H	0.013649000	3.934379000	3.041257000
C	2.182047000	3.251173000	0.346543000
H	3.099765000	2.905973000	0.365863000
H	2.107740000	4.014099000	0.957106000
H	1.960858000	3.538598000	−0.563590000
N	−1.775434000	1.584868000	2.735834000
H	−1.653628000	2.209296000	3.307777000
H	−1.916040000	0.826186000	3.144794000
N	2.460923000	0.505542000	−0.282437000
H	2.279707000	−0.227798000	−0.707611000
H	2.829137000	1.116881000	−0.725627000
N	1.376293000	0.926287000	0.431585000
N	−0.643210000	1.436324000	1.965925000
O	0.936259000	−1.958892000	−0.991359000
O	−2.155095000	−1.182377000	2.919198000
Pt	−0.097663000	−0.444467000	1.132631000

C	−5.940463000	−1.922686000	0.266319000
C	−5.645001000	−3.146848000	−0.560273000
H	−6.159876000	−3.109719000	−1.393615000
H	−4.688440000	−3.177389000	−0.768372000
H	−5.896343000	−3.949730000	−0.056968000
C	−8.817532000	−2.481669000	−0.662074000
H	−8.522688000	−2.526853000	0.271487000
H	−9.794323000	−2.429927000	−0.694416000
H	−8.516841000	−3.284525000	−1.138419000
C	−8.226746000	−1.259527000	−1.318217000
C	−6.526665000	2.673759000	−0.802982000
C	−5.346203000	2.324933000	0.011371000
C	−6.860770000	4.088503000	−1.149673000
H	−7.822453000	4.233212000	−1.038709000
H	−6.367478000	4.695163000	−0.558434000
H	−6.609161000	4.263923000	−2.080637000
C	−4.390381000	3.366426000	0.508131000
H	−3.473465000	3.024632000	0.443452000
H	−4.475710000	4.176230000	−0.037121000
H	−4.593982000	3.579036000	1.442595000
N	−8.393918000	1.896015000	−1.932469000
H	−8.282541000	2.564800000	−2.454184000
H	−8.542870000	1.172833000	−2.398770000
N	−4.101701000	0.579094000	0.907288000
H	−4.275356000	−0.186406000	1.275055000
H	−3.724644000	1.152821000	1.391436000
N	−5.199403000	1.055439000	0.250461000
N	−7.247357000	1.686489000	−1.198813000
O	−5.614495000	−1.935830000	1.443217000
O	−8.779139000	−0.847587000	−2.332053000
Pt	−6.687497000	−0.255182000	−0.531094000
C	7.206187000	−2.160821000	0.131300000
C	7.501650000	−3.384982000	−0.695293000
H	6.986773000	−3.347854000	−1.528634000
H	8.458211000	−3.415525000	−0.903391000
H	7.250307000	−4.187866000	−0.191987000
C	4.329118000	−2.719805000	−0.797094000
H	4.623962000	−2.764988000	0.136468000
H	3.352327000	−2.668062000	−0.829436000
H	4.629809000	−3.522661000	−1.273438000
C	4.919904000	−1.497663000	−1.453236000
C	6.619985000	2.435624000	−0.938001000
C	7.800446000	2.086798000	−0.123649000
C	6.285880000	3.850367000	−1.284692000
H	5.324197000	3.995076000	−1.173729000
H	6.779172000	4.457028000	−0.693453000
H	6.537489000	4.025788000	−2.215657000
C	8.756269000	3.128291000	0.373112000
H	9.673186000	2.786496000	0.308433000
H	8.670941000	3.938094000	−0.172141000
H	8.552669000	3.340900000	1.307576000
N	4.752732000	1.657879000	−2.067489000

H	4.864109000	2.326665000	−2.589203000
H	4.603780000	0.934697000	−2.533790000
N	9.044949000	0.340958000	0.772268000
H	8.871294000	−0.424542000	1.140035000
H	9.422006000	0.914685000	1.256417000
N	7.947246000	0.817304000	0.115441000
N	5.899293000	1.448353000	−1.333832000
O	7.532155000	−2.173965000	1.308197000
O	4.367512000	−1.085721000	−2.467073000
Pt	6.459153000	−0.493318000	−0.666113000
O---H interactions			
C	−1.655575000	2.229760000	0.957907000
C	−2.639900000	2.004322000	2.075675000
H	−2.871427000	2.864533000	2.484777000
H	−2.239026000	1.418872000	2.750802000
H	−3.450391000	1.584510000	1.717943000
C	−3.496432000	4.617567000	0.356639000
H	−3.368638000	3.856358000	−0.247299000
H	−3.896346000	5.362548000	−0.136227000
H	−4.090935000	4.355054000	1.091181000
C	−2.163935000	5.043596000	0.918896000
C	2.127786000	5.068159000	0.463911000
C	2.384416000	3.614932000	0.488654000
C	3.221659000	6.062887000	0.246476000
H	2.916504000	6.752099000	−0.377988000
H	4.007314000	5.608914000	−0.124456000
H	3.458637000	6.478714000	1.101873000
C	3.764085000	3.051153000	0.334583000
H	3.875205000	2.290532000	0.943557000
H	4.425918000	3.740942000	0.550300000
H	3.894279000	2.751187000	−0.588970000
N	0.544866000	6.754028000	0.603438000
H	1.171669000	7.232776000	0.934775000
H	−0.183139000	6.842250000	1.077613000
N	1.430394000	1.519807000	0.804413000
H	0.683634000	1.135506000	0.589791000
H	2.129956000	1.197756000	0.468808000
N	1.330578000	2.873858000	0.663489000
N	0.907062000	5.425917000	0.644534000
O	−1.476313000	1.320431000	0.162608000
O	−2.085641000	6.180818000	1.369775000
Pt	−0.541160000	3.878442000	0.837796000
C	−5.031682000	−0.484214000	−2.283424000
C	−4.054151000	0.654401000	−2.152599000
H	−3.829993000	0.784786000	−1.207248000
H	−4.456759000	1.473107000	−2.508792000
H	−3.238977000	0.444916000	−2.655310000
C	−3.199013000	−1.771247000	−0.177059000
H	−3.318574000	−2.116767000	−1.086362000
H	−2.799484000	−2.464614000	0.386380000
H	−2.608380000	−0.988621000	−0.200445000
C	−4.537655000	−1.371448000	0.389847000

C	−8.826112000	−1.834732000	0.244965000
C	−9.075371000	−1.372102000	−1.134232000
C	−9.923559000	−2.349051000	1.119268000
H	−9.617563000	−3.151417000	1.589015000
H	−10.704200000	−2.569040000	0.568685000
H	−10.168750000	−1.661127000	1.773040000
C	−10.450973000	−1.355236000	−1.727958000
H	−10.562460000	−0.544985000	−2.269074000
H	−11.117885000	−1.362128000	−1.009945000
H	−10.573056000	−2.145124000	−2.294573000
N	−7.252984000	−2.204439000	1.905039000
H	−7.884595000	−2.037015000	2.457219000
H	−6.528827000	−1.775526000	2.137947000
N	−8.112761000	−0.431349000	−3.028373000
H	−7.362515000	−0.515546000	−3.454334000
H	−8.808246000	−0.657203000	−3.441925000
N	−8.018972000	−0.975454000	−1.780002000
N	−7.608571000	−1.764688000	0.649280000
O	−5.200582000	−0.967475000	−3.392228000
O	−4.625041000	−1.286803000	1.609640000
Pt	−6.153750000	−1.104065000	−0.756584000
C	1.229130000	−2.525585000	4.463988000
C	0.251600000	−3.664199000	4.333163000
H	0.027442000	−3.794584000	3.387813000
H	0.654208000	−4.482906000	4.689356000
H	−0.563574000	−3.454714000	4.835875000
C	−0.603538000	−1.238552000	2.357623000
H	−0.483978000	−0.893031000	3.266927000
H	−1.003067000	−0.545185000	1.794185000
H	−1.194171000	−2.021178000	2.381010000
C	0.735104000	−1.638351000	1.790718000
C	5.023561000	−1.175067000	1.935600000
C	5.272820000	−1.637696000	3.314796000
C	6.121008000	−0.660748000	1.061297000
H	5.815011000	0.141618000	0.591550000
H	6.901649000	−0.440758000	1.611879000
H	6.366199000	−1.348671000	0.407525000
C	6.648421000	−1.654562000	3.908523000
H	6.759909000	−2.464814000	4.449638000
H	7.315334000	−1.647671000	3.190509000
H	6.770505000	−0.864675000	4.475138000
N	3.450433000	−0.805360000	0.275525000
H	4.082044000	−0.972784000	−0.276654000
H	2.726276000	−1.234273000	0.042617000
N	4.310209000	−2.578450000	5.208938000
H	3.559964000	−2.494253000	5.634899000
H	5.005695000	−2.352596000	5.622489000
N	4.216421000	−2.034344000	3.960567000
N	3.806019000	−1.245111000	1.531285000
O	1.398030000	−2.042323000	5.572793000
O	0.822489000	−1.722996000	0.570924000
Pt	2.351199000	−1.905734000	2.937148000

C	5.458126000	0.780038000	−3.138472000
C	6.442451000	1.005477000	−4.256240000
H	6.673979000	0.145265000	−4.665342000
H	6.041578000	1.590927000	−4.931367000
H	7.252942000	1.425289000	−3.898508000
C	7.298984000	−1.607768000	−2.537204000
H	7.171189000	−0.846559000	−1.933266000
H	7.698897000	−2.352749000	−2.044338000
H	7.893487000	−1.345255000	−3.271745000
C	5.966487000	−2.033797000	−3.099461000
C	1.674765000	−2.058360000	−2.644475000
C	1.418136000	−0.605133000	−2.669219000
C	0.580893000	−3.053089000	−2.427041000
H	0.886047000	−3.742300000	−1.802577000
H	−0.204762000	−2.599115000	−2.056108000
H	0.343914000	−3.468915000	−3.282438000
C	0.038466000	−0.041355000	−2.515147000
H	−0.072653000	0.719267000	−3.124122000
H	−0.623366000	−0.731143000	−2.730865000
H	−0.091727000	0.258611000	−1.591595000
N	3.257686000	−3.744230000	−2.784002000
H	2.630883000	−4.222977000	−3.115340000
H	3.985690000	−3.832452000	−3.258178000
N	2.372158000	1.489992000	−2.984977000
H	3.118917000	1.874293000	−2.770356000
H	1.672596000	1.812043000	−2.649373000
N	2.471973000	0.135941000	−2.844054000
N	2.895490000	−2.416118000	−2.825099000
O	5.278864000	1.689368000	−2.343172000
O	5.888193000	−3.171020000	−3.550340000
Pt	4.343712000	−0.868643000	−3.018361000
Pt---H interactions			
C	0.681768000	4.875237000	−0.245288000
C	−0.124875000	6.075064000	0.177634000
H	−0.945615000	5.775935000	0.622429000
H	−0.356250000	6.607612000	−0.611103000
H	0.402128000	6.621136000	0.798265000
C	−0.161435000	3.834368000	2.521932000
H	0.765181000	4.023518000	2.264801000
H	−0.170323000	3.274425000	3.324570000
H	−0.627422000	4.677412000	2.706407000
C	−0.858949000	3.112572000	1.396913000
C	−0.473268000	0.669081000	−2.139623000
C	0.318604000	1.584658000	−2.984015000
C	−0.842471000	−0.706652000	−2.591693000
H	−0.710559000	−1.338305000	−1.855690000
H	−0.277313000	−0.964168000	−3.350125000
H	−1.783103000	−0.717602000	−2.867130000
C	0.768137000	1.202929000	−4.361431000
H	0.687944000	1.977991000	−4.956906000
H	0.207528000	0.473291000	−4.698638000
H	1.702527000	0.909817000	−4.331740000

N	−1.538046000	0.348655000	−0.107177000
H	−2.076979000	−0.157443000	−0.537621000
H	−1.984436000	0.886389000	0.416459000
N	1.221104000	3.719624000	−3.147782000
H	1.609869000	4.275745000	−2.608345000
H	1.682880000	3.429217000	−3.786657000
N	0.581784000	2.738679000	−2.446147000
N	−0.829036000	1.128493000	−0.993694000
O	1.850098000	5.052326000	−0.553773000
O	−1.867382000	2.476395000	1.681586000
Pt	−0.133076000	3.069133000	−0.465553000
C	5.166374000	−3.279343000	−0.255074000
C	4.427308000	−4.531865000	−0.647619000
H	3.571287000	−4.289899000	−1.059463000
H	4.263855000	−5.077873000	0.148876000
H	4.965500000	−5.041624000	−1.289184000
C	4.146325000	−2.301995000	−2.985677000
H	5.093044000	−2.427282000	−2.765361000
H	4.067847000	−1.745030000	−3.786598000
H	3.731930000	−3.175052000	−3.152851000
C	3.445980000	−1.627853000	−1.833188000
C	3.802912000	0.840954000	1.688754000
C	4.687800000	−0.017460000	2.500194000
C	3.358936000	2.188965000	2.156805000
H	3.418557000	2.827148000	1.417038000
H	3.934579000	2.485373000	2.892818000
H	2.431276000	2.136225000	2.468959000
C	5.164012000	0.395829000	3.859418000
H	5.160199000	−0.382098000	4.456544000
H	4.568698000	1.086060000	4.219341000
H	6.074393000	0.751830000	3.793472000
N	2.639852000	1.085432000	−0.299910000
H	2.085025000	1.554242000	0.152029000
H	2.210900000	0.517857000	−0.806343000
N	5.739278000	−2.085814000	2.625562000
H	6.143529000	−2.614900000	2.070540000
H	6.204928000	−1.763790000	3.246211000
N	5.007621000	−1.151606000	1.950869000
N	3.434548000	0.356856000	0.557056000
O	6.355253000	−3.376074000	0.007086000
O	2.386185000	−1.062182000	−2.077215000
Pt	4.239742000	−1.532608000	−0.000586000
C	−3.234224000	−3.565725000	−0.089985000
C	−3.973291000	−4.818248000	−0.482530000
H	−4.829312000	−4.576281000	−0.894373000
H	−4.136744000	−5.364256000	0.313966000
H	−3.435098000	−5.328007000	−1.124095000
C	−4.254274000	−2.588378000	−2.820588000
H	−3.307555000	−2.713664000	−2.600272000
H	−4.332752000	−2.031412000	−3.621509000
H	−4.668669000	−3.461434000	−2.987761000
C	−4.954619000	−1.914235000	−1.668099000

C	−4.597687000	0.554572000	1.853844000
C	−3.712798000	−0.303843000	2.665284000
C	−5.041663000	1.902583000	2.321895000
H	−4.982042000	2.540766000	1.582128000
H	−4.466019000	2.198990000	3.057908000
H	−5.969323000	1.849843000	2.634048000
C	−3.236587000	0.109446000	4.024508000
H	−3.240400000	−0.668480000	4.621634000
H	−3.831900000	0.799678000	4.384431000
H	−2.326206000	0.465447000	3.958562000
N	−5.760747000	0.799049000	−0.134820000
H	−6.315574000	1.267860000	0.317118000
H	−6.189699000	0.231474000	−0.641254000
N	−2.661321000	−2.372197000	2.790651000
H	−2.257070000	−2.901283000	2.235630000
H	−2.195671000	−2.050173000	3.411300000
N	−3.392978000	−1.437988000	2.115959000
N	−4.966051000	0.070474000	0.722146000
O	−2.045346000	−3.662456000	0.172176000
O	−6.014414000	−1.348564000	−1.912125000
Pt	−4.160857000	−1.818991000	0.164503000

**Complex 4**			
C---H interactions			
C	6.758026000	−2.091813000	−0.089591000
C	7.170732000	−3.471573000	0.336714000
H	6.844509000	−3.644295000	1.244629000
H	8.149034000	−3.539906000	0.322243000
H	6.788851000	−4.131550000	−0.278588000
C	4.207612000	−3.278057000	0.344379000
C	3.896475000	−4.154297000	−0.831585000
H	4.665478000	−4.170825000	−1.438795000
H	3.114750000	−3.801992000	−1.304357000
H	3.707188000	−5.064041000	−0.520033000
C	4.250950000	1.441570000	−0.196981000
H	4.328272000	2.382398000	−0.302175000
C	2.962528000	0.803186000	0.046584000
C	1.791494000	1.549941000	0.163927000
H	1.805215000	2.496141000	0.076576000
C	0.607673000	0.876635000	0.410233000
H	−0.203983000	1.361054000	0.501265000
C	0.606492000	−0.483342000	0.523277000
H	−0.201212000	−0.954556000	0.691819000
C	1.808279000	−1.165010000	0.385882000
H	1.807446000	−2.112326000	0.457726000
C	7.086273000	2.370557000	−0.223268000
C	8.362884000	2.595293000	−0.764156000
H	8.748056000	1.955032000	−1.352184000
C	9.057542000	3.738852000	−0.445278000
H	9.924089000	3.876377000	−0.810349000
C	8.515505000	4.692512000	0.400161000
H	9.006713000	5.473231000	0.627849000

C	7.246685000	4.487542000	0.904763000
H	6.859470000	5.146387000	1.470420000
C	6.526423000	3.341660000	0.603664000
H	5.654020000	3.219416000	0.959470000
N	5.286578000	0.663683000	−0.262292000
N	2.964227000	−0.538570000	0.155785000
N	6.534630000	1.108724000	−0.506386000
H	7.123979000	0.372908000	−0.609090000
O	7.662809000	−1.344621000	−0.471570000
O	3.950507000	−3.686159000	1.452137000
Pt	4.876469000	−1.439615000	0.008103000
C	−3.329908000	−1.411866000	−0.206565000
C	−2.917202000	−2.791627000	0.219739000
H	−3.243425000	−2.964348000	1.127654000
H	−1.938900000	−2.859959000	0.205269000
H	−3.299084000	−3.451604000	−0.395562000
C	−5.880322000	−2.598110000	0.227403000
C	−6.191459000	−3.474350000	−0.948560000
H	−5.422458000	−3.490878000	−1.555770000
H	−6.973186000	−3.122046000	−1.421331000
H	−6.380746000	−4.384094000	−0.637007000
C	−5.836985000	2.121517000	−0.313956000
H	−5.759663000	3.062345000	−0.419150000
C	−7.125406000	1.483133000	−0.070391000
C	−8.296441000	2.229888000	0.046952000
H	−8.282719000	3.176088000	−0.040398000
C	−9.480261000	1.556582000	0.293258000
H	−10.291917000	2.041000000	0.384290000
C	−9.481443000	0.196605000	0.406302000
H	−10.289147000	−0.274609000	0.574845000
C	−8.279655000	−0.485063000	0.268907000
H	−8.280489000	−1.432379000	0.340751000
C	−3.001661000	3.050504000	−0.340242000
C	−1.725050000	3.275240000	−0.881131000
H	−1.339878000	2.634979000	−1.469159000
C	−1.030392000	4.418798000	−0.562253000
H	−0.163845000	4.556323000	−0.927323000
C	−1.572429000	5.372459000	0.283187000
H	−1.081221000	6.153178000	0.510875000
C	−2.841249000	5.167489000	0.787788000
H	−3.228465000	5.826334000	1.353444000
C	−3.561511000	4.021607000	0.486690000
H	−4.433915000	3.899363000	0.842495000
N	−4.801356000	1.343630000	−0.379267000
N	−7.123707000	0.141377000	0.038810000
N	−3.553304000	1.788671000	−0.623361000
H	−2.963956000	1.052855000	−0.726066000
O	−2.425125000	−0.664674000	−0.588546000
O	−6.137427000	−3.006212000	1.335163000
Pt	−5.211465000	−0.759668000	−0.108871000
O---H interactions			
C	−0.851121000	−1.360248000	1.076960000

C	−1.489619000	−0.057231000	0.689317000
H	−2.108821000	−0.205692000	−0.055742000
H	−0.795250000	0.579070000	0.415593000
H	−1.981161000	0.306277000	1.454985000
C	−3.589853000	−1.970042000	1.554046000
C	−3.988177000	−1.334500000	2.852090000
H	−3.241052000	−0.803056000	3.198085000
H	−4.220725000	−2.031125000	3.499671000
H	−4.762892000	−0.752143000	2.706466000
C	−0.595070000	−5.657962000	1.572791000
H	0.052391000	−6.352667000	1.553277000
C	−2.009149000	−5.937272000	1.794727000
C	−2.475969000	−7.240191000	1.959698000
H	−1.878183000	−7.978735000	1.941857000
C	−3.833476000	−7.429852000	2.151118000
H	−4.179624000	−8.307426000	2.259647000
C	−4.675068000	−6.356063000	2.183038000
H	−5.608439000	−6.476072000	2.314381000
C	−4.140644000	−5.084830000	2.021184000
H	−4.724802000	−4.336164000	2.049963000
C	2.081172000	−4.715457000	0.649658000
C	3.330514000	−4.082396000	0.755391000
H	3.415261000	−3.282469000	1.262346000
C	4.431648000	−4.616238000	0.127317000
H	5.270149000	−4.174739000	0.198650000
C	4.338222000	−5.785820000	−0.608474000
H	5.099636000	−6.141244000	−1.051839000
C	3.117339000	−6.426333000	−0.684162000
H	3.048820000	−7.240024000	−1.171187000
C	1.990358000	−5.907641000	−0.065004000
H	1.159336000	−6.363868000	−0.127349000
N	−0.263486000	−4.415818000	1.402412000
N	−2.836626000	−4.875933000	1.827565000
N	1.005921000	−4.005998000	1.212853000
H	1.040450000	−3.058322000	1.207941000
O	0.382658000	−1.381211000	1.090619000
O	−4.357184000	−1.909987000	0.622716000
Pt	−1.898702000	−3.007071000	1.484037000
C	−9.786018000	6.281513000	0.676664000
C	−10.730427000	6.966217000	1.622747000
H	−10.237030000	7.272656000	2.412189000
H	−11.430787000	6.337438000	1.898585000
H	−11.140758000	7.735228000	1.175255000
C	−8.632644000	8.882106000	0.758882000
C	−9.459147000	9.784629000	−0.107050000
H	−10.237587000	9.292507000	−0.441997000
H	−8.920256000	10.096565000	−0.862707000
H	−9.761398000	10.554660000	0.418521000
C	−5.986361000	5.486311000	−1.249995000
H	−5.503486000	4.761607000	−1.629256000
C	−5.417516000	6.827016000	−1.173737000
C	−4.128064000	7.096922000	−1.628927000

H	−3.591972000	6.411610000	−2.011111000
C	−3.649284000	8.390036000	−1.510338000
H	−2.771869000	8.600477000	−1.806294000
C	−4.437437000	9.362784000	−0.967294000
H	−4.117840000	10.253534000	−0.882103000
C	−5.715797000	9.025740000	−0.542985000
H	−6.267694000	9.703666000	−0.170970000
C	−7.316329000	2.853235000	−0.801818000
C	−8.256878000	1.832409000	−1.016491000
H	−9.154999000	2.053870000	−1.236493000
C	−7.882391000	0.513256000	−0.909007000
H	−8.529190000	−0.168971000	−1.047971000
C	−6.578570000	0.162897000	−0.600236000
H	−6.330007000	−0.749949000	−0.513184000
C	−5.646331000	1.165871000	−0.422716000
H	−4.744832000	0.933554000	−0.229562000
C	−5.997693000	2.503593000	−0.520325000
H	−5.341490000	3.179348000	−0.396304000
N	−7.182786000	5.330182000	−0.774505000
N	−6.197998000	7.784856000	−0.639297000
N	−7.843863000	4.156458000	−0.799560000
H	−8.732613000	4.281462000	−0.493286000
O	−10.090421000	5.135420000	0.335127000
O	−8.245995000	9.300088000	1.824516000
Pt	−8.089290000	7.113174000	0.040573000
C	−4.062738000	−8.617169000	−1.055216000
C	−3.424239000	−9.920186000	−0.667573000
H	−2.805038000	−9.771725000	0.077485000
H	−4.118609000	−10.556487000	−0.393849000
H	−2.932697000	−10.283693000	−1.433242000
C	−1.324006000	−8.007375000	−1.532303000
C	−0.925682000	−8.642916000	−2.830346000
H	−1.672807000	−9.174361000	−3.176342000
H	−0.693134000	−7.946291000	−3.477927000
H	−0.150967000	−9.225274000	−2.684722000
C	−4.318788000	−4.319454000	−1.551048000
H	−4.966250000	−3.624749000	−1.531533000
C	−2.904710000	−4.040145000	−1.772983000
C	−2.437890000	−2.737225000	−1.937954000
H	−3.035676000	−1.998682000	−1.920113000
C	−1.080383000	−2.547564000	−2.129374000
H	−0.734235000	−1.669991000	−2.237903000
C	−0.238790000	−3.621353000	−2.161294000
H	0.694581000	−3.501344000	−2.292638000
C	−0.773215000	−4.892587000	−1.999440000
H	−0.189056000	−5.641252000	−2.028219000
C	−6.995031000	−5.261959000	−0.627914000
C	−8.244373000	−5.895021000	−0.733647000
H	−8.329120000	−6.694947000	−1.240602000
C	−9.345507000	−5.361179000	−0.105573000
H	−10.184008000	−5.802677000	−0.176906000
C	−9.252081000	−4.191596000	0.630218000

H	−10.013496000	−3.836172000	1.073583000
C	−8.031198000	−3.551084000	0.705906000
H	−7.962679000	−2.737393000	1.192930000
C	−6.904217000	−4.069775000	0.086747000
H	−6.073194000	−3.613548000	0.149093000
N	−4.650373000	−5.561599000	−1.380668000
N	−2.077233000	−5.101484000	−1.805821000
N	−5.919780000	−5.971419000	−1.191109000
H	−5.954309000	−6.919094000	−1.186199000
O	−5.296517000	−8.596206000	−1.068876000
O	−0.556675000	−8.067430000	−0.600972000
Pt	−3.015157000	−6.970346000	−1.462294000
C	6.173675000	−1.380973000	2.203430000
C	6.812174000	−2.683990000	2.591072000
H	7.431376000	−2.535529000	3.336132000
H	6.117804000	−3.320291000	2.864797000
H	7.303716000	−3.047497000	1.825405000
C	8.912407000	−0.771179000	1.726344000
C	9.310731000	−1.406721000	0.428301000
H	8.563606000	−1.938165000	0.082304000
H	9.543279000	−0.710095000	−0.219280000
H	10.085446000	−1.989078000	0.573925000
C	5.917624000	2.916741000	1.707598000
H	5.270163000	3.611447000	1.727114000
C	7.331703000	3.196050000	1.485664000
C	7.798523000	4.498970000	1.320693000
H	7.200737000	5.237513000	1.338534000
C	9.156030000	4.688631000	1.129273000
H	9.502179000	5.566205000	1.020744000
C	9.997623000	3.614843000	1.097353000
H	10.930994000	3.734851000	0.966008000
C	9.463197000	2.343608000	1.259206000
H	10.047357000	1.594944000	1.230427000
C	3.241383000	1.974237000	2.630733000
C	1.992040000	1.341174000	2.525000000
H	1.907293000	0.541248000	2.018045000
C	0.890907000	1.875017000	3.153074000
H	0.052405000	1.433518000	3.081741000
C	0.984332000	3.044599000	3.888864000
H	0.222917000	3.400023000	4.332229000
C	2.205215000	3.685112000	3.964553000
H	2.273734000	4.498803000	4.451577000
C	3.332196000	3.166421000	3.345394000
H	4.163219000	3.622648000	3.407740000
N	5.586040000	1.674597000	1.877979000
N	8.159180000	2.134712000	1.452825000
N	4.316633000	1.264776000	2.067538000
H	4.282104000	0.317101000	2.072448000
O	4.939896000	−1.360010000	2.189771000
O	9.679738000	−0.831234000	2.657675000
Pt	7.221256000	0.265849000	1.796353000
C	6.243551000	5.154141000	−2.000507000

C	5.605052000	6.457158000	−2.388149000
H	4.985850000	6.308697000	−3.133209000
H	6.299421000	7.093460000	−2.661874000
H	5.113510000	6.820666000	−1.622481000
C	3.504819000	4.544348000	−1.523421000
C	3.106494000	5.179889000	−0.225376000
H	3.853620000	5.711334000	0.120619000
H	2.873947000	4.483264000	0.422204000
H	2.331779000	5.762246000	−0.371001000
C	6.499601000	0.856427000	−1.504675000
H	7.147063000	0.161722000	−1.524190000
C	5.085523000	0.577118000	−1.282740000
C	4.618702000	−0.725802000	−1.117770000
H	5.216488000	−1.464345000	−1.135610000
C	3.261196000	−0.915462000	−0.926349000
H	2.915047000	−1.793037000	−0.817821000
C	2.419603000	0.158326000	−0.894430000
H	1.486232000	0.038317000	−0.763085000
C	2.954027000	1.429560000	−1.056283000
H	2.369869000	2.178225000	−1.027504000
C	9.175843000	1.798932000	−2.427810000
C	10.425186000	2.431994000	−2.322076000
H	10.509933000	3.231921000	−1.815121000
C	11.526319000	1.898152000	−2.950150000
H	12.364821000	2.339650000	−2.878817000
C	11.432894000	0.728569000	−3.685941000
H	12.194308000	0.373146000	−4.129306000
C	10.212011000	0.088057000	−3.761629000
H	10.143491000	−0.725634000	−4.248654000
C	9.085030000	0.606748000	−3.142470000
H	8.254007000	0.150521000	−3.204816000
N	6.831185000	2.098571000	−1.675055000
N	4.258045000	1.638457000	−1.249902000
N	8.100593000	2.508392000	−1.864614000
H	8.135121000	3.456067000	−1.869525000
O	7.477329000	5.133178000	−1.986848000
O	2.737488000	4.604403000	−2.454751000
Pt	5.195970000	3.507319000	−1.593430000

Complex 5			
C---H interactions			
C	4.834963000	−2.154228000	0.183921000
C	6.063242000	−2.611548000	0.925404000
H	6.863679000	−2.325986000	0.438829000
H	6.056319000	−3.589546000	0.996624000
H	6.068588000	−2.218939000	1.822976000
C	5.905842000	0.365368000	0.919408000
C	7.124016000	0.735209000	0.115503000
H	7.527077000	−0.077489000	−0.258198000
H	7.773000000	1.184719000	0.694874000
H	6.864251000	1.335051000	−0.614224000
C	1.837734000	0.445462000	−1.566671000

H	1.030720000	0.381038000	−2.064028000
C	2.455645000	1.741317000	−1.255795000
C	1.924388000	2.926486000	−1.699673000
H	1.128819000	2.925578000	−2.219332000
C	2.542537000	4.119278000	−1.393211000
H	2.191779000	4.944237000	−1.706765000
C	3.690000000	4.081821000	−0.615060000
H	4.136743000	4.885913000	−0.378647000
C	4.176916000	2.861702000	−0.187138000
H	4.951977000	2.848414000	0.361461000
C	0.745737000	−2.263471000	−1.678033000
C	0.545600000	−3.756187000	−1.624887000
H	−0.055993000	−3.980339000	−0.884018000
H	1.410205000	−4.197331000	−1.487871000
H	0.152036000	−4.063904000	−2.468028000
N	2.451943000	−0.610698000	−1.125349000
N	3.601601000	1.698057000	−0.514019000
N	1.984409000	−1.884293000	−1.286837000
H	2.570606000	−2.459023000	−1.058271000
O	4.145026000	−3.078912000	−0.315547000
O	5.944240000	0.527466000	2.141896000
O	−0.118101000	−1.488793000	−2.015219000
Pt	4.302642000	−0.259580000	−0.053429000
C	−2.606852000	−1.929389000	0.967196000
C	−1.378574000	−2.386709000	1.708679000
H	−0.578136000	−2.101147000	1.222105000
H	−1.385497000	−3.364708000	1.779899000
H	−1.373227000	−1.994101000	2.606251000
C	−1.535973000	0.590207000	1.702683000
C	−0.317799000	0.960048000	0.898778000
H	0.085261000	0.147349000	0.525078000
H	0.331185000	1.409558000	1.478149000
H	−0.577564000	1.559890000	0.169051000
C	−5.604081000	0.670301000	−0.783396000
H	−6.411096000	0.605876000	−1.280753000
C	−4.986170000	1.966156000	−0.472520000
C	−5.517428000	3.151325000	−0.916398000
H	−6.312996000	3.150417000	−1.436057000
C	−4.899279000	4.344116000	−0.609936000
H	−5.250036000	5.169076000	−0.923490000
C	−3.751816000	4.306660000	0.168216000
H	−3.305072000	5.110751000	0.404628000
C	−3.264899000	3.086541000	0.596137000
H	−2.489838000	3.073253000	1.144736000
C	−6.696078000	−2.038633000	−0.894758000
C	−6.896216000	−3.531348000	−0.841612000
H	−7.497808000	−3.755500000	−0.100742000
H	−6.031611000	−3.972493000	−0.704596000
H	−7.289779000	−3.839066000	−1.684753000
N	−4.989872000	−0.385860000	−0.342074000
N	−3.840215000	1.922896000	0.269256000
N	−5.457407000	−1.659455000	−0.503562000

H	−4.871209000	−2.234184000	−0.274996000
O	−3.296789000	−2.854073000	0.467728000
O	−1.497576000	0.752305000	2.925171000
O	−7.559917000	−1.263954000	−1.231944000
Pt	−3.139173000	−0.034742000	0.729846000
O---H interactions			
C	−1.260094000	−0.098971000	−0.285793000
C	−1.499502000	−1.459607000	−0.930862000
H	−0.988090000	−1.520814000	−1.764522000
H	−2.454592000	−1.565378000	−1.124410000
H	−1.211448000	−2.167246000	−0.316942000
C	1.403119000	−1.059779000	−0.441309000
C	1.836135000	−0.984718000	−1.884017000
H	1.073735000	−0.719375000	−2.439696000
H	2.161834000	−1.861832000	−2.173013000
H	2.553803000	−0.322920000	−1.976113000
C	0.735692000	3.018275000	1.928164000
H	0.526099000	3.824751000	2.386345000
C	2.101832000	2.520577000	1.836602000
C	3.160008000	3.217326000	2.382748000
H	3.015678000	4.049867000	2.816617000
C	4.441312000	2.681328000	2.288515000
H	5.183035000	3.154054000	2.647051000
C	4.627592000	1.460667000	1.671003000
H	5.490618000	1.067118000	1.617107000
C	3.531865000	0.835259000	1.137946000
H	3.659736000	−0.005088000	0.712783000
C	−2.091857000	3.730231000	1.903432000
C	−3.593094000	3.727899000	1.778935000
H	−3.868449000	3.013144000	1.168270000
H	−3.892770000	4.592131000	1.427295000
H	−3.993976000	3.577332000	2.660821000
N	−0.164179000	2.314320000	1.361311000
N	2.298978000	1.332026000	1.178303000
N	−1.502003000	2.631355000	1.366626000
H	−2.019966000	2.067187000	0.873081000
O	−2.295020000	0.574782000	−0.138582000
O	1.664995000	−2.078816000	0.196387000
O	−1.473507000	4.627518000	2.463254000
Pt	0.507577000	0.509272000	0.363289000
C	9.089341000	−2.629130000	−0.383284000
C	9.316399000	−1.151267000	−0.562095000
H	8.960570000	−0.868797000	−1.429618000
H	10.277936000	−0.962088000	−0.526627000
H	8.860730000	−0.661380000	0.153575000
C	6.398386000	−1.737655000	−0.394200000
C	5.925551000	−1.336351000	−1.766197000
H	6.700680000	−1.124572000	−2.329149000
H	5.348878000	−0.548147000	−1.694829000
H	5.423501000	−2.075469000	−2.167993000
C	7.065469000	−6.429018000	0.137424000
H	7.277436000	−7.349798000	0.237530000

C	5.677280000	−5.959303000	0.037433000
C	4.613123000	−6.826151000	0.042757000
H	4.762451000	−7.761423000	0.119859000
C	3.327022000	−6.343004000	−0.063503000
H	2.584017000	−6.934370000	−0.075083000
C	3.147787000	−4.970617000	−0.153083000
H	2.274426000	−4.603392000	−0.219571000
C	4.253762000	−4.142581000	−0.144730000
H	4.119112000	−3.203454000	−0.186771000
C	9.899620000	−6.927694000	0.649369000
C	11.397471000	−6.820282000	0.775801000
H	11.646238000	−6.802709000	1.723879000
H	11.703865000	−5.995894000	0.342869000
H	11.818676000	−7.592238000	0.343206000
N	7.990982000	−5.519123000	0.082547000
N	5.506707000	−4.609337000	−0.080101000
N	9.325300000	−5.784324000	0.208938000
H	9.795110000	−5.100184000	0.015511000
O	10.134976000	−3.327090000	−0.378606000
O	6.141512000	−0.988607000	0.551934000
O	9.278689000	−7.924914000	0.932272000
Pt	7.309161000	−3.480768000	−0.196040000
C	3.534777000	6.563134000	0.419643000
C	3.774185000	7.923770000	1.064713000
H	3.262773000	7.984977000	1.898372000
H	4.729277000	8.029542000	1.258260000
H	3.486133000	8.631409000	0.450791000
C	0.871565000	7.523942000	0.575159000
C	0.438548000	7.448881000	2.017867000
H	1.200949000	7.183538000	2.573545000
H	0.112850000	8.325995000	2.306862000
H	−0.279119000	6.787083000	2.109963000
C	1.538992000	3.445889000	−1.794314000
H	1.748585000	2.639412000	−2.252495000
C	0.172853000	3.943585000	−1.702752000
C	−0.885324000	3.246837000	−2.248899000
H	−0.740993000	2.414296000	−2.682767000
C	−2.166628000	3.782835000	−2.154665000
H	−2.908351000	3.310109000	−2.513201000
C	−2.352909000	5.003496000	−1.537153000
H	−3.215934000	5.397045000	−1.483257000
C	−1.257181000	5.628904000	−1.004096000
H	−1.385053000	6.469250000	−0.578933000
C	4.366541000	2.733931000	−1.769581000
C	5.867779000	2.736264000	−1.645086000
H	6.143133000	3.451019000	−1.034420000
H	6.167455000	1.872032000	−1.293445000
H	6.268661000	2.886830000	−2.526971000
N	2.438863000	4.149843000	−1.227462000
N	−0.024294000	5.132137000	−1.044454000
N	3.776686000	3.832808000	−1.232776000
H	4.294649000	4.396976000	−0.739230000

O	4.569704000	5.889381000	0.272431000
O	0.609689000	8.542979000	−0.062538000
O	3.748191000	1.836645000	−2.329404000
Pt	1.767106000	5.954892000	−0.229439000
C	−11.364024000	−3.835033000	0.249435000
C	−11.591083000	−5.312895000	0.428246000
H	−11.235254000	−5.595366000	1.295768000
H	−12.552620000	−5.502075000	0.392776000
H	−11.135414000	−5.802783000	−0.287425000
C	−8.673069000	−4.726507000	0.260350000
C	−8.200234000	−5.127812000	1.632347000
H	−8.975364000	−5.339591000	2.195299000
H	−7.623562000	−5.916016000	1.560979000
H	−7.698185000	−4.388693000	2.034144000
C	−9.340153000	−0.035144000	−0.271273000
H	−9.552120000	0.885635000	−0.371380000
C	−7.951965000	−0.504860000	−0.171283000
C	−6.887806000	0.361989000	−0.176607000
H	−7.037134000	1.297260000	−0.253709000
C	−5.601706000	−0.121160000	−0.070347000
H	−4.858700000	0.470208000	−0.058767000
C	−5.422472000	−1.493547000	0.019234000
H	−4.549110000	−1.860771000	0.085721000
C	−6.528446000	−2.321582000	0.010880000
H	−6.393795000	−3.260708000	0.052922000
C	−12.174303000	0.463531000	−0.783219000
C	−13.672154000	0.356119000	−0.909652000
H	−13.920921000	0.338547000	−1.857729000
H	−13.978548000	−0.468269000	−0.476719000
H	−14.093359000	1.128076000	−0.477056000
N	−10.265665000	−0.945039000	−0.216397000
N	−7.781392000	−1.854826000	−0.053749000
N	−11.599983000	−0.679839000	−0.342788000
H	−12.069793000	−1.363980000	−0.149360000
O	−12.409660000	−3.137073000	0.244756000
O	−8.416195000	−5.475556000	−0.685784000
O	−11.553373000	1.460752000	−1.066122000
Pt	−9.583845000	−2.983395000	0.062190000
O---H interactions			
C	−0.720200000	−6.558706000	−1.651860000
C	0.340904000	−7.612575000	−1.827999000
H	0.869729000	−7.413102000	−2.627720000
H	−0.084397000	−8.490401000	−1.928706000
H	0.926392000	−7.621719000	−1.042552000
C	1.592565000	−4.969421000	−1.250140000
C	2.339213000	−4.697570000	−2.529049000
H	2.106346000	−5.380409000	−3.193951000
H	3.302714000	−4.725270000	−2.356400000
H	2.091850000	−3.813138000	−2.870198000
C	−2.597963000	−2.717144000	−0.928845000
H	−3.468221000	−2.338698000	−0.881783000
C	−1.387031000	−1.893545000	−0.814712000

C	−1.444815000	−0.529431000	−0.674204000
H	−2.286899000	−0.090370000	−0.641098000
C	−0.283273000	0.206015000	−0.580841000
H	−0.310942000	1.151637000	−0.497550000
C	0.926137000	−0.472185000	−0.611932000
H	1.745482000	0.003020000	−0.542027000
C	0.926665000	−1.847302000	−0.745893000
H	1.759365000	−2.303886000	−0.746372000
C	−4.736206000	−4.708219000	−0.846898000
C	−5.555470000	−5.969825000	−0.937659000
H	−5.790113000	−6.274190000	−0.035800000
H	−5.035173000	−6.665605000	−1.391646000
H	−6.375178000	−5.792058000	−1.444557000
N	−2.418120000	−3.992611000	−1.097175000
N	−0.197670000	−2.562182000	−0.875226000
N	−3.436878000	−4.899884000	−1.172669000
H	−3.150968000	−5.664634000	−1.416793000
O	−1.898974000	−6.963938000	−1.815567000
O	2.241739000	−5.315843000	−0.259791000
O	−5.191072000	−3.644080000	−0.499417000
Pt	−0.359045000	−4.656542000	−1.226737000
C	2.260005000	3.628559000	0.264401000
C	3.132960000	2.551323000	0.898649000
H	3.397619000	2.833099000	1.799113000
H	2.628280000	1.712779000	0.953323000
H	3.933230000	2.414411000	0.349604000
C	4.593831000	5.136982000	0.828276000
C	4.638662000	5.376712000	2.316584000
H	3.914606000	4.877528000	2.749018000
H	5.500536000	5.074655000	2.669942000
H	4.528808000	6.334232000	2.497114000
C	1.199474000	7.315769000	−1.705815000
H	0.480917000	7.684249000	−2.207973000
C	2.399249000	8.085411000	−1.404796000
C	2.531920000	9.398551000	−1.806708000
H	1.828860000	9.831864000	−2.275906000
C	3.711435000	10.077737000	−1.514534000
H	3.814324000	10.986230000	−1.771877000
C	4.729149000	9.424306000	−0.848712000
H	5.550541000	9.863105000	−0.660644000
C	4.518690000	8.125925000	−0.466849000
H	5.219095000	7.675783000	−0.008483000
C	−1.053044000	5.508674000	−2.109668000
C	−1.957824000	4.308066000	−2.205240000
H	−1.617118000	3.596108000	−1.625030000
H	−2.861923000	4.557261000	−1.920882000
H	−1.982889000	3.990655000	−3.132451000
N	1.164558000	6.121264000	−1.260190000
N	3.393524000	7.455785000	−0.697785000
N	0.116398000	5.256028000	−1.467645000
H	0.205192000	4.455302000	−1.042071000
O	1.123002000	3.232731000	−0.047421000

O	5.629103000	4.793710000	0.259023000
O	−1.340424000	6.596757000	−2.593785000
Pt	2.896341000	5.459503000	−0.133908000
C	−8.984008000	2.848002000	1.376904000
C	−10.045112000	3.901871000	1.553042000
H	−10.573937000	3.702398000	2.352764000
H	−9.619811000	4.779697000	1.653750000
H	−10.630600000	3.911015000	0.767596000
C	−11.296773000	1.258717000	0.975184000
C	−12.043421000	0.986866000	2.254093000
H	−11.810554000	1.669705000	2.918994000
H	−13.006922000	1.014566000	2.081444000
H	−11.796058000	0.102434000	2.595241000
C	−7.106245000	−0.993560000	0.653889000
H	−6.235987000	−1.372007000	0.606826000
C	−8.317177000	−1.817159000	0.539755000
C	−8.259393000	−3.181273000	0.399248000
H	−7.417309000	−3.620334000	0.366142000
C	−9.420935000	−3.916719000	0.305885000
H	−9.393266000	−4.862342000	0.222594000
C	−10.630345000	−3.238519000	0.336976000
H	−11.449691000	−3.713725000	0.267070000
C	−10.630873000	−1.863402000	0.470937000
H	−11.463573000	−1.406818000	0.471416000
C	−4.968002000	0.997515000	0.571942000
C	−4.148738000	2.259120000	0.662703000
H	−3.914095000	2.563486000	−0.239156000
H	−4.669035000	2.954901000	1.116690000
H	−3.329030000	2.081354000	1.169601000
N	−7.286088000	0.281907000	0.822219000
N	−9.506538000	−1.148522000	0.600270000
N	−6.267330000	1.189180000	0.897713000
H	−6.553240000	1.953930000	1.141836000
O	−7.805234000	3.253234000	1.540611000
O	−11.945947000	1.605139000	−0.015166000
O	−4.513136000	−0.066624000	0.224461000
Pt	−9.345163000	0.945838000	0.951780000
C	7.444204000	0.082145000	0.010556000
C	6.571248000	1.159382000	−0.623693000
H	6.306589000	0.877605000	−1.524157000
H	7.075927000	1.997925000	−0.678367000
H	5.770978000	1.296293000	−0.074649000
C	5.110377000	−1.426278000	−0.553319000
C	5.065547000	−1.666007000	−2.041628000
H	5.789602000	−1.166824000	−2.474062000
H	4.203672000	−1.363951000	−2.394986000
H	5.175401000	−2.623528000	−2.222157000
C	8.504735000	−3.605064000	1.980771000
H	9.223290000	−3.973545000	2.482928000
C	7.304959000	−4.374707000	1.679753000
C	7.172288000	−5.687847000	2.081664000
H	7.875348000	−6.121159000	2.550862000

C	5.992774000	−6.367033000	1.789490000
H	5.889884000	−7.275526000	2.046833000
C	4.975059000	−5.713601000	1.123668000
H	4.153668000	−6.152401000	0.935600000
C	5.185518000	−4.415221000	0.741806000
H	4.485113000	−3.965078000	0.283439000
C	10.757252000	−1.797969000	2.384624000
C	11.662032000	−0.597361000	2.480196000
H	11.321325000	0.114596000	1.899986000
H	12.566131000	−0.846557000	2.195838000
H	11.687096000	−0.279951000	3.407408000
N	8.539650000	−2.410559000	1.535146000
N	6.310684000	−3.745081000	0.972741000
N	9.587811000	−1.545323000	1.742602000
H	9.499017000	−0.744598000	1.317028000
O	8.581206000	0.477973000	0.322377000
O	4.075105000	−1.083006000	0.015933000
O	11.044632000	−2.886053000	2.868740000
Pt	6.807868000	−1.748799000	0.408865000

**Complex 6**			
C---H interactions			
C	−6.838245000	−2.097726000	−1.169427000
C	−7.323829000	−2.890765000	−2.348177000
H	−7.346045000	−2.316325000	−3.141340000
H	−8.223861000	−3.230454000	−2.164745000
H	−6.716007000	−3.644708000	−2.506882000
C	−9.141331000	−0.603541000	−1.816277000
C	−10.399607000	−1.239805000	−1.292271000
H	−10.646163000	−0.821431000	−0.440676000
H	−10.246740000	−2.197537000	−1.152990000
H	−11.123211000	−1.115002000	−1.940266000
C	−6.296614000	1.268067000	1.511218000
H	−5.686066000	1.583362000	2.166630000
C	−7.490764000	2.015130000	1.165384000
C	−7.772656000	3.241802000	1.755462000
H	−7.185790000	3.611344000	2.404903000
C	−8.919885000	3.915763000	1.381084000
H	−9.134898000	4.753672000	1.773950000
C	−9.747764000	3.360802000	0.433641000
H	−10.542751000	3.807219000	0.168703000
C	−9.406802000	2.144213000	−0.126484000
H	−9.977710000	1.770835000	−0.787299000
C	−3.818681000	−0.270623000	1.632070000
C	−2.732821000	−1.291448000	1.574741000
C	−2.925796000	−2.623627000	1.210283000
H	−3.796467000	−2.929078000	0.986296000
C	−1.861668000	−3.504561000	1.170441000
H	−1.987832000	−4.408693000	0.909126000
C	−0.610775000	−3.035417000	1.520286000
C	−0.379362000	−1.734719000	1.896341000
H	0.491869000	−1.443545000	2.137918000

C	−1.447729000	−0.862723000	1.913504000
H	−1.307176000	0.044387000	2.160425000
F	0.440036000	−3.878274000	1.457049000
N	−6.103089000	0.151381000	0.883321000
N	−8.296533000	1.467060000	0.225886000
N	−5.015628000	−0.645116000	1.082122000
H	−5.108068000	−1.381229000	0.728179000
O	−5.857594000	−2.583853000	−0.573103000
O	−9.116057000	−0.184854000	−2.963692000
O	−3.642378000	0.834782000	2.121389000
Pt	−7.631998000	−0.390366000	−0.557272000
C	5.401598000	−1.562038000	0.214365000
C	4.602793000	−2.691821000	0.797165000
H	4.281268000	−2.439167000	1.687317000
H	3.838731000	−2.885329000	0.215913000
H	5.169779000	−3.489665000	0.868119000
C	2.924400000	−0.255039000	0.546714000
C	1.955562000	−0.580130000	−0.557071000
H	1.980496000	0.130120000	−1.232321000
H	2.206704000	−1.432207000	−0.970483000
H	1.051116000	−0.649749000	−0.188011000
C	6.589418000	2.498468000	−0.739132000
H	7.361962000	2.987925000	−0.994778000
C	5.303276000	3.146630000	−0.567605000
C	5.156454000	4.518517000	−0.737164000
H	5.901277000	5.057879000	−0.976069000
C	3.909606000	5.085377000	−0.551278000
H	3.784790000	6.020256000	−0.665678000
C	2.850625000	4.281960000	−0.199011000
H	1.986082000	4.653599000	−0.072772000
C	3.062348000	2.926428000	−0.032306000
H	2.328996000	2.378407000	0.220461000
C	9.058825000	0.950611000	−0.570330000
C	10.126287000	−0.089993000	−0.524187000
C	9.908182000	−1.449189000	−0.747315000
H	9.033920000	−1.758499000	−0.951114000
C	10.952115000	−2.351832000	−0.673592000
H	10.803963000	−3.279604000	−0.811244000
C	12.215573000	−1.869180000	−0.394411000
C	12.475707000	−0.537606000	−0.179871000
H	13.357259000	−0.235335000	0.003659000
C	11.420802000	0.348863000	−0.238421000
H	11.577510000	1.273341000	−0.082353000
F	13.236768000	−2.743878000	−0.291083000
N	6.633512000	1.221130000	−0.526633000
N	4.267302000	2.351070000	−0.212440000
N	7.773475000	0.481078000	−0.623106000
H	7.615519000	−0.325470000	−0.608587000
O	6.553668000	−1.863873000	−0.153368000
O	2.539525000	−0.277303000	1.705970000
O	9.317248000	2.144303000	−0.542848000
Pt	4.749976000	0.298124000	0.027186000

H---F interactions			
C	−7.157681000	1.841764000	−0.013711000
C	−7.091370000	3.304455000	0.318393000
H	−7.494471000	3.458164000	1.197862000
H	−7.579714000	3.815614000	−0.359345000
H	−6.153662000	3.593512000	0.332994000
C	−9.913458000	2.383472000	0.246014000
C	−10.486156000	3.029521000	−0.985778000
H	−10.906682000	2.344835000	−1.547386000
H	−9.767713000	3.467253000	−1.488021000
H	−11.155171000	3.695306000	−0.724581000
C	−8.717554000	−2.197556000	−0.255537000
H	−8.409562000	−3.089846000	−0.358996000
C	−10.129673000	−1.892854000	−0.126012000
C	−11.088501000	−2.899267000	−0.113419000
H	−10.832910000	−3.810627000	−0.196148000
C	−12.419613000	−2.552679000	0.022723000
H	−13.092558000	−3.223321000	0.029907000
C	−12.760494000	−1.226320000	0.147563000
H	−13.670342000	−0.969834000	0.236316000
C	−11.760473000	−0.272381000	0.141103000
H	−12.001602000	0.641185000	0.237423000
C	−5.816872000	−2.459960000	−0.056766000
C	−4.335198000	−2.289497000	−0.049936000
C	−3.674856000	−1.140688000	−0.484624000
H	−4.176695000	−0.406997000	−0.818381000
C	−2.296233000	−1.058339000	−0.433475000
H	−1.844824000	−0.272054000	−0.715688000
C	−1.592961000	−2.149017000	0.038808000
C	−2.203226000	−3.303301000	0.465413000
H	−1.691442000	−4.039474000	0.778959000
C	−3.580488000	−3.363799000	0.425792000
H	−4.023000000	−4.149487000	0.726542000
F	−0.249248000	−2.069447000	0.120748000
N	−7.897573000	−1.194928000	−0.222929000
N	−10.457189000	−0.586012000	0.005142000
N	−6.544002000	−1.326699000	−0.306502000
H	−6.173726000	−0.602076000	−0.422695000
O	−6.065211000	1.314912000	−0.300999000
O	−10.196135000	2.836045000	1.345000000
O	−6.345100000	−3.538764000	0.165931000
Pt	−8.814818000	0.758443000	−0.000755000
C	8.234838000	2.428556000	−0.011035000
C	8.301149000	3.891245000	0.321070000
H	7.898048000	4.044954000	1.200539000
H	7.812806000	4.402405000	−0.356668000
H	9.238857000	4.180303000	0.335671000
C	5.479060000	2.970264000	0.248691000
C	4.906363000	3.616311000	−0.983101000
H	4.485837000	2.931626000	−1.544709000
H	5.624805000	4.054044000	−1.485344000
H	4.237348000	4.282097000	−0.721904000

C	6.674965000	−1.610764000	−0.252860000
H	6.982956000	−2.503056000	−0.356319000
C	5.262847000	−1.306063000	−0.123335000
C	4.304018000	−2.312475000	−0.110743000
H	4.559609000	−3.223835000	−0.193471000
C	2.972907000	−1.965888000	0.025401000
H	2.299962000	−2.636530000	0.032584000
C	2.632026000	−0.639530000	0.150240000
H	1.722178000	−0.383043000	0.238993000
C	3.632046000	0.314410000	0.143780000
H	3.390916000	1.227976000	0.240100000
C	9.575648000	−1.873170000	−0.054089000
C	11.057321000	−1.702707000	−0.047258000
C	11.717663000	−0.553897000	−0.481947000
H	11.215825000	0.179794000	−0.815703000
C	13.096287000	−0.471548000	−0.430798000
H	13.547694000	0.314737000	−0.713011000
C	13.799558000	−1.562225000	0.041485000
C	13.189293000	−2.716510000	0.468090000
H	13.701076000	−3.452684000	0.781637000
C	11.812032000	−2.777008000	0.428469000
H	11.369519000	−3.562696000	0.729220000
F	15.143270000	−1.482656000	0.123425000
N	7.494946000	−0.608138000	−0.220252000
N	4.935330000	0.000779000	0.007819000
N	8.848516000	−0.739908000	−0.303825000
H	9.218793000	−0.015285000	−0.420017000
O	9.327307000	1.901703000	−0.298322000
O	5.196384000	3.422837000	1.347677000
O	9.047420000	−2.951973000	0.168608000
Pt	6.577701000	1.345235000	0.001922000
O---H interactions			
C	5.738979000	2.827397000	2.236787000
C	6.891927000	3.737694000	2.546987000
H	6.744833000	4.607562000	2.121222000
H	7.720737000	3.342536000	2.206375000
H	6.961653000	3.856372000	3.518644000
C	6.648332000	3.333407000	−0.384709000
C	7.841699000	2.492231000	−0.746609000
H	7.544668000	1.703220000	−1.246883000
H	8.299077000	2.206622000	0.071468000
H	8.456658000	3.017803000	−1.298583000
C	2.543745000	0.949421000	−0.015206000
H	1.732089000	0.478623000	0.130771000
C	3.035334000	1.216772000	−1.353325000
C	2.319703000	0.824223000	−2.478561000
H	1.492683000	0.365162000	−2.388846000
C	2.830172000	1.113744000	−3.730006000
H	2.360385000	0.851610000	−4.513110000
C	4.025933000	1.785381000	−3.830365000
H	4.394064000	1.987525000	−4.681895000
C	4.684331000	2.160977000	−2.674717000

H	5.504918000	2.633079000	−2.750746000
C	1.666280000	0.974200000	2.768993000
C	1.528212000	1.027670000	4.253089000
C	2.602245000	1.100703000	5.139592000
H	3.490220000	1.111136000	4.803542000
C	2.387431000	1.159389000	6.503561000
H	3.116343000	1.223293000	7.108756000
C	1.085507000	1.122873000	6.962779000
C	0.000226000	1.039408000	6.124934000
H	−0.883566000	1.009412000	6.471629000
C	0.229488000	1.000283000	4.765527000
H	−0.508248000	0.953334000	4.167936000
F	0.862306000	1.208947000	8.289950000
N	3.264296000	1.383703000	0.970136000
N	4.208526000	1.885801000	−1.444560000
N	2.919127000	1.231118000	2.279364000
H	3.540499000	1.421524000	2.782679000
O	5.176443000	2.329212000	3.231253000
O	6.653672000	4.525400000	−0.652333000
O	0.716266000	0.735541000	2.038958000
Pt	5.089999000	2.414334000	0.412490000
C	−5.738979000	−2.827397000	−2.236787000
C	−6.891927000	−3.737694000	−2.546987000
H	−6.744833000	−4.607562000	−2.121222000
H	−7.720737000	−3.342536000	−2.206375000
H	−6.961653000	−3.856372000	−3.518644000
C	−6.648332000	−3.333407000	0.384709000
C	−7.841699000	−2.492231000	0.746609000
H	−7.544668000	−1.703220000	1.246883000
H	−8.299077000	−2.206622000	−0.071468000
H	−8.456658000	−3.017803000	1.298583000
C	−2.543745000	−0.949421000	0.015206000
H	−1.732089000	−0.478623000	−0.130771000
C	−3.035334000	−1.216772000	1.353325000
C	−2.319703000	−0.824223000	2.478561000
H	−1.492683000	−0.365162000	2.388846000
C	−2.830172000	−1.113744000	3.730006000
H	−2.360385000	−0.851610000	4.513110000
C	−4.025933000	−1.785381000	3.830365000
H	−4.394064000	−1.987525000	4.681895000
C	−4.684331000	−2.160977000	2.674717000
H	−5.504918000	−2.633079000	2.750746000
C	−1.666280000	−0.974200000	−2.768993000
C	−1.528212000	−1.027670000	−4.253089000
C	−2.602245000	−1.100703000	−5.139592000
H	−3.490220000	−1.111136000	−4.803542000
C	−2.387431000	−1.159389000	−6.503561000
H	−3.116343000	−1.223293000	−7.108756000
C	−1.085507000	−1.122873000	−6.962779000
C	−0.000226000	−1.039408000	−6.124934000
H	0.883566000	−1.009412000	−6.471629000
C	−0.229488000	−1.000283000	−4.765527000

H	0.508248000	−0.953334000	−4.167936000
F	−0.862306000	−1.208947000	−8.289950000
N	−3.264296000	−1.383703000	−0.970136000
N	−4.208526000	−1.885801000	1.444560000
N	−2.919127000	−1.231118000	−2.279364000
H	−3.540499000	−1.421524000	−2.782679000
O	−5.176443000	−2.329212000	−3.231253000
O	−6.653672000	−4.525400000	0.652333000
O	−0.716266000	−0.735541000	−2.038958000
Pt	−5.089999000	−2.414334000	−0.412490000
C	14.176179000	2.827397000	2.236787000
C	15.329127000	3.737694000	2.546987000
H	15.182033000	4.607562000	2.121222000
H	16.157937000	3.342536000	2.206375000
H	15.398853000	3.856372000	3.518644000
C	15.085532000	3.333407000	−0.384709000
C	16.278899000	2.492231000	−0.746609000
H	15.981868000	1.703220000	−1.246883000
H	16.736277000	2.206622000	0.071468000
H	16.893858000	3.017803000	−1.298583000
C	10.980945000	0.949421000	−0.015206000
H	10.169289000	0.478623000	0.130771000
C	11.472534000	1.216772000	−1.353325000
C	10.756903000	0.824223000	−2.478561000
H	9.929883000	0.365162000	−2.388846000
C	11.267372000	1.113744000	−3.730006000
H	10.797585000	0.851610000	−4.513110000
C	12.463133000	1.785381000	−3.830365000
H	12.831264000	1.987525000	−4.681895000
C	13.121531000	2.160977000	−2.674717000
H	13.942118000	2.633079000	−2.750746000
C	10.103480000	0.974200000	2.768993000
C	9.965412000	1.027670000	4.253089000
C	11.039445000	1.100703000	5.139592000
H	11.927420000	1.111136000	4.803542000
C	10.824631000	1.159389000	6.503561000
H	11.553543000	1.223293000	7.108756000
C	9.522707000	1.122873000	6.962779000
C	8.437426000	1.039408000	6.124934000
H	7.553634000	1.009412000	6.471629000
C	8.666688000	1.000283000	4.765527000
H	7.928952000	0.953334000	4.167936000
F	9.299506000	1.208947000	8.289950000
N	11.701496000	1.383703000	0.970136000
N	12.645726000	1.885801000	−1.444560000
N	11.356327000	1.231118000	2.279364000
H	11.977699000	1.421524000	2.782679000
O	13.613643000	2.329212000	3.231253000
O	15.090872000	4.525400000	−0.652333000
O	9.153466000	0.735541000	2.038958000
Pt	13.527199000	2.414334000	0.412490000
O---H interactions			

C	5.738979000	2.827397000	2.236787000
C	6.891927000	3.737694000	2.546987000
H	6.744833000	4.607562000	2.121222000
H	7.720737000	3.342536000	2.206375000
H	6.961653000	3.856372000	3.518644000
C	6.648332000	3.333407000	−0.384709000
C	7.841699000	2.492231000	−0.746609000
H	7.544668000	1.703220000	−1.246883000
H	8.299077000	2.206622000	0.071468000
H	8.456658000	3.017803000	−1.298583000
C	2.543745000	0.949421000	−0.015206000
H	1.732089000	0.478623000	0.130771000
C	3.035334000	1.216772000	−1.353325000
C	2.319703000	0.824223000	−2.478561000
H	1.492683000	0.365162000	−2.388846000
C	2.830172000	1.113744000	−3.730006000
H	2.360385000	0.851610000	−4.513110000
C	4.025933000	1.785381000	−3.830365000
H	4.394064000	1.987525000	−4.681895000
C	4.684331000	2.160977000	−2.674717000
H	5.504918000	2.633079000	−2.750746000
C	1.666280000	0.974200000	2.768993000
C	1.528212000	1.027670000	4.253089000
C	2.602245000	1.100703000	5.139592000
H	3.490220000	1.111136000	4.803542000
C	2.387431000	1.159389000	6.503561000
H	3.116343000	1.223293000	7.108756000
C	1.085507000	1.122873000	6.962779000
C	0.000226000	1.039408000	6.124934000
H	−0.883566000	1.009412000	6.471629000
C	0.229488000	1.000283000	4.765527000
H	−0.508248000	0.953334000	4.167936000
F	0.862306000	1.208947000	8.289950000
N	3.264296000	1.383703000	0.970136000
N	4.208526000	1.885801000	−1.444560000
N	2.919127000	1.231118000	2.279364000
H	3.540499000	1.421524000	2.782679000
O	5.176443000	2.329212000	3.231253000
O	6.653672000	4.525400000	−0.652333000
O	0.716266000	0.735541000	2.038958000
Pt	5.089999000	2.414334000	0.412490000
C	15.406499000	3.693353000	−5.366160000
C	16.559446000	2.783056000	−5.055960000
H	16.412352000	1.913188000	−5.481725000
H	17.388256000	3.178214000	−5.396572000
H	16.629172000	2.664378000	−4.084303000
C	16.315852000	3.187343000	−7.987656000
C	17.509218000	4.028519000	−8.349557000
H	17.212187000	4.817530000	−8.849831000
H	17.966596000	4.314128000	−7.531480000
H	18.124177000	3.502947000	−8.901531000
C	12.211265000	5.571329000	−7.618153000

H	11.399608000	6.042127000	−7.472177000
C	12.702854000	5.303978000	−8.956272000
C	11.987223000	5.696527000	−10.081508000
H	11.160203000	6.155588000	−9.991793000
C	12.497691000	5.407006000	−11.332953000
H	12.027904000	5.669140000	−12.116057000
C	13.693452000	4.735369000	−11.433312000
H	14.061583000	4.533225000	−12.284842000
C	14.351851000	4.359773000	−10.277664000
H	15.172438000	3.887671000	−10.353694000
C	11.333799000	5.546550000	−4.833954000
C	11.195731000	5.493080000	−3.349859000
C	12.269764000	5.420047000	−2.463355000
H	13.157739000	5.409614000	−2.799405000
C	12.054951000	5.361361000	−1.099386000
H	12.783863000	5.297457000	−0.494192000
C	10.753027000	5.397877000	−0.640168000
C	9.667745000	5.481342000	−1.478013000
H	8.783953000	5.511338000	−1.131319000
C	9.897007000	5.520467000	−2.837420000
H	9.159271000	5.567416000	−3.435012000
F	10.529825000	5.311803000	0.687002000
N	12.931815000	5.137047000	−6.632811000
N	13.876045000	4.634949000	−9.047507000
N	12.586646000	5.289632000	−5.323584000
H	13.208018000	5.099226000	−4.820269000
O	14.843962000	4.191538000	−4.371695000
O	16.321192000	1.995349000	−8.255280000
O	10.383785000	5.785209000	−5.563989000
Pt	14.757519000	4.106416000	−7.190457000
C	2.698221000	−2.827397000	−2.236787000
C	1.545273000	−3.737694000	−2.546987000
H	1.692367000	−4.607562000	−2.121222000
H	0.716463000	−3.342536000	−2.206375000
H	1.475547000	−3.856372000	−3.518644000
C	1.788868000	−3.333407000	0.384709000
C	0.595501000	−2.492231000	0.746609000
H	0.892532000	−1.703220000	1.246883000
H	0.138123000	−2.206622000	−0.071468000
H	−0.019458000	−3.017803000	1.298583000
C	5.893455000	−0.949421000	0.015206000
H	6.705111000	−0.478623000	−0.130771000
C	5.401866000	−1.216772000	1.353325000
C	6.117497000	−0.824223000	2.478561000
H	6.944517000	−0.365162000	2.388846000
C	5.607028000	−1.113744000	3.730006000
H	6.076815000	−0.851610000	4.513110000
C	4.411267000	−1.785381000	3.830365000
H	4.043136000	−1.987525000	4.681895000
C	3.752869000	−2.160977000	2.674717000
H	2.932282000	−2.633079000	2.750746000
C	6.770920000	−0.974200000	−2.768993000

C	6.908988000	−1.027670000	−4.253089000
C	5.834955000	−1.100703000	−5.139592000
H	4.946980000	−1.111136000	−4.803542000
C	6.049769000	−1.159389000	−6.503561000
H	5.320857000	−1.223293000	−7.108756000
C	7.351693000	−1.122873000	−6.962779000
C	8.436974000	−1.039408000	−6.124934000
H	9.320766000	−1.009412000	−6.471629000
C	8.207712000	−1.000283000	−4.765527000
H	8.945448000	−0.953334000	−4.167936000
F	7.574894000	−1.208947000	−8.289950000
N	5.172904000	−1.383703000	−0.970136000
N	4.228674000	−1.885801000	1.444560000
N	5.518073000	−1.231118000	−2.279364000
H	4.896701000	−1.421524000	−2.782679000
O	3.260757000	−2.329212000	−3.231253000
O	1.783528000	−4.525400000	0.652333000
O	7.720934000	−0.735541000	−2.038958000
Pt	3.347201000	−2.414334000	−0.412490000
C	4.508660000	3.693353000	9.839734000
C	5.661608000	2.783056000	10.149935000
H	5.514513000	1.913188000	9.724170000
H	6.490417000	3.178214000	9.809323000
H	5.731334000	2.664378000	11.121591000
C	5.418013000	3.187343000	7.218238000
C	6.611380000	4.028519000	6.856338000
H	6.314348000	4.817530000	6.356064000
H	7.068758000	4.314128000	7.674415000
H	7.226338000	3.502947000	6.304364000
C	1.313426000	5.571329000	7.587741000
H	0.501769000	6.042127000	7.733718000
C	1.805015000	5.303978000	6.249623000
C	1.089384000	5.696527000	5.124386000
H	0.262364000	6.155588000	5.214101000
C	1.599852000	5.407006000	3.872941000
H	1.130065000	5.669140000	3.089838000
C	2.795613000	4.735369000	3.772582000
H	3.163744000	4.533225000	2.921052000
C	3.454012000	4.359773000	4.928230000
H	4.274599000	3.887671000	4.852201000
C	0.435961000	5.546550000	10.371941000
C	0.297892000	5.493080000	11.856036000
C	1.371925000	5.420047000	12.742540000
H	2.259901000	5.409614000	12.406489000
C	1.157112000	5.361361000	14.106508000
H	1.886024000	5.297457000	14.711703000
C	−0.144812000	5.397877000	14.565726000
C	−1.230094000	5.481342000	13.727882000
H	−2.113886000	5.511338000	14.074576000
C	−1.000832000	5.520467000	12.368475000
H	−1.738568000	5.567416000	11.770883000
F	−0.368014000	5.311803000	15.892897000

N	2.033976000	5.137047000	8.573083000
N	2.978206000	4.634949000	6.158387000
N	1.688808000	5.289632000	9.882311000
H	2.310179000	5.099227000	10.385626000
O	3.946124000	4.191538000	10.834200000
O	5.423353000	1.995350000	6.950614000
O	−0.514054000	5.785209000	9.641906000
Pt	3.859680000	4.106416000	8.015438000

Two colored properties named shape index and curvedness plots which have importance in the description of the stacking of ring systems [1] are shown in Figure S2. The first evidence on the possibility of ring stacking is the presence of large green area separated by blue edges in the curvedness plot. In this plot, green color indicates flat regions while the dark blue means large positive curvature. The second evidence comes from the shape index plot on the HF surface. In this plot, the presence of complementary hollows (red triangle) and bumps (blue triangle) where two molecular surfaces touch one another is an indicator on the ring stacking. Figures S2 and S3 showed the shape index and curvedness plots of the studied Pt-complexes. These colored properties were used to characterize the packing modes, in particular planar stacking arrangements, and showed how the adjacent molecules contact one another. For complex **1**, the presence of large flat area in the curvedness plot together with the small red and blue triangles in the shape index graph of both complex sides are good indication on the presence of ring stacking. In the current case, it is not due to the usual π–π stacking interactions between two aromatic π-systems. Here the interaction occurs between the pyridine ring π-system with the chelate five membered "CNPtNC" ring. The centroid-centroid distances between the two rings were found in the range 3.558–3.569° which are very close to the known limit of the well known π–π stacking between the aromatic six member ring [2]. In complex **4**, the two sides of the complex showed two different interactions with the neighboring complex units. In one side of the complex, the chelate ring stack with another similar side from another complex unit with very short centroid-centroid distance of 3.387 Å. The other side is included in the same kind of stacking as in **1** with centroid-centroid distance of 3.710 Å. In complex **3**, although there is no aromatic ring in the structure but we note the large flat area in the curvedness plot as well as the small red and blue triangles in the shape index plot of one side. These observations reveal the presence of ring stacking between the two chelate rings lying above each other. The centroid-centroid distance between the two chelate rings is 3.621 Å. Although the two complex units of **5** differ very slightly in the geometric parameters but based on the curvedness plot they are included in two different types of packing with the neighboring molecules. Only one flat side for unit 1while the two sides of unit 2 have large flat region are indicative on the presence of planar stacking. In complex **2**, the curvedness plot and shape index map not gave any indication on the presence of planar stacking between the complex units in the crystal lattice.

References

1. Koenderink, J.J.; van Doorn, A.J. Surface shape and curvature scales.*Image Visn. Comput.***1992**,*10*, 557–564.
2. Janiak, C. A critical account on π–π stacking in metal complexes with aromatic nitrogen-containing ligands. *J. Chem. Soc. Dalton Trans.* **2000**, 3885–3896, doi:10.1039/B003010O.
